# Transformation of 3-(Furan-2-yl)-1,3-di(het)arylpropan-1-ones to Prop-2-en-1-ones via Oxidative Furan Dearomatization/2-Ene-1,4,7-triones Cyclization

**DOI:** 10.3390/molecules26092637

**Published:** 2021-04-30

**Authors:** Roman O. Shcherbakov, Diana A. Eshmemet’eva, Anton A. Merkushev, Igor V. Trushkov, Maxim G. Uchuskin

**Affiliations:** 1 Department of Chemistry, Perm State University, Bukireva St. 15, 614990 Perm, Russia; romanshcherbakov00@gmail.com (R.O.S.); diana.ttt.00@mail.ru (D.A.E.); anton.merckushev@psu.ru (A.A.M.); 2 N.D. Zelinsky Institute of Organic Chemistry Russian Academy of Sciences, Leninsky Pr. 47, 119334 Moscow, Russia; itrushkov@mail.ru; 3 D. Rogachev National Medical Research Center of Pediatric Hematology, Oncology and Immunology, Samory Mashela St. 1, 117997 Moscow, Russia

**Keywords:** furan, oxidation, cyclization, 2-ene-1,4,7-trione, Paal-Knorr reaction

## Abstract

The approach to 3-(furan-2-yl)-1,3-di(het)arylprop-2-en-1-ones based on the oxidative dearomatization of 3-(furan-2-yl)-1,3-di(het)arylpropan-1-ones followed by an unusual cyclization of the formed di(het)aryl-substituted 2-ene-1,4,7-triones has been developed. The cyclization step is related to the Paal–Knorr synthesis, but the furan ring formation is accompanied in this case by a formal shift of the double bond through the formation of a fully conjugated 4,7-hydroxy-2,4,6-trien-1-one system or its surrogate.

## 1. Introduction

**Figure 1:**
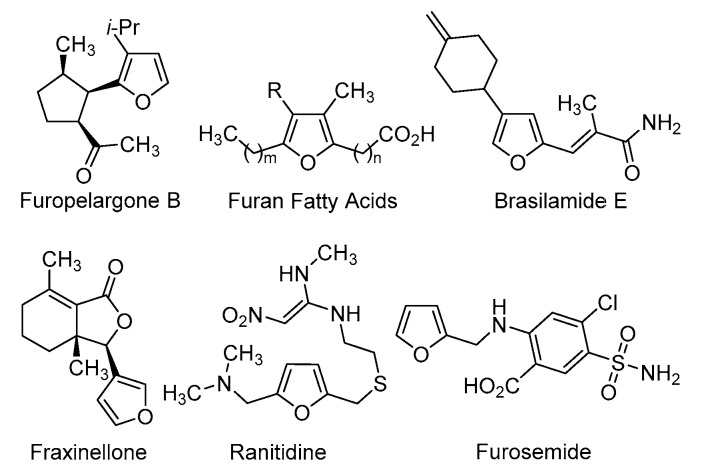
Some furan-based medicines and natural products.

Substituted furans play an important role in modern organic and medicinal chemistry. At first, the furan core is an integral part of diverse plant metabolites and, accordingly, a lot of them were isolated from various natural sources; some of them exhibit multifaceted biological activities. Thus, furopelargone B was isolated from several species of the *Alpinia* genus (Figure 1) [1,2,3]. The furan fatty acids are important natural compounds due to their health benefits and impact on inflammatory and cardiovascular diseases [4,5]. Brasilamide E, isolated from the plant endophytic fungus *Paraconiothynium brasiliense*, selectively inhibited the proliferation of the breast and gastric cancer cell lines [6]. Fraxinellone, a partially degraded limonoid isolated from several plants [7,8,9], exhibits antifertility, vascular relaxing, anti-inflammatory, insecticidal activities [10,11,12]. Secondly, there are approved drugs containing the furan ring. For example, ranitidine is a commonly used histamine H_2_-receptor antagonist, which helps to prevent and treat gastric acid-related conditions, including ulcers [13]. Furosemide is a potent loop diuretic that is used for edema secondary to congestive heart failure exacerbation, liver and kidney failure, and high blood pressure [14,15]. Moreover, due to their versatile reactivity, furans are intensively used in organic synthesis as universal building blocks for the preparation of various useful products, including natural compounds [16,17,18,19,20].

**Scheme 1:**
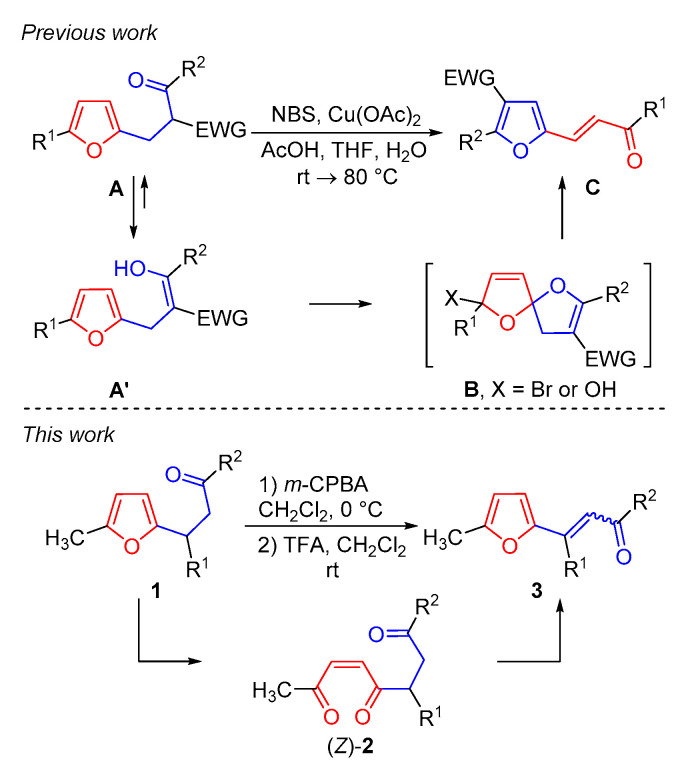
Previous approach and concept of this work.

The development of methodologies for the synthesis of substituted furans is the focus of many research groups [21,22,23,24]. In 2013, Yin et al. described an original approach for the synthesis of substituted 4-(furan-2-yl)but-3-en-2-ones **C** based on the oxidative rearrangement of 4-(furan-2-yl)butan-2-ones **A** (Scheme 1) [25]. The process proceeds through the formation of a key spiro-intermediate **B**, the hydrolysis of which results in the formation of functionalized furans in moderate to good yields. The formation of intermediate **B** is driven by the presence of electron-withdrawing group (EWG) at the α-position to the ketone moiety, that facilitates its enolization and subsequent nucleophilic attack onto the activated furan nucleus.

Based on our experience on the use of furans dearomatization in the synthesis of various heterocycles [26,27,28,29,30,31], we assumed that the removal of EWG may lead to a switch of reactivity pattern via the crucial decrease of the enol **A**′ form contribution. As a result, the oxidation of oxoalkyl furans, which lack α-EWG-functionality, may lead to different products. To test this hypothesis, we studied the oxidation of 2-(2-furyl)-2-phenylethyl ketones **1** as convenient model substrates. With this goal, we synthesized a series of these starting compounds using Michael addition of 2-substituted furans to α,β-unsaturated carbonyl compounds [32]. Indeed, we found that the oxidation of substrates **1** followed by treatment with trifluoroacetic acid (TFA) led to unsaturated ketones **3** through the intermediate formation of unsaturated 1,4,7-triketones **2**. Herein, we report the results of our investigation.

## 2. Results and Discussions

**Scheme 2:**
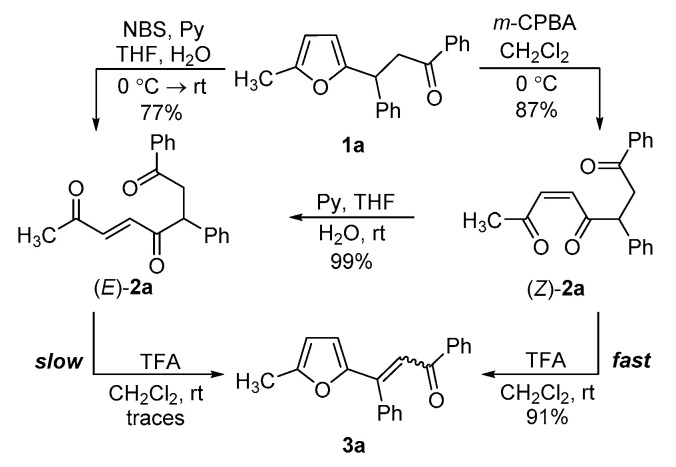
Synthesis of (*E*)-**2a**, (*Z*)-**2a** and **3a.**

We started this study by searching for optimal reaction conditions for oxidation of model furan **1a**. Initially we screened a series of oxidants, commonly applied for performing related processes, and found that the use of *N*-bromosuccinimide (NBS)/pyridine system in aq. THF leads to the formation of (*E*)-**2a** with 77% yield while application of *m*-chloroperbenzoic acid (*m*-CPBA) afforded (*Z*)-**2a** [33] as the exclusive product in 87% yield (Scheme 2) [34,35]. Other oxidants (ceric ammonium nitrate, pyridinium chlorochromate, 2,3-dichloro-5,6-dicyanobenzoquinone, Oxone, NaClO_2_, MnO_2_, and Pb(OAc)_4_) led to similar results, but with a lower conversion of the starting compound **1a** or in low yield and poor *Z*,*E*-ratio of triketone **2a**.

It is noteworthy that enetriketones **2**, containing several electrophilic and nucleophilic sites with different reactivity, are attractive objects for designing various transformations, including condensations, which could afford diverse alicyclic or heterocyclic products. We attempted to study the chemical behavior of triketone **2a** under various conditions. Basic conditions were screened first. We found that the treatment of the starting (*Z*)-**2a** with pyridine in aq. THF leads to a quantitative isomerization to (*E*)-**2a**. On the other hand, we did not observe any conversion of formed (*E*)-**2a**. Similar results were achieved when we used PPh_3_ in toluene or 4-(dimethylamino)pyridine in DMF.

On the other hand, it is well known that aldol condensation, Paal–Knorr reaction and many other processes could be initiated by various Brønsted acids; therefore, we studied acid-catalyzed transformations of **2a**. We found that the treatment of (*Z*)-**2a** with TFA in CH_2_Cl_2_ at room temperature unexpectedly leads to the rapid formation of furan **3a**. Oppositely, (*E*)-**2a** transforms into furan **3a** only in trace amounts under the same conditions. We believe that the geometry of the C=C bond and the mutual arrangement of carbonyl groups alter the reactivity dramatically. A wide range of tested Brønsted acids led to a similar result, but after the prolonged reaction time and with lower yield of the desired product **3a**. Under the optimal reaction conditions, the product was obtained as a mixture of (*Z*)*-* and (*E*)-isomers in a ratio of *ca* 89:11 based on NMR analysis.

**Scheme 3:**
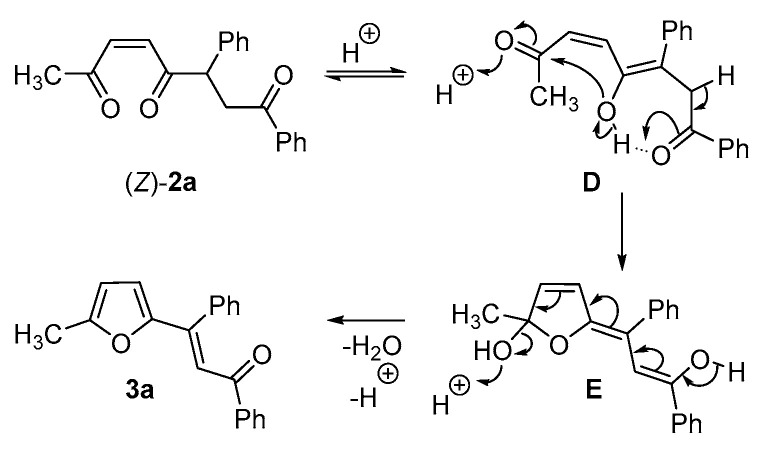
The plausible mechanism of cyclodehydration of (*Z*)-**2a** with formation of **3a.**

The plausible mechanism of this unusual transformation is presented in Scheme 3. We assumed that hydroxy group of enol **D**, which is presumably formed in an acidic media, attacks a suitably located carbonyl carbon with the formation of an intermediate 2,5-dihydrofuran-2-ol **E**, which then is converted into the desired product **3a** via dehydration. The disclosed cyclodehydration is similar to the Paal–Knorr furan synthesis, wherein, however, saturated 1,4-dicarbonyl compounds are used as starting compounds. In our case the formation of the furan product proceeds through the cyclodehydration of unsaturated 1,4-diketone. Further water elimination leads to the product of the formal side chain oxidation, i.e., α,β-unsaturated ketone **3a** [36,37].

**Scheme 4:**
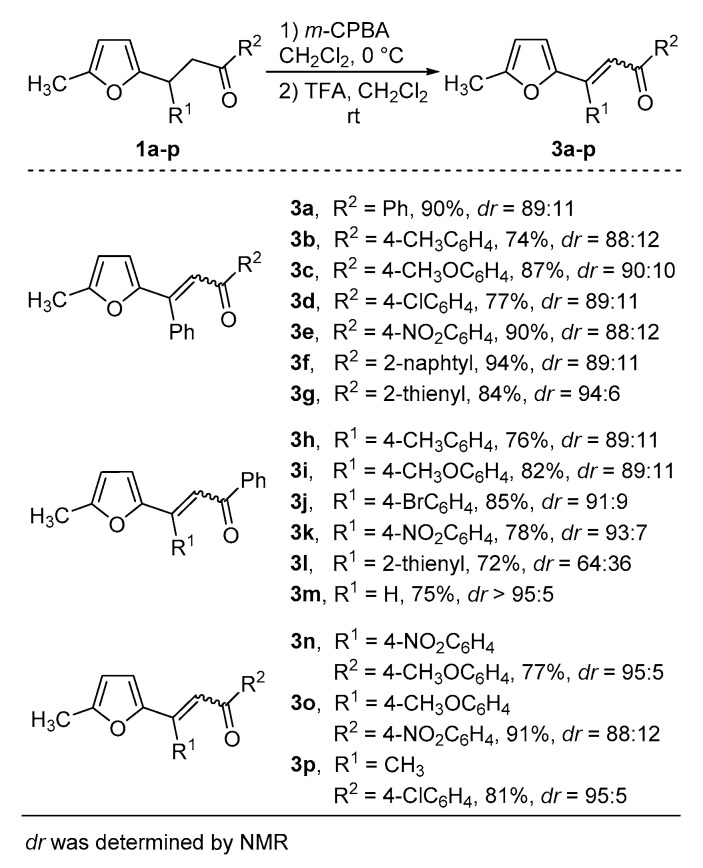
The scope of the one-pot synthesis of furans **1.**

Since we optimized both stages separately, we decided to realize a one-pot process. First, we treated the Michael adduct **1a** with *m*-CPBA at 0 °C in CH_2_Cl_2_, then we added TFA to the reaction mixture at ambient temperature that led to the formation of the desired product with 90% yield (Scheme 4). Encouraged by this result, we studied the scope of this synthetic protocol. We found that the wide range of triketones, formed through the oxidation of the corresponding Michael adducts **1a**–**p**, could be involved into the discussed cyclization. Such substituents at aromatic rings as alkyl, methoxy, halogen, nitro had no significant influence on the reaction efficiency, and the desired products **3a**–**e**,**h**–**k**,**n**–**p** were isolated in good to high yields. Moreover, we showed that the heterocyclic and naphthyl-containing Michael adducts **1f**,**g**,**l** could also be converted into the corresponding products. Unfortunately, we failed to separate (*Z*)- and (*E*)-isomers of the resulting mixture using column chromatography, and in addition, we were unable to improve the ratio of isomers using the known methods of isomerization of alkenes.

## 3. Materials and Methods

### 3.1. General Information 

^1^H and ^13^C NMR spectra were recorded with a “Bruker Avance III HD 400” (Bruker, Billerica, MA, USA) (400 MHz for ^1^H and 100 MHz for ^13^C NMR) spectrometer at room temperature; the chemical shifts (δ) were measured in ppm with respect to the solvent (CDCl_3_, ^1^H: δ = 7.26 ppm, ^13^C: δ = 77.16 ppm; (D_6_) DMSO, ^1^H: δ = 2.50 ppm, ^13^C: δ = 39.52 ppm). Coupling constants (*J*) are given in Hertz (Hz). Splitting patterns of an apparent multiplets associated with an averaged coupling constants were designated as s (singlet), d (doublet), t (triplet), q (quartet), m (multiplet), dd (doublet of doublets), and br (broadened). High resolution and accurate mass measurements were carried out using a micrOTOF-QTM ESI-TOF (electrospray ionization/time of flight, Bruker, Billerica, MA, USA) using ESI modes. GC/MS analysis was performed on an “Agilent 7890B” interfaced to an “Agilent 5977A” mass selective detector (Agilent Technologies, Santa Clara, CA, USA). Melting points were determined with a ”Stuart SMP 30” (Cole-Parmer, Stone, Staffordshire, UK). Column chromatography was performed on silica gel Macherey Nagel (40–63 μm, Macherey-Nagel GmbH & Co., Düren, Germany). All the reactions were carried out using freshly distilled and dry solvents from solvent stills. The NMR spectra for new compounds are available in the Supplementary Materials.

Starting 2-(3-oxoalkyl)furans **1** were synthesized according to the reported procedure [32]. CuBr_2_ (2.8 mg, 2.5 mol %) was added to a solution of corresponding chalcone (0.5 mmol) and 2-methylfuran (68 µL, 0.75 mmol) in CH_2_Cl_2_ (1.25 mL). The reaction mixture was stirred for 4 h at room temperature while controlling the reaction progress by TLC. Upon completion, the mixture was concentrated at reduced pressure. The product was isolated by column chromatography (silica gel, eluent—petroleum ether/CH_2_Cl_2_, gradient from 19:1 to 1:1). 

*3-(5-Methylfuran-2-yl)-1,3-diphenylpropan-1-one* (**1a**) [32]. Yield 122 mg (84%), pale yellow oil; ^1^H NMR (400 MHz, CDCl_3_) δ = 2.23 (s, 3H, CH_3_), 3.54 (dd, ^2^*J* = 16.9 Hz, ^3^*J* = 7.2 Hz, 1H, CH_2_), 3.80 (dd, ^2^*J* = 16.9 Hz, ^3^*J* = 7.2 Hz, 1H, CH_2_), 4.80 (t, ^3^*J* = 7.2 Hz, 1H, CH), 5.85 (d, ^3^*J* = 2.8 Hz, 1H, H_Fur_), 5.91 (d, ^3^*J* = 2.8 Hz, 1H, H_Fur_), 7.20–7.24 (m, 1H, H_Ar_), 7.29–7.35 (m, 4H, H_Ar_), 7.43–7.47 (m, 2H, H_Ar_), 7.53–7.57 (m, 1H, H_Ar_), 7.94–7.96 (m, 2H, H_Ar_) ppm; ^13^C NMR (100 MHz, CDCl_3_) δ = 13.6, 40.6, 43.9, 106.1, 106.6, 126.8, 128.0 (2C), 128.2 (2C), 128.6 (2C), 128.7 (2C), 133.1, 137.3, 142.4, 151.2, 155.1, 197.8 ppm.

*3-(5-Methylfuran-2-yl)-3-phenyl-1-(4-methylphenyl)propan-1-one (***1b***)* [32]. Yield 106 mg (70%), pale yellow oil; ^1^H NMR (400 MHz, CDCl_3_) δ = 2.21 (s, 3H, CH_3_), 2.40 (s, 3H, CH_3_), 3.50 (dd, ^2^*J* = 16.9 Hz, ^3^*J* = 7.2 Hz, 1H, CH_2_), 3.75 (dd, ^2^*J* = 16.9 Hz, ^3^*J* = 7.2 Hz, 1H, CH_2_), 4.77 (t, ^3^*J* = 7.2 Hz, 1H, CH), 5.83 (d, ^3^*J* = 3.0 Hz, 1H, H_Fur_), 5.89 (d, ^3^*J* = 3.0 Hz, 1H, H_Fur_), 7.20–7.25 (m, 3H, H_Ar_), 7.29–7.33 (m, 4H, H_Ar_), 7.84–7.86 (m, 2H, H_Ar_) ppm; ^13^C NMR (100 MHz, CDCl_3_) δ = 13.7, 21.7, 40.6, 43.7, 106.1, 106.5, 126.8, 128.0 (2C), 128.4 (2C), 128.6 (2C), 129.4 (2C), 134.7, 142.5, 144.0, 151.2, 155.1, 197.4 ppm.

*1-(4-Methoxyphenyl)-3-(5-methylfuran-2-yl)-3-phenylpropan-1-one (***1c***)* [32]. Yield 114 mg (71%), pale yellow oil; ^1^H NMR (400 MHz, CDCl_3_) δ = 2.24 (s, 3H, CH_3_), 3.49 (dd, ^2^*J* = 16.7 Hz, ^3^*J* = 7.2 Hz, 1H, CH_2_), 3.75 (dd, ^2^*J* = 16.7 Hz, ^3^*J* = 7.2 Hz, 1H, CH_2_), 3.88 (s, 3H, OCH_3_), 4.79 (t, ^3^*J* = 7.2 Hz, 1H, CH), 5.85 (br s, 1H, H_Fur_), 5.91 (br s, 1H, H_Fur_), 6.94 (d, ^3^*J* = 8.8 Hz, 2H, H_Ar_), 7.19–7.25 (m, 1H, H_Ar_), 7.27–7.35 (m, 4H, H_Ar_), 7.95 (d, 2H, ^3^*J* = 8.8 Hz, H_Ar_) ppm; ^13^C NMR (100 MHz, CDCl_3_) δ = 13.7, 40.7, 43.5, 55.6, 106.1, 106.5, 113.8 (2C), 126.8, 128.0 (2C), 128.6 (2C), 130.3, 130.5 (2C), 142.6, 151.1, 155.2, 163.6, 196.3 ppm.

*1-(4-Chlorophenyl)-3-(5-methylfuran-2-yl)-3-phenylpropan-1-one (***1d**) [32]. Yield 128 mg (79%), pale yellow oil; ^1^H NMR (400 MHz, CDCl_3_) δ = 2.22 (s, 3H, CH_3_), 3.50 (dd, ^2^*J* = 16.9 Hz, ^3^*J* = 7.2 Hz, 1H, CH_2_), 3.75 (dd, ^2^*J* = 16.9 Hz, ^3^*J* = 7.2 Hz, 1H, CH_2_), 4.77 (t, ^3^*J* = 7.2 Hz, 1H, CH), 5.85 (d, ^3^*J* = 2.6 Hz, 1H, H_Fur_), 5.89 (d, ^3^*J* = 2.6 Hz, 1H, H_Fur_), 7.20–7.23 (m, 1H, H_Ar_), 7.28–7.33 (m, 4H, H_Ar_), 7.41 (AA’BB’-system, ^3^*J* = 8.4 Hz, 2H, H_Ar_), 7.87 (AA’BB’-system, ^3^*J* = 8.4 Hz, 2H, H_Ar_) ppm; ^13^C NMR (100 MHz, CDCl_3_) δ = 13.6, 40.7, 43.8, 106.2, 106.7, 126.9, 128.0 (2C), 128.7 (2C), 129.0 (2C), 129.6 (2C), 135.6, 139.6, 142.2, 151.2, 154.8, 196.6 ppm.

*3-(5-Methylfuran-2-yl)-1-(4-nitrophenyl)-3-phenylpropan-1-one* (**1e***)* [32]. Yield 136 mg (81%), yellow oil; ^1^H NMR (400 MHz, CDCl_3_) δ = 2.21 (s, 3H, CH_3_), 3.55 (dd, ^2^*J* = 16.9 Hz, ^3^*J* = 7.2 Hz, 1H, CH_2_), 3.82 (dd, ^2^*J* = 16.9 Hz, ^3^*J* = 7.2 Hz, 1H, CH_2_), 4.74 (t, ^3^*J* = 7.2 Hz, 1H, CH), 5.84 (d, ^3^*J* = 2.9 Hz, 1H, H_Fur_), 5.88 (d, ^3^*J* = 2.9 Hz, 1H, H_Fur_), 7.22–7.23 (m, 1H, H_Ar_), 7.28–7.31 (m, 4H, H_Ar_), 8.05 (AA’BB’-system, ^3^*J* = 8.7 Hz, 2H, H_Ar_), 8.28 (AA’BB’-system, ^3^*J* = 8.7 Hz, 2H, H_Ar_) ppm; ^13^C NMR (100 MHz, CDCl_3_) δ = 13.7, 40.7, 44.4, 106.2, 106.9, 123.9 (2C), 127.1, 127.9 (2C), 128.8 (2C), 129.2 (2C), 141.6, 141.8, 150.5, 151.4, 154.4, 196.6 ppm.

*3-(5-Methylfuran-2-yl)-1-(naphthalen-2-yl)-3-phenylpropan-1-one (***1f***).* Yield 95 mg (56%), pale yellow oil; ^1^H NMR (400 MHz, CDCl_3_) δ = 2.22 (s, 3H, CH_3_), 3.65 (dd, ^2^*J* = 16.7 Hz, ^3^*J* = 7.1 Hz, 1H, CH_2_), 3.92 (dd, ^2^*J* = 16.7 Hz, *^3^J =* 7.1 Hz, 1H, CH_2_), 4.84 (t, ^3^*J* = 7.1 Hz, 1H, CH), 5.84 (d, ^3^*J* = 2.6 Hz, 1H, H_Fur_), 5.93 (d, ^3^*J* = 2.6 Hz, 1H, H_Fur_), 7.20–7.23 (m, 1H, H_Ar_), 7.29–7.32 (m, 2H, H_Ar_), 7.35–7.37 (m, 2H, H_Ar_), 7.53–7.61 (m, 2H, H_Ar_), 7.86–7.88 (m, 2H, H_Ar_), 7.94–7.96 (m, 1H, H_Ar_), 7.99–8.02 (m, 1H, H_Ar_), 8.45 (s, 1H, H_Ar_) ppm; ^13^C NMR (100 MHz, CDCl_3_) δ = 13.7, 40.9, 44.0, 106.2, 106.7, 124.1, 126.9 (2C), 127.9, 128.1 (2C), 128.5, 128.6, 128.7 (2C), 129.7, 129.9, 132.7, 134.6, 135.8, 142.5, 151.2, 155.1, 197.8 ppm; HRMS (ESI) calcd. for C_24_H_21_O_2_^+^ [M + H]^+^ 341.1536, found 341.1538.

*3-(5-Methylfuran-2-yl)-3-phenyl-1-(thiophen-2-yl)propan-1-one (***1g***).* Yield 110 mg (74%), pale yellow oil; ^1^H NMR (400 MHz, CDCl_3_) δ = 2.21 (s, 3H, CH_3_), 3.44 (dd, ^2^*J* = 16.2 Hz, ^3^*J* = 7.3 Hz, 1H, CH_2_), 3.69 (dd, ^2^*J* = 16.2 Hz, ^3^*J* = 7.3 Hz, 1H, CH_2_), 4.75 (t, ^3^*J* = 7.3 Hz, 1H, CH), 5.83 (d, ^3^*J* = 3.1 Hz, 1H, H_Fur_), 5.91 (d, ^3^*J* = 3.1 Hz, 1H, H_Fur_), 7.08–7.10 (m, 1H, H_Th_), 7.18–7.22 (m, 1H, H_Ar_), 7.29–7.32 (m, 4H, H_Ar_), 7.59–7.60 (m, 1H, H_Th_), 7.70–7.71 (m, 1H, H_Th_) ppm; ^13^C NMR (100 MHz, CDCl_3_) δ = 12.9, 40.2, 44.0, 105.4, 106.1, 126.2, 127.3 (2C), 127.4, 128.0 (2C), 131.2, 133.0, 141.4, 143.8, 150.6, 154.0, 189.9 ppm; HRMS (ESI) calcd. for C_18_H_17_SO_2_^+^ [M + H]^+^ 297.0944, found 297.0940.

*3-(5-Methylfuran-2-yl)-1-phenyl-3-(4-methylphenyl)propan-1-one (***1h***)* [32]. Yield 111 mg (73%), pale yellow oil; ^1^H NMR (400 MHz, CDCl_3_) δ = 2.22 (s, 3H CH_3_), 2.32 (s, 3H, CH_3_), 3.52 (dd, ^2^*J* = 16.9 Hz, ^3^*J* = 7.2 Hz, 1H, CH_2_), 3.78 (dd, ^2^*J* = 16.9 Hz, ^3^*J* = 7.2 Hz, 1H, CH_2_), 4.76 (t, ^3^*J* = 7.2 Hz, 1H, CH), 5.85 (d, ^3^*J* = 2.8 Hz, 1H, H_Fur_), 5.90 (d, ^3^*J* = 2.8 Hz, 1H, H_Fur_), 7.12 (AA’BB’-system, ^3^*J* = 7.8 Hz, 2H, H_Ar_), 7.22 (AA’BB’-system, ^3^*J* = 7.8 Hz, 2H, H_Ar_), 7.43–7.46 (m, 2H, H_Ar_), 7.53–7.57 (m, 1H, H_Ar_), 7.94–7.96 (m, 2H, H_Ar_) ppm; ^13^C NMR (100 MHz, CDCl_3_) δ = 13.7, 21.1, 40.3, 44.0, 106.1, 106.5, 127.9 (2C), 128.2 (2C), 128.7 (2C), 129.3 (2C), 133.1, 136.3, 137.3, 139.4, 151.1, 155.3, 197.9 ppm.

*3-(4-Methoxyphenyl)-3-(5-methylfuran-2-yl)-1-phenylpropan-1-one (***1i***)* [32]. Yield 120 mg (75%), pale yellow oil; ^1^H NMR (400 MHz, CDCl_3_) δ = 2.22 (s, 3H, CH_3_), 3.51 (dd, ^2^*J* = 16.8 Hz, ^3^*J* = 7.5 Hz, 1H, CH_2_), 3.72–3.77 (m, 1H, CH_2_), 3.77 (s, 3H, OCH_3_), 4.73 (t, ^3^*J* = 7.5 Hz, 1H, CH), 5.83 (d, ^3^*J* = 2.8 Hz, 1H, H_Fur_), 5.87 (d, ^3^*J* = 2.8 Hz, 1H, H_Fur_), 6.84 (AA’BB’-system, ^3^*J* = 8.6 Hz, 2H, H_Ar_), 7.24 (AA’BB’-system, ^3^*J* = 8.6 Hz, 2H, H_Ar_), 7.42–7.46 (m, 2H, H_Ar_), 7.53–7.57 (m, 1H, H_Ar_), 7.93–7.95 (m, 2H, H_Ar_) ppm; ^13^C NMR (100 MHz, CDCl_3_) δ = 13.7, 39.8, 44.0, 55.3, 106.1, 106.4, 114.1 (2C), 128.2 (2C), 128.7 (2C), 129.0 (2C), 133.1, 134.4, 137.2, 151.1, 155.4, 158.5, 198.0 ppm.

*3-(4-Bromophenyl)-3-(5-methylfuran-2-yl)-1-phenylpropan-1-one (***1j***)* [32]. Yield 157 mg (85%), orange oil; ^1^H NMR (400 MHz, CDCl_3_) δ = 2.24 (s, 3H, CH_3_), 3.55 (dd, ^2^*J* = 17.2 Hz, ^3^*J* = 7.7 Hz, 1H, CH_2_), 3.78 (dd, ^2^*J* = 17.2 Hz, ^3^*J* = 7.7 Hz, 1H, CH_2_), 4.78 (t, ^3^*J* = 7.7 Hz, 1H, CH), 5.87 (d, ^3^*J* = 2.6 Hz, 1H, H_Fur_), 5.93 (d, ^3^*J* = 2.6 Hz, 1H, H_Fur_), 7.23 (AA’BB’-system, ^3^*J* = 8.1 Hz, 2H, H_Ar_), 7.43 (AA’BB’-system, ^3^*J* = 8.1 Hz, 2H, H_Ar_), 7.45 (d, ^3^*J* = 7.5 Hz, 2H, H_Ar_), 7.56 (t, ^3^*J* = 7.5 Hz, 1H, H_Ar_), 7.96 (d, ^3^*J* = 7.5 Hz, 2H, H_Ar_) ppm; ^13^C NMR (100 MHz, CDCl_3_) δ = 13.6, 39.9, 43.5, 106.1, 106.7, 120.6, 128.1 (2C), 128.7 (2C), 129.8 (2C), 131.6 (2C), 133.2, 136.9, 141.3, 151.3, 154.4, 197.3 ppm.

*3-(5-Methylfuran-2-yl)-3-(4-nitrophenyl)-1-phenylpropan-1-one* (**1k***).* Yield 134 mg (80%), pale yellow oil; ^1^H NMR (400 MHz, CDCl_3_) δ = 2.23 (s, 3H, CH_3_), 3.61 (dd, ^2^*J* = 17.3 Hz, ^3^*J* = 7.3 Hz, 1H, CH_2_), 3.80 (dd, ^2^*J* = 17.3 Hz, ^3^*J* = 7.3 Hz, 1H, CH_2_), 4.88 (t, ^3^*J* = 7.3 Hz, 1H, CH), 5.87 (d, ^3^*J* = 3.1 Hz, 1H, H_Fur_), 5.96 (d, ^3^*J* = 3.1 Hz, 1H, H_Fur_), 7.43–7.47 (m, 2H, H_Ar_), 7.49 (AA’BB’-system, ^3^*J* = 8.7 Hz, 2H, H_Ar_), 7.55–7.58 (m, 1H, H_Ar_), 7.93–7.94 (m, 2H, H_Ar_), 8.14 (AA’BB’-system, ^3^*J* = 8.7 Hz, 2H, H_Ar_) ppm; ^13^C NMR (100 MHz, CDCl_3_) δ = 12.9, 39.6, 42.6, 105.6, 106.6, 123.2 (2C), 127.5 (2C), 128.1 (2C), 128.3 (2C), 132.8, 136.1, 146.3, 149.2, 151.2, 152.6, 196.2 ppm; HRMS (ESI) calcd. for C_20_H_18_NO_4_^+^ [M + H]^+^ 336.1230, found 336.1224.

*3-(5-Methylfuran-2-yl)-1-phenyl-3-(thiophen-2-yl)propan-1-one (***1l***).* Yield 101 mg (68%), pale yellow oil; ^1^H NMR (400 MHz, CDCl_3_) δ = 2.23 (s, 3H, CH_3_), 3.62 (dd, ^2^*J* = 16.9 Hz, ^3^*J* = 7.3 Hz, 1H, CH_2_), 3.77 (dd, ^2^*J* = 16.9 Hz, ^3^*J* = 7.3 Hz, 1H, CH_2_), 5.09 (t, ^3^*J* = 7.3 Hz, 1H, CH), 5.85 (d, ^3^*J* = 3.0 Hz 1H, H_Fur_), 5.98 (d, ^3^*J* = 3.0 Hz, 1H, H_Fur_), 6.89–6.91 (m, 2H, H_Ar_), 7.14 (d, ^3^*J* = 4.6 Hz, 1H, H_Th_), 7.43–7.47 (m, 2H, H_Ar+Th_), 7.55 (t, ^3^*J* = 4.6 Hz, 1H, H_Th_), 7.94–7.96 (m, 2H, H_Ar_) ppm; ^13^C NMR (100 MHz, CDCl_3_) δ = 13.7, 35.9, 44.8, 106.2, 106.7, 124.0, 124.9, 126.8, 128.3 (2C), 128.7 (2C), 133.3, 137.2, 145.9, 151.3, 154.3, 197.4 ppm; HRMS (ESI) calcd. for C_18_H_17_SO_2_^+^ [M + H]^+^ 297.0944, found 297.0942.

*3-(5-Methylfuran-2-yl)-1-phenylpropan-1-one (***1m***)* [38]. Yield 95 mg (89%), yellow oil; ^1^H NMR (400 MHz, CDCl_3_) δ = 2.24 (s, 3H, CH_3_), 3.04 (t, ^3^*J* = 7.6 Hz, 2H, CH_2_), 3.32 (t, ^3^*J* = 7.6 Hz, 2H, CH_2_), 5.85 (d, ^3^*J* = 3.1 Hz, 1H, H_Fur_), 5.92 (d, ^3^*J* = 3.1 Hz, 1H, H_Fur_), 7.45–7.47 (m, 2H, H_Ar_), 7.55–7.56 (m, 1H, H_Ar_), 7.96–7.98 (m, 2H, H_Ar_) ppm; ^13^C NMR (100 MHz, CDCl_3_) δ = 13.4, 22.6, 37.2, 105.9, 106.0, 128.0 (2C), 128.5 (2C), 133.0, 136.9, 150.5, 152.9, 198.8 ppm.

*1-(4-Methoxyphenyl)-3-(5-methylfuran-2-yl)-3-(4-nitrophenyl)propan-1-one (***1n***)* [32]. Yield 97 mg (53%), orange oil; ^1^H NMR (400 MHz, CDCl_3_) δ = 2.21 (s, 3H, CH_3_), 3.56 (dd, ^2^*J* = 17.2 Hz, ^3^*J* = 7.5 Hz, 1H, CH_2_), 3.73 (dd, ^2^*J* = 17.2 Hz, ^3^*J* = 7.5 Hz, 1H, CH_2_), 3.84 (s, 3H, OCH_3_), 4.86 (t, ^3^*J* = 7.5 Hz, 1H, CH), 5.86 (d, ^3^*J* = 2.9 Hz, 1H, H_Fur_), 5.96 (d, ^3^*J* = 2.9 Hz, 1H, H_Fur_), 6.91 (d, ^3^*J* = 8.8 Hz, 2H, H_Ar_), 7.47 (d, ^3^*J* = 8.7 Hz, 2H, H_Ar_), 7.91 (d, ^3^*J* = 8.8 Hz, 2H, H_Ar_), 8.11 (d, ^3^*J* = 8.7 Hz, 2H, H_Ar_) ppm; ^13^C NMR (100 MHz, CDCl_3_) δ = 13.6, 40.3, 42.7, 55.6, 106.3, 107.1, 113.9 (2C), 123.8 (2C), 129.0 (2C), 129.7, 130.4 (2C), 146.8, 150.0, 151.8, 153.4, 163.8, 195.3 ppm.

*3-(4-Methoxyphenyl)-3-(5-methylfuran-2-yl)-1-(4-nitrophenyl)propan-1-one (***1o***)* [32]. Yield 148 mg (81%), orange oil; ^1^H NMR (400 MHz, CDCl_3_) δ = 2.21 (s, 3H, CH_3_), 3.53 (dd, ^2^*J* = 16.9 Hz, ^3^*J* = 7.5 Hz, 1H, CH_2_), 3.75–3.81 (m, 1H, CH_2_), 3.77 (s, 3H, OCH_3_), 4.69 (t, ^3^*J* = 7.5 Hz, 1H, CH), 5.83–5.86 (m, 2H, H_Fur_), 6.83 (d, ^3^*J* = 8.6 Hz, 2H, H_Ar_), 7.23 (d, ^3^*J* = 8.6 Hz, 2H, H_Ar_), 8.05 (d, ^3^*J* = 8.8 Hz, 2H, H_Ar_), 8.27 (d, ^3^*J* = 8.8 Hz, 2H, H_Ar_) ppm; ^13^C NMR (100 MHz, CDCl_3_) δ = 13.7, 39.9, 44.6, 55.3, 106.2, 106.7, 114.2 (2C), 123.9 (2C), 128.9 (2C), 129.2 (2C), 133.8, 141.6, 150.4, 151.3, 154.8, 158.7, 196.7 ppm.

*1-(4-Chlorophenyl)-3-(5-methylfuran-2-yl)butan-1-one (***1p***).* Yield 105 mg (80%), pale yellow oil; ^1^H NMR (400 MHz, CDCl_3_) δ = 1.30 (d, ^3^*J* = 6.9 Hz, 3H, CH_3_), 2.22 (s, 3H, CH_3_), 3.01 (dd, ^2^*J* = 16.4 Hz, ^3^*J* = 8.2 Hz, 1H, CH_2_), 3.36 (dd, ^2^*J* = 16.4 Hz, *^3^J =* 5.4 Hz, 1H, CH_2_), 3.44–3.64 (m, 1H, CH), 5.82 (d, ^3^*J* = 3.0 Hz, 1H, H_Fur_), 5.88 (d, ^3^*J* = 3.0 Hz, 1H, H_Fur_), 7.42 (AA’BB’-system, ^3^*J* = 8.6 Hz, 2H, H_Ar_), 7.88 (AA’BB’-system, ^3^*J* = 8.6 Hz, 2H, H_Ar_) ppm; ^13^C NMR (100 MHz, CDCl_3_) δ = 13.6, 19.1, 29.5, 44.6, 104.7, 105.9, 129.0 (2C), 129.7 (2C), 135.7, 139.6, 150.6, 157.3, 197.8 ppm; HRMS (ESI) calcd. for C_15_H_16_ClO_2_^+^ [M + H]^+^ 263.0833, found 263.0835.

### 3.2. Synthesis of (Z)-1,3-diphenyloct-5-ene-1,4,7-trione (Z)-***2a***

To a solution of 3-(5-methylfuran-2-yl)-1,3-diphenylpropan-1-one **1a** (145 mg, 0.5 mmol) in CH_2_Cl_2_ (2.5 mL) was added *m*-CPBA (70% *w*/*w*, 148 mg, 0.6 mmol) at 0 °C. The reaction mixture was stirred at the same temperature for 1 h. Upon completion, the reaction mixture was poured into saturated solution of NaHCO_3_ (5 mL), extracted with CH_2_Cl_2_ (3 × 3 mL), washed with brine (3 × 1 mL), dried with anhydrous Na_2_SO_4_, and concentrated in vacuo. The product was purified by column chromatography (silica gel, eluent—petroleum ether/ethyl acetate, 5:1). Yield 133 mg (87%), pale yellow solid, mp = 133–135 °C (ethyl acetate); ^1^H NMR (400 MHz, DMSO-*d*_6_) δ = 2.09 (s, 3H, CH_3_), 3.37 (dd, ^2^*J* = 18.1 Hz, ^3^*J* = 4.6 Hz, 1H, CH_2_), 3.95 (dd, ^2^*J* = 18.1 Hz, ^3^*J* = 9.5 Hz, 1H, CH_2_), 4.56 (dd, ^3^*J* = 9.5 Hz, ^3^*J* = 4.6 Hz, 1H, CH), 6.46 (d, ^3^*J* = 12.0 Hz, 1H, =CH), 6.53 (d, ^3^*J* = 12.0 Hz, 1H, =CH), 7.28–7.31 (m, 1H, H_Ar_), 7.36–7.37 (m, 4H, H_Ar_), 7.50–7.54 (m, 2H, H_Ar_), 7.62–7.66 (m, 1H, H_Ar_), 7.98–8.00 (m, 2H, H_Ar_) ppm; ^13^C NMR (100 MHz, DMSO-*d*_6_) δ = 28.9, 41.0, 52.2, 127.4, 127.9 (2C), 128.6 (2C), 128.7 (2C), 128.8 (2C), 129.9, 133.2, 136.2, 136.9, 142.0, 197.7, 198.4, 202.6 ppm; HRMS (ESI) calcd. for C_20_H_19_O_3_^+^ [M + H]^+^ 307.1329, found 307.1328.

### 3.3. Synthesis of (E)-1,3-diphenyloct-5-ene-1,4,7-trione (E)-***2a***

To a solution of a 3-(5-methylfuran-2-yl)-1,3-diphenylpropan-1-one **1a** (145 mg, 0.5 mmol) in THF/H_2_O (3:1, 2.5 mL) was added NBS (107 mg, 0.6 mmol) at 0 °C. The reaction mixture was stirred at the same temperature for 1 h. Then pyridine (81 µL, 1 mmol) was added. The reaction mixture was allowed to room temperature and stirred for 3 h. Upon completion, the reaction mixture was poured into a mixture of ethyl acetate (5 mL) and aq. solution of Na_2_S_2_O_3_ (5 mL) with vigorous stirring. The organic layer was separated, dried with anhydrous Na_2_SO_4_, and concentrated in vacuo. The product was purified by column chromatography (silica gel, eluent—petroleum ether/ethyl acetate, 5:1). Yield 118 mg (77%), pale yellow oil; ^1^H NMR (400 MHz, CDCl_3_) δ = 2.30 (s, 3H, CH_3_), 3.25 (dd, ^2^*J* = 18.0 Hz, *^3^J =* 3.7 Hz, 1H, CH_2_), 4.07 (dd, ^2^*J* = 18.0 Hz, *^3^J =* 10.0 Hz, 1H, CH_2_), 4.69 (dd, ^3^*J* = 10.0 Hz, *^3^J =* 3.7 Hz, 1H, CH), 6.92 (s, 2H, =CH), 7.27–7.32 (m, 3H, H_Ar_), 7.34–7.38 (m, 2H, H_Ar_), 7.43–7.47 (m, 2H, H_Ar_), 7.55–7.58 (m, 1H, H_Ar_), 7.95–7.97 (m, 2H, H_Ar_) ppm; ^13^C NMR (100 MHz, CDCl_3_) δ = 28.4, 42.9, 52.6, 128.2, 128.3 (2C), 128.7 (2C), 128.8 (2C), 129.6 (2C), 133.5, 136.5, 136.6, 136.8, 137.6, 197.8, 198.2, 198.4 ppm; HRMS (ESI) calcd. for C_20_H_19_O_3_^+^ [M + H]^+^ 307.1329, found 307.1328. 

### 3.4. Isomerization of (Z)-**2a** to (E)-***2a***

To a solution of (*Z*)-1,3-diphenyloct-5-ene-1,4,7-trione (*Z*)-**2a** (153 mg, 0.5 mmol) in THF/H_2_O (3:1, 2.5 mL) was added pyridine (81 µL, 1 mmol) at rt. The reaction mixture was stirred at the same temperature for 3 h (TLC control). Upon completion, the reaction mixture was extracted with CH_2_Cl_2_ (3 × 3 mL), washed with saturated aq. solution of NH_4_Cl (3 × 1 mL), brine (3 × 1 mL), and dried with anhydrous Na_2_SO_4_. The resulted solution passed through thin pad of silica gel and concentrated in vacuo. Yield 151 mg (99%). All spectral data are consistent with those described above.

### 3.5. General Procedure for the Synthesis of 3-(5-methylfuran-2-yl)-prop-2-en-1-ones ***3***

To a solution of 2-(3-oxoalk-1-enyl)furan **1** (0.5 mmol) in CH_2_Cl_2_ (3 mL) at 0 °C was added *m*-CPBA (70% *w*/*w*, 148 mg, 0.6 mmol). The reaction mixture was stirred at the same temperature for 1 h (TLC control) and then TFA (3.8 µL, 10 mol%) was added. The reaction mixture was allowed to room temperature and stirred overnight (TLC control). Upon completion, the reaction mixture was poured into saturated solution of NaHCO_3_ (5 mL), extracted with CH_2_Cl_2_ (3 × 2 mL), washed with saturated aqueous solution of NH_4_Cl (3 × 3 mL), dried with anhydrous Na_2_SO_4_, and concentrated in vacuo. The product was purified by column chromatography (silica gel, eluent—petroleum ether/ethyl acetate, 80:1). 

*3-(5-Methylfuran-2-yl)-1,3-diphenylprop-2-en-1-one (***3a***)* was isolated as mixture of isomers in an 89:11 ratio. Yield 130 mg (90%), pale orange oil; ^1^H NMR (400 MHz, CDCl_3_) δ = 2.43 (s, 3H, CH_3_), 6.07 (d, ^3^*J* = 3.3 Hz, 1H, H_Fur_), 6.10 (d, ^3^*J* = 3.3 Hz, 1H, H_Fur_), 7.29–7.30 (m, 2H, H_Ar_), 7.33–7.35 (m, 3H, H_Ar_), 7.40–7.42 (m, 3H, H_Ar_), 7.46–7.48 (m, 1H, =CH), 7.93–7.95 (m, 2H, H_Ar_) ppm; ^13^C NMR (100 MHz, CDCl_3_) δ = 13.4, 108.5, 115.9, 116.7, 127.3 (2C), 127.6, 127.7 (2C), 127.8 (2C), 128.5 (2C), 131.6, 136.3, 138.7, 143.2, 152.4, 154.7, 190.2 ppm; HRMS (ESI) calcd. for C_20_H_17_O_2_^+^ [M + H]^+^ 289.1223, found 289.1228.

*3-(5-Methylfuran-2-yl)-3-phenyl-1-(4-methylphenyl)prop-2-en-1-one* (**3b***)* was isolated as mixture of isomers in an 88:12 ratio. Yield 112 mg (74%), pale orange oil; ^1^H NMR (400 MHz, CDCl_3_) δ = 2.39 (s, 3H, CH_3_), 2.42 (s, 3H, CH_3_), 6.06 (d, ^3^*J* = 3.3 Hz, 1H, H_Fur_), 6.07 (d, ^3^*J* = 3.3 Hz, 1H, H_Fur_), 7.20 (AA’BB’-system, ^3^*J* = 8.0 Hz, 2H, H_Ar_), 7.27–7.30 (m, 2H, H_Ar_), 7.33–7.34 (m, 3H, H_Ar_), 7.40 (s, 1H, =CH), 7.85 (AA’BB’-system, ^3^*J* = 8.0 Hz, 2H, H_Ar_) ppm; ^13^C NMR (100 MHz, CDCl_3_) δ = 14.1, 21.7, 109.2, 116.7, 117.2, 128.0 (2C), 128.2, 128.7 (2C), 129.1 (2C), 129.2 (2C), 136.9, 137.1, 143.1, 143.6, 153.2, 155.2, 190.5 ppm; HRMS (ESI) calcd. for C_21_H_19_O_2_^+^ [M + H]^+^ 303.1380, found 303.1387.

*1-(4-Methoxyphenyl)-3-(5-methylfuran-2-yl)-3-phenylprop-2-en-1-one (***3c***)* was isolated as mixture of isomers in a 90:10 ratio. Yield 138 mg (87%), pale yellow oil; ^1^H NMR (400 MHz, CDCl_3_) δ = 2.42 (s, 3H, CH_3_), 3.85 (s, 3H, OCH_3_), 6.05 (d, ^3^*J* = 3.5 Hz, 1H, H_Fur_), 6.07 (d, ^3^*J* = 3.5 Hz, 1H, H_Fur_), 6.89 (AA’BB’-system, ^3^*J* = 8.8 Hz, 2H, H_Ar_), 7.28–7.30 (m, 2H, H_Ar_), 7.33–7.34 (m, 3H, H_Ar_), 7.38 (s, 1H, =CH), 7.94 (AA’BB’-system, ^3^*J* = 8.8 Hz, 2H, H_Ar_) ppm; ^13^C NMR (100 MHz, CDCl_3_) δ = 14.1, 55.5, 109.1, 113.7 (2C), 116.8, 116.9, 128.0 (2C), 128.2, 129.2 (2C), 130.9 (2C), 132.4, 137.2, 143.1, 153.2, 155.1, 163.2, 189.6 ppm; HRMS (ESI) calcd. for C_21_H_19_O_3_^+^ [M + H]^+^ 319.1329, found 319.1325.

*1-(4-Chlorophenyl)-3-(5-methylfuran-2-yl)-3-phenylprop-2-en-1-one (***3d***)* was isolated as mixture of isomers in an 89:11 ratio. Yield 124 mg (77%), pale orange oil; ^1^H NMR (400 MHz, CDCl_3_) δ = 2.43 (s, 3H, CH_3_), 6.07 (d, ^3^*J* = 3.4 Hz, 1H, H_Fur_), 6.11 (d, ^3^*J* = 3.4 Hz, 1H, H_Fur_), 7.27–7.28 (m, 1H, H_Ar_), 7.33–7.34 (m, 3H, H_Ar_), 7.35–7.37 (m, 3H, H_Ar_), 7.42–7.44 (m, 1H, =CH), 7.85–7.87 (m, 2H, H_Ar_) ppm; ^13^C NMR (100 MHz, CDCl_3_) δ = 13.4, 108.6, 115.2, 117.2, 127.4 (2C), 127.7, 128.0 (2C), 128.5 (2C), 129.2 (2C), 137.1, 138.0, 143.7, 144.7, 152.2, 154.9, 189.0 ppm; HRMS (ESI) calcd. for C_20_H_16_ClO_2_^+^ [M + H]^+^ 323.0833, found 323.0830.

*3-(5-Methylfuran-2-yl)-1-(4-nitrophenyl)-3-phenylprop-2-en-1-one (***3e***)* was isolated as mixture of isomers in an 88:12 ratio. Yield 150 mg (90%), pale orange oil; ^1^H NMR (400 MHz, CDCl_3_) δ = 2.45 (s, 3H, CH_3_), 6.11 (d, ^3^*J* = 3.4 Hz, 1H, H_Fur_), 6.18 (d, ^3^*J* = 3.4 Hz, 1H, H_Fur_), 7.27–7.28 (m, 2H, H_Ar_), 7.33–7.35 (m, 4H, H_Ar+=CH_), 8.00 (AA’BB’-system, ^3^*J* = 8.8 Hz, 2H, H_Ar_), 8.20 (AA’BB’-system, ^3^*J* = 8.8 Hz, 2H, H_Ar_) ppm; ^13^C NMR (100 MHz, CDCl_3_) δ = 13.5, 109.0, 114.7, 118.5, 122.9 (2C), 127.5 (2C), 128.1, 128.5 (2C), 128.7 (2C), 135.7, 143.7, 145.1, 149.0, 151.9, 155.7, 188.7 ppm; HRMS (ESI) calcd. for C_20_H_16_NO_4_^+^ [M + H]^+^ 334.1074, found 334.1075.

*3-(5-Methylfuran-2-yl)-1-(naphthalen-2-yl)-3-phenylprop-2-en-1-one (***3f***)* was isolated as mixture of isomers in an 89:11 ratio. Yield 159 mg (94%), pale yellow oil; ^1^H NMR (400 MHz, CDCl_3_) δ = 2.46 (s, 3H, CH_3_), 6.09 (d, ^3^*J* = 3.3 Hz, 1H, H_Fur_), 6.13 (d, ^3^*J* = 3.3 Hz, 1H, H_Fur_), 7.33 (br s, 5H, H_Ar+=CH_), 7.52–7.57 (m, 3H, H_Ar_), 7.84–7.86 (m, 2H, H_Ar_), 7.94–7.96 (m, 1H, H_Ar_), 7.98–8.01 (m, 1H, H_Ar_), 8.49 (br s, 1H, H_Ar_) ppm; ^13^C NMR (100 MHz, CDCl_3_) δ = 13.4, 108.5, 116.1, 116.8, 123.9, 125.9, 127.2, 127.3 (2C), 127.4, 127.5, 127.6, 128.5 (2C), 128.9, 129.3, 132.0, 134.7, 136.1, 136.3, 143.2, 152.5, 154.7, 190.1 ppm; HRMS (ESI) calcd. for C_24_H_19_O_2_^+^ [M + H]^+^ 339.1380, found 339.1383.

*3-(5-Methylfuran-2-yl)-3-phenyl-1-(thiophen-2-yl)prop-2-en-1-one (***3g***)* was isolated as mixture of isomers in a 94:6 ratio. Yield 123 mg (84%), pale yellow oil; ^1^H NMR (400 MHz, DMSO-*d*_6_) δ = 2.41 (s, 3H, CH_3_), 6.13 (d, ^3^*J* = 3.3 Hz, 1H, H_Fur_), 6.26 (d, ^3^*J* = 3.3 Hz, 1H, H_Fur_), 7.21–7.25 (m, 3H, H_Ar+Th_), 7.31 (s, 1H, =CH), 7.35–7.40 (m, 3H, H_Ar+Th_), 7.92–7.94 (m, 2H, H_Ar_) ppm; ^13^C NMR (100 MHz, DMSO-*d*_6_) δ = 13.6, 109.5, 114.9, 117.5, 127.6 (2C), 127.9, 128.6, 128.7 (2C), 132.0, 134.3, 136.2, 143.0, 146.2, 151.9, 155.5, 181.2 ppm; HRMS (ESI) calcd. for C_18_H_15_SO_2_^+^ [M + H]^+^ 295.0787, found 295.0792.

*3-(5-Methylfuran-2-yl)-1-phenyl-3-(4-methylphenyl)prop-2-en-1-one (***3h***)* was isolated as mixture of isomers in an 89:11 ratio. Yield 115 mg (76%), pale yellow oil; ^1^H NMR (400 MHz, CDCl_3_) δ = 2.37 (s, 3H, CH_3_), 2.42 (s, 3H, CH_3_), 6.07 (d, ^3^*J* = 3.2 Hz, 1H, H_Fur_), 6.13 (d, ^3^*J* = 3.2 Hz, 1H, H_Fur_), 7.15 (AA’BB’-system, ^3^*J* = 7.8 Hz, 2H, H_Ar_), 7.18–7.20 (m, 2H, H_Ar_), 7.40 (s, 1H, =CH), 7.41–7.43 (m, 2H, H_Ar_), 7.47–7.51 (m, 1H, H_Ar_), 7.96 (AA’BB’-system, ^3^*J* = 7.8 Hz, 2H, H_Ar_) ppm; ^13^C NMR (100 MHz, CDCl_3_) δ = 14.1, 21.5, 109.2, 116.2, 117.5, 128.4 (2C), 128.6 (2C), 128.8 (2C), 129.1 (2C), 132.3, 133.9, 138.1, 139.5, 144.2, 153.2, 155.3, 190.8 ppm; HRMS (ESI) calcd. for C_21_H_19_O_2_^+^ [M + H]^+^ 303.1380, found 303.1370.

*3-(4-Methoxyphenyl)-3-(5-methylfuran-2-yl)-1-phenylprop-2-en-1-one (***3i***)* was isolated as mixture of isomers in an 89:11 ratio. Yield 130 mg (82%), pale orange oil; ^1^H NMR (400 MHz, CDCl_3_) δ = 2.42 (s, 3H, CH_3_), 3.81 (s, 3H, OCH_3_), 6.08 (d, ^3^*J* = 3.2 Hz, 1H, H_Fur_), 6.15 (d, ^3^*J* = 3.2 Hz, 1H, H_Fur_), 6.87 (AA’BB’-system, ^3^*J* = 8.7 Hz, 2H, H_Ar_), 7.24 (AA’BB’-system, ^3^*J* = 8.7 Hz, 2H, H_Ar_), 7.36 (s, 1H, =CH), 7.40–7.42 (m, 2H, H_Ar_), 7.46–7.50 (m, 1H, H_Ar_), 7.93–7.95 (m, 2H, H_Ar_) ppm; ^13^C NMR (100 MHz, CDCl_3_) δ = 14.1, 55.3, 109.2, 113.5 (2C), 116.4, 117.4, 128.4 (2C), 128.5 (2C), 128.94, 130.7 (2C), 132.3, 139.5, 143.8, 153.3, 155.3, 159.8, 191.1 ppm; HRMS (ESI) calcd. for C_21_H_19_O_3_^+^ [M + H]^+^ 319.1329, found 319.1340.

*3-(4-Bromophenyl)-3-(5-methylfuran-2-yl)-1-phenylprop-2-en-1-one (***3j***)* was isolated as mixture of isomers in a 91:9 ratio. Yield 155 mg (85%), pale orange oil; ^1^H NMR (400 MHz, CDCl_3_) δ = 2.42 (s, 3H, CH_3_), 6.07–6.09 (m, 2H, H_Fur_), 7.17 (AA’BB’-system, ^3^*J* = 8.3 Hz, 2H, H_Ar_), 7.41–7.45 (m, 3H, H_Ar+=CH_), 7.47–7.51 (m, 3H, H_Ar_), 7.95 (AA’BB’-system, ^3^*J* = 8.3 Hz, 2H, H_Ar_) ppm; ^13^C NMR (100 MHz, CDCl_3_) δ = 14.1, 109.4, 116.5, 117.6, 122.5, 128.5 (2C), 128.6 (2C), 130.9 (2C), 131.3 (2C), 132.6, 136.0, 139.3, 142.9, 152.6, 155.7, 190.4 ppm; HRMS (ESI) calcd. for C_20_H_16_BrO_2_^+^ [M + H]^+^ 367.0328, found 367.0328.

*3-(5-Methylfuran-2-yl)-3-(4-nitrophenyl)-1-phenylprop-2-en-1-one (***3k***)* was isolated as mixture of isomers in a 93:7 ratio. Yield 130 mg (78%), pale orange oil; ^1^H NMR (400 MHz, CDCl_3_) δ = 2.44 (s, 3H, CH_3_), 6.02 (d, ^3^*J* = 3.3 Hz, 1H, H_Fur_), 6.10 (d, ^3^*J* = 3.3 Hz, 1H, H_Fur_), 7.45–7.48 (m, 4H, H_Ar+=CH_), 7.53–7.54 (m, 2H, H_Ar_), 7.95–7.97 (m, 2H, H_Ar_), 8.23–8.25 (m, 2H, H_Ar_) ppm; ^13^C NMR (100 MHz, CDCl_3_) δ = 14.1, 109.6, 116.4, 118.0, 123.5 (2C), 128.5 (2C), 128.7 (2C), 130.1 (2C), 133.0, 139.0, 142.2, 144.4, 147.8, 151.9, 156.3, 189.7 ppm; HRMS (ESI) calcd. for C_20_H_16_NO_4_^+^ [M + H]^+^ 334.1074, found 334.1073.

*3-(5-Methylfuran-2-yl)-1-phenyl-3-(thiophen-2-yl)prop-2-en-1-one (***3l***)* was isolated as mixture of isomers in a 64:36 ratio (A:B). Yield 106 mg (72%), orange oil; ^1^H NMR (400 MHz, DMSO-*d*_6_) δ = 2.01 (s, 3H, CH_3_, B), 2.40 (s, 3H, CH_3_, A), 6.09 (d, ^3^*J* = 3.3 Hz, 1H, H_Fur_, B), 6.29 (d, ^3^*J* = 3.4 Hz, 1H, H_Fur_, A), 6.48 (d, ^3^*J* = 3.4 Hz, 1H, H_Fur_, A), 6.58 (d, ^3^*J* = 3.3 Hz, 1H, H_Fur_, B), 6.79 (s, 1H, =CH, B), 7.00 (dd, ^3^*J* = 5.1 Hz, ^3^*J* = 3.6 Hz, 1H, H_Th_, A), 7.10 (dd, ^3^*J* = 3.6 Hz, *^4^J* = 1.2 Hz, 1H, H_Th_, A), 7.18 (s, 1H, =CH, A), 7.19 (dd, ^3^*J* = 5.1 Hz, ^3^*J* = 3.6 Hz, 1H, H_Th_, B), 7.42–7.44 (m, 2H, H_Ar_, B), 7.46–7.48 (m, 2H, H_Ar_, A), 7.51–7.53 (m, 1H, H_Ar_, A), 7.54–7.55 (m, 1H, H_Th_, B), 7.56–7.57 (m, 1H, H_Ar_, B), 7.58 (dd, ^3^*J* = 5.1, *^4^J =* 1.2 Hz, 1H, H_Th_, A), 7.73 (dd, ^3^*J* = 5.1, *^4^J =* 1.2 Hz, 1H, H_Th_, B), 7.79–7.82 (m, 2H, H_Ar_, B), 7.83–7.85 (m, 2H, H_Ar_, A) ppm; ^13^C NMR (100 MHz, DMSO-*d*_6_) δ = 13.5 (A), 12.9 (B), 108.1 (B), 109.2 (A), 115.4 (B), 116.3 (A), 118.9 (A), 120.9 (B), 126.8 (A), 127.8 (A), 127.9 (2C, B), 128.0 (2C, A), 128.1 (B), 128.3 (2C, B), 128.5 (2C, A), 128.6 (B), 128.7 (B), 129.5 (A), 132.3 (B), 132.6 (A), 132.9 (B), 133.8 (A), 135.9 (A), 137.9 (B), 138.0 (A), 141.1 (B), 148.1 (B), 151.0 (A), 153.6 (B), 155.1 (A), 190.9 (A), 192.2 (B) ppm; HRMS (ESI) calcd. for C_18_H_15_SO_2_^+^ [M + H]^+^ 295.0787, found 295.0794.

*(E)-3-(5-Methylfuran-2-yl)-1-phenylprop-2-en-1-one (***3m***)* [39]. Yield 80 mg (75%), pale yellow oil; ^1^H NMR (400 MHz, CDCl_3_) δ = 2.40 (s, 3H, CH_3_), 6.13 (d, ^3^*J* = 3.2 Hz, 1H, H_Fur_), 6.63 (d, ^3^*J* = 3.2 Hz, 1H, H_Fur_), 7.37 (d, ^3^*J* = 15.3 Hz, 1H, =CH), 7.46–7.51 (m, 2H, H_Ar_), 7.52–7.56 (m, 2H, H_Ar+=CH_), 8.02–8.04 (m, 2H, H_Ar_) ppm; ^13^C NMR (100 MHz, CDCl_3_) δ = 13.4, 108.7, 117.2, 117.5, 127.8 (2C), 127.9 (2C), 130.2, 131.9, 137.9, 149.9, 155.3, 189.3 ppm.

*1-(4-Methoxyphenyl)-3-(5-methylfuran-2-yl)-3-(4-nitrophenyl)prop-2-en-1-one (***3n***)* was isolated as mixture of isomers in a 95:5 ratio. Yield 140 mg (77%), yellow oil; ^1^H NMR (400 MHz, CDCl_3_) δ = 2.44 (s, 3H, CH_3_), 3.87 (s, 3H, OCH_3_), 5.99 (d, ^3^*J* = 3.3 Hz, 1H, H_Fur_), 6.08 (d, ^3^*J* = 3.3 Hz, 1H, H_Fur_), 6.93 (AA’BB’-system, ^3^*J* = 8.9 Hz, 2H, H_Ar_), 7.46 (AA’BB’-system, ^3^*J* = 8.7 Hz, 2H, H_Ar_), 7.51 (s, 1H, =CH), 7.96 (AA’BB’-system, ^3^*J* = 8.9 Hz, 2H, H_Ar_), 8.23 (AA’BB’-system, ^3^*J* = 8.7 Hz, 2H, H_Ar_) ppm; ^13^C NMR (100 MHz, CDCl_3_) δ = 13.4, 54.9, 108.7, 113.2 (2C), 116.0, 116.7, 122.7 (2C), 129.3 (2C), 130.1 (2C), 131.2, 140.7, 143.9, 147.0, 151.2, 155.3, 162.9, 187.6 ppm; HRMS (ESI) calcd. for C_21_H_18_NO_5_^+^ [M + H]^+^ 364.1179, found 364.1171.

*3-(4-Methoxyphenyl)-3-(5-methylfuran-2-yl)-1-(4-nitrophenyl)prop-2-en-1-one (***3o**) was isolated as mixture of isomers in an 88:12 ratio. Yield 165 mg (91%), pale orange oil; ^1^H NMR (400 MHz, CDCl_3_) δ = 2.43 (s, 3H, CH_3_), 3.79 (s, 3H, OCH_3_), 6.11 (d, ^3^*J* = 3.4 Hz, 1H, H_Fur_), 6.23 (d, ^3^*J* = 3.4 Hz, 1H, H_Fur_), 6.83 (AA’BB’-system, ^3^*J* = 8.7 Hz, 2H, H_Ar_), 7.20 (AA’BB’-system, ^3^*J* = 8.7 Hz, 2H, H_Ar_), 7.25 (s, 1H, =CH), 7.97 (AA’BB’-system, ^3^*J* = 8.8 Hz, 2H, H_Ar_), 8.18 (AA’BB’-system, ^3^*J* = 8.8 Hz, 2H, H_Ar_) ppm; ^13^C NMR (100 MHz, CDCl_3_) δ = 13.4, 54.7, 108.9, 113.0 (2C), 114.9, 118.1, 122.8 (2C), 127.6, 128.7 (2C), 130.3 (2C), 143.9, 144.7, 149.0, 152.1, 155.5, 159.6, 189.1 ppm; HRMS (ESI) calcd. for C_21_H_18_NO_5_^+^ [M + H]^+^ 364.1179, found 364.1178.

*1-(4-Chlorophenyl)-3-(5-methylfuran-2-yl)but-2-en-1-one (***3p***)* was isolated as mixture of isomers in a 95:5 ratio. Yield 105 mg (81%), pale yellow oil; ^1^H NMR (400 MHz, DMSO-*d*_6_) δ = 2.38 (s, 3H, CH_3_), 2.44 (s, 3H, CH_3_), 6.32 (d, ^3^*J* = 3.3 Hz, 1H, H_Fur_), 7.04 (d, ^3^*J* = 3.3 Hz, 1H, H_Fur_), 7.31 (s, 1H, =CH), 7.59 (AA’BB’-system, ^3^*J* = 8.6 Hz, 2H, H_Ar_), 7.97 (AA’BB’-system, ^3^*J* = 8.6 Hz, 2H, H_Ar_) ppm; ^13^C NMR (100 MHz, DMSO-*d*_6_) δ = 13.5, 15.1, 109.4, 113.6, 115.4, 128.7 (2C), 129.5 (2C), 137.2, 137.9, 142.6, 152.3, 155.2, 188.7 ppm; HRMS (ESI) calcd. for C_15_H_14_ClO_2_^+^ [M + H]^+^ 261.0677, found 261.0675.

## 4. Conclusions

In conclusion, we reported the formal oxidation of 3-(furan-2-yl)-1,3-di(het)arylpropan-1-ones to the corresponding prop-2-en-1-ones based on the two-step procedure that includes the oxidation of starting furans followed by unusual acid-induced cyclization of (*Z*)-2-ene-1,4,7-triketones. The scope and limitations of the developed method were studied.

## Supplementary Materials

The following are available online, Copies of NMR spectra for novel compounds are available online.
