# Supplementary material for: Transformation of 3-(Furan-2-yl)-1,3-di(het)arylpropan-1-ones to Prop-2-en-1-ones via Oxidative Furan Dearomatization/2-Ene-1,4,7-triones Cyclization
Source: Molecules. 2021 Apr 30;26(9):2637. doi: 10.3390/molecules26092637 (PMC8124928; doi:10.3390/molecules26092637)
Supplement: Supplementary file 1 [file molecules-26-02637-s001.zip › molecules-1200650.pdf]

Transformation of 3-(Furan-2-yl)-1,3-di(het)arylpropan-1-ones to the Corresponding Prop-2-en-1-ones *via* the Oxidative Furan Dearomatization/2-Ene-1,4,7-triones Cyclization

Roman O. Shcherbakov,[†] Diana A. Eshmemet'eva,[†] Anton A. Merkushev,[†] Igor V. Trushkov,[§,‡] Maxim G. Uchuskin*[†]

[†] Perm State University, Bukireva st. 15, Perm, 614990, Russian Federation, e-mail: mu@psu.ru

[§] N.D. Zelinsky Institute of Organic Chemistry Russian Academy of Sciences, Leninsky pr. 47, Moscow, 119334, Russian Federation

[‡] D. Rogachev National Medical Research Center of Pediatric Hematology, Oncology and Immunology, Samory Mashela st. 1, Moscow, 117997, Russian Federation

Supporting Information

1H, CDCl3, 400 MHz

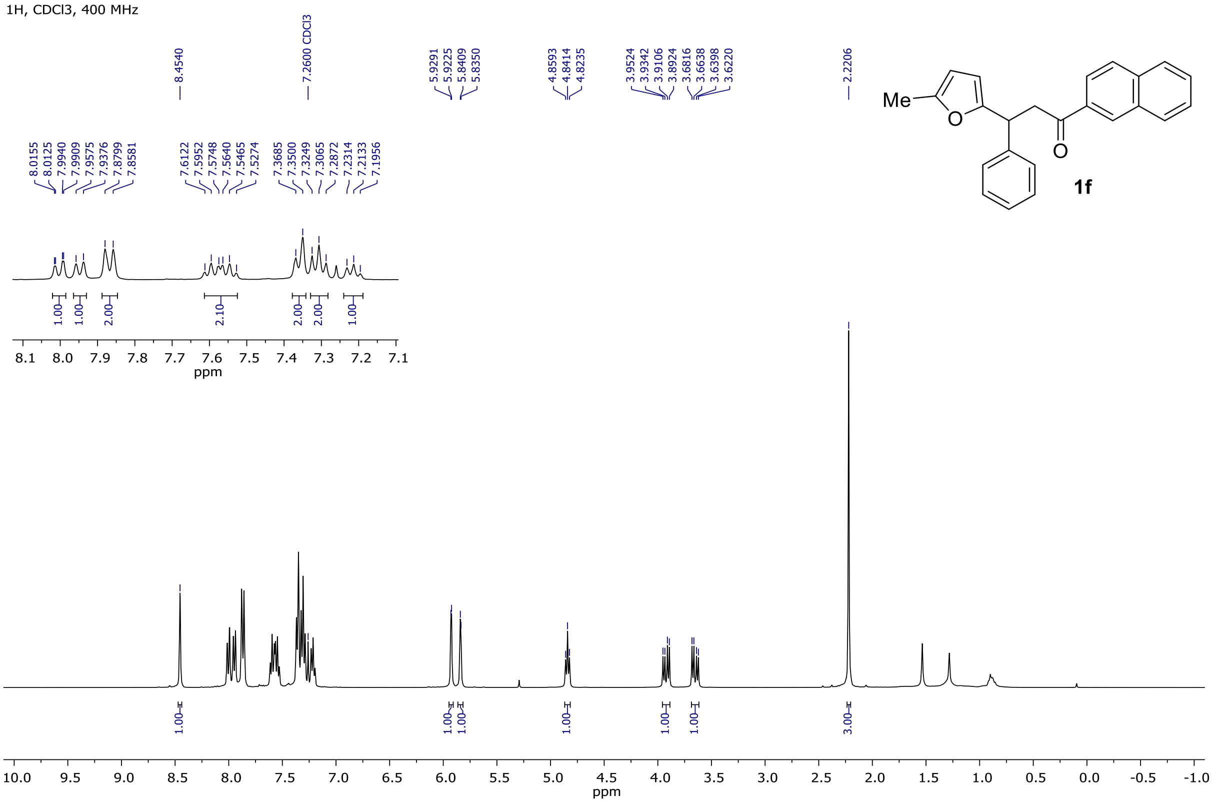

13C, CDCl3, 100 MHz

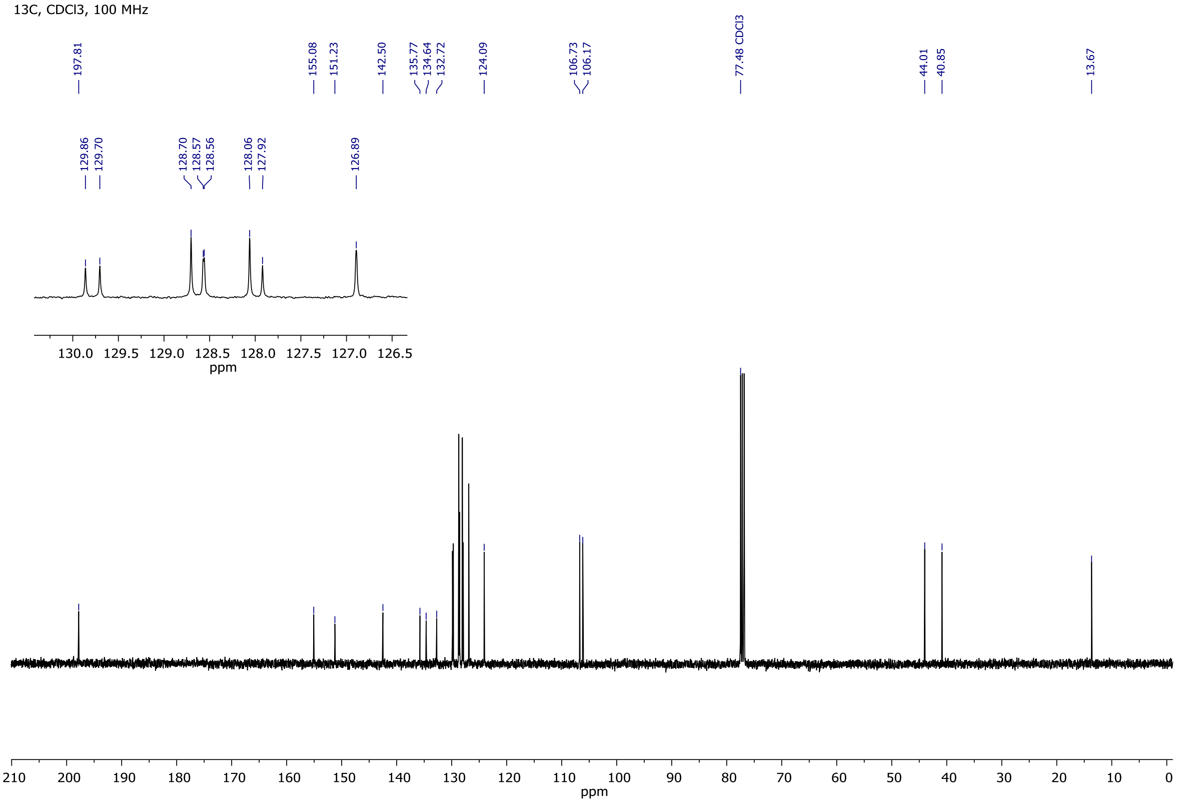

1H, CDCl3, 400 MHz

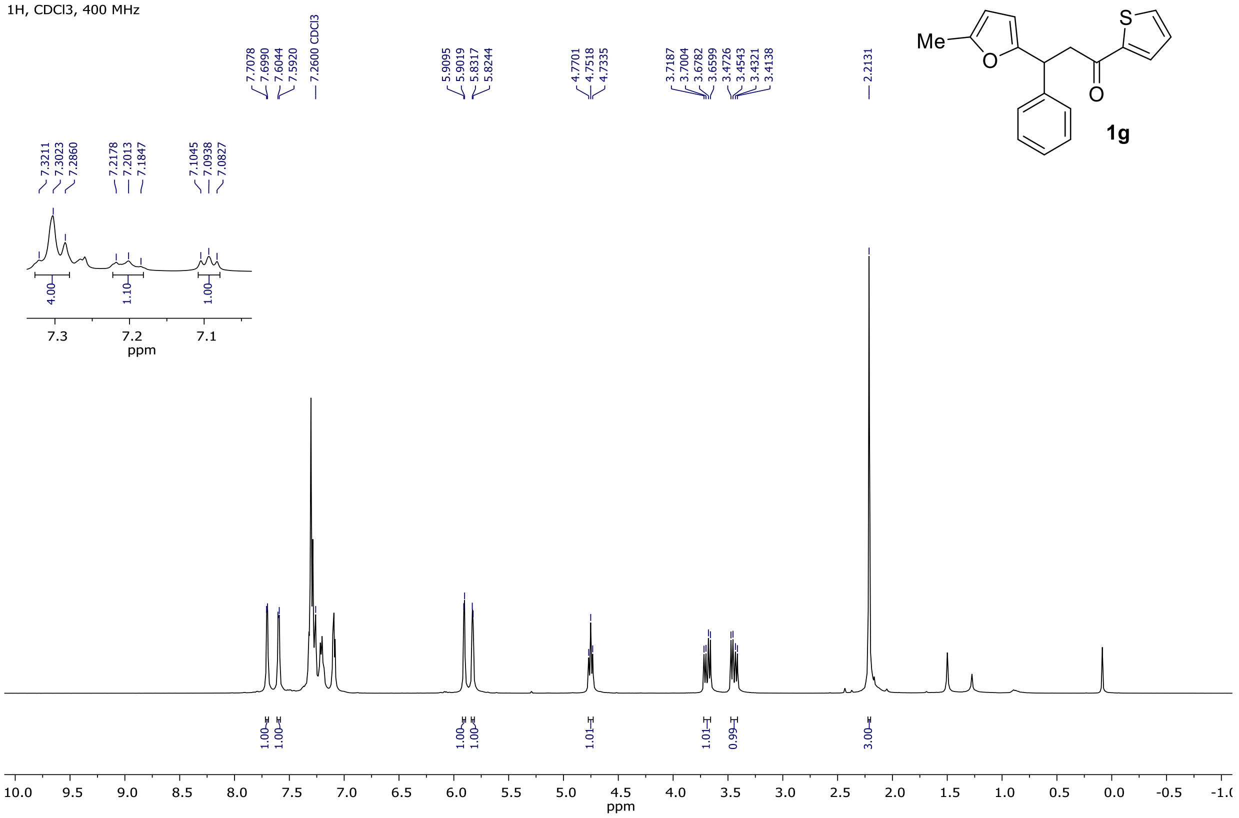

1g

13C, CDCl3, 100 MHz

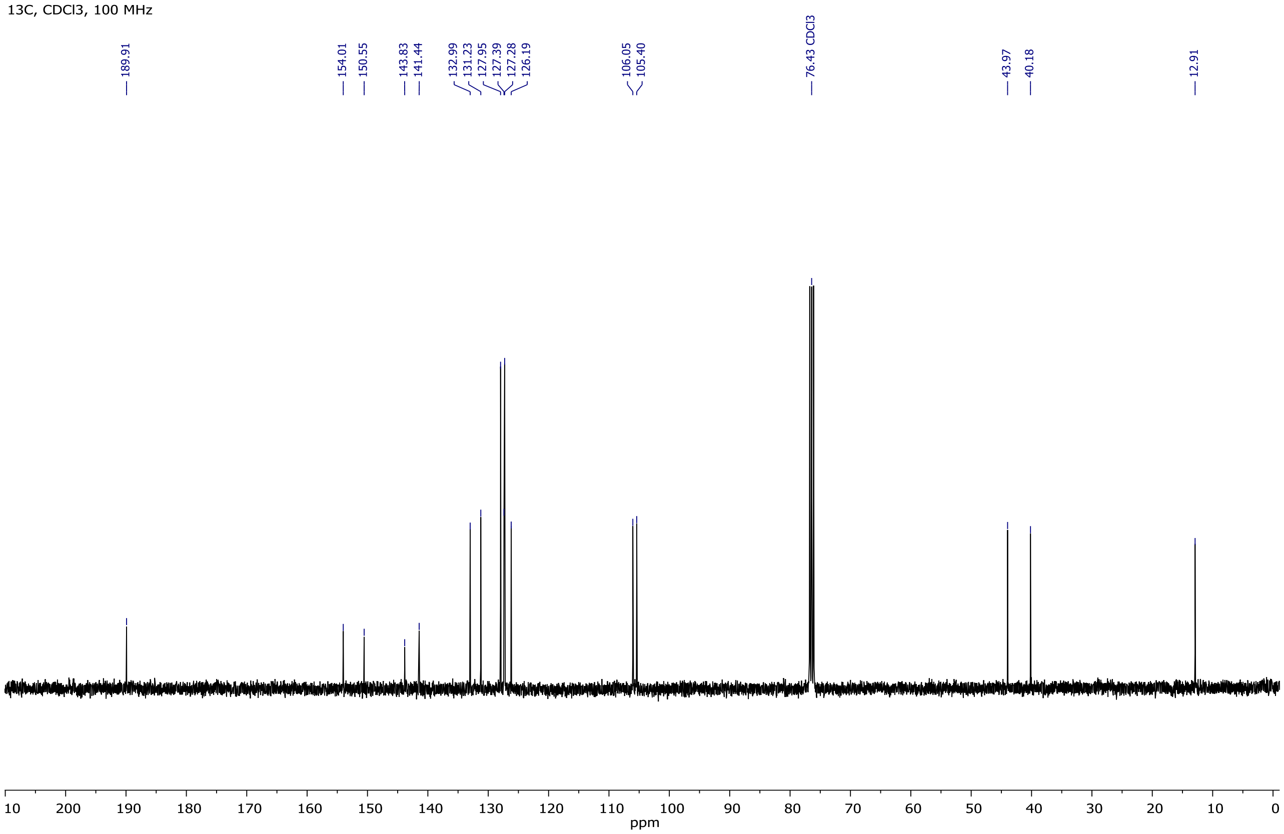

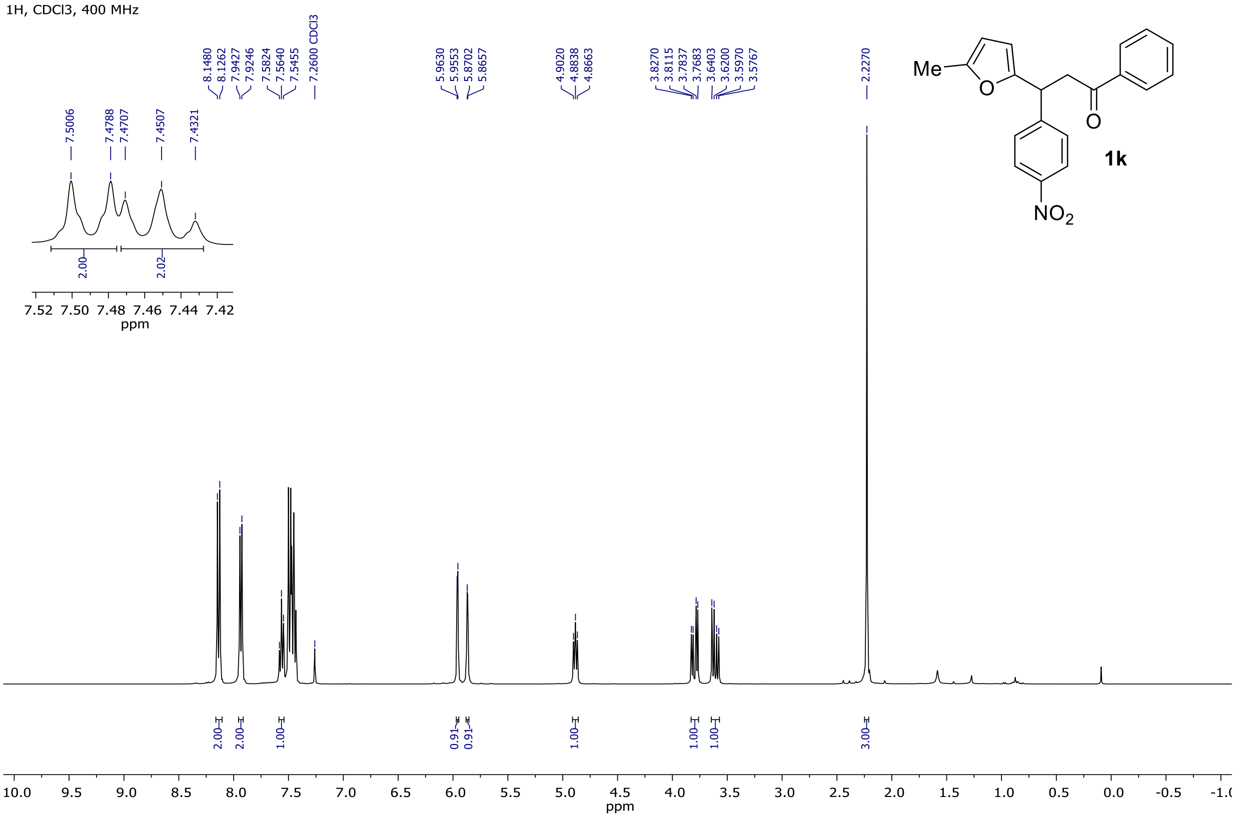
1H, CDCl3, 400 MHz
8.1480 8.1262 7.9427 7.9246 7.5824 7.5640 7.5455 7.2600 CDCl3
5.9630 5.9553 5.8702 5.8657
4.9020 4.8838 4.8663
3.8270 3.8115 3.7837 3.7683 3.6403 3.6200 3.5970 3.5767
2.2270
7.5006 7.4788 7.4707 7.4507 7.4321
2.00 2.02
7.52 7.50 7.48 7.46 7.44 7.42
ppm
2.00 2.00 1.00 0.91 0.91 1.00 1.00 1.00 3.00
10.0 9.5 9.0 8.5 8.0 7.5 7.0 6.5 6.0 5.5 5.0 4.5 4.0 3.5 3.0 2.5 2.0 1.5 1.0 0.5 0.0 -0.5
ppm
Me
O
O
NO2

1k

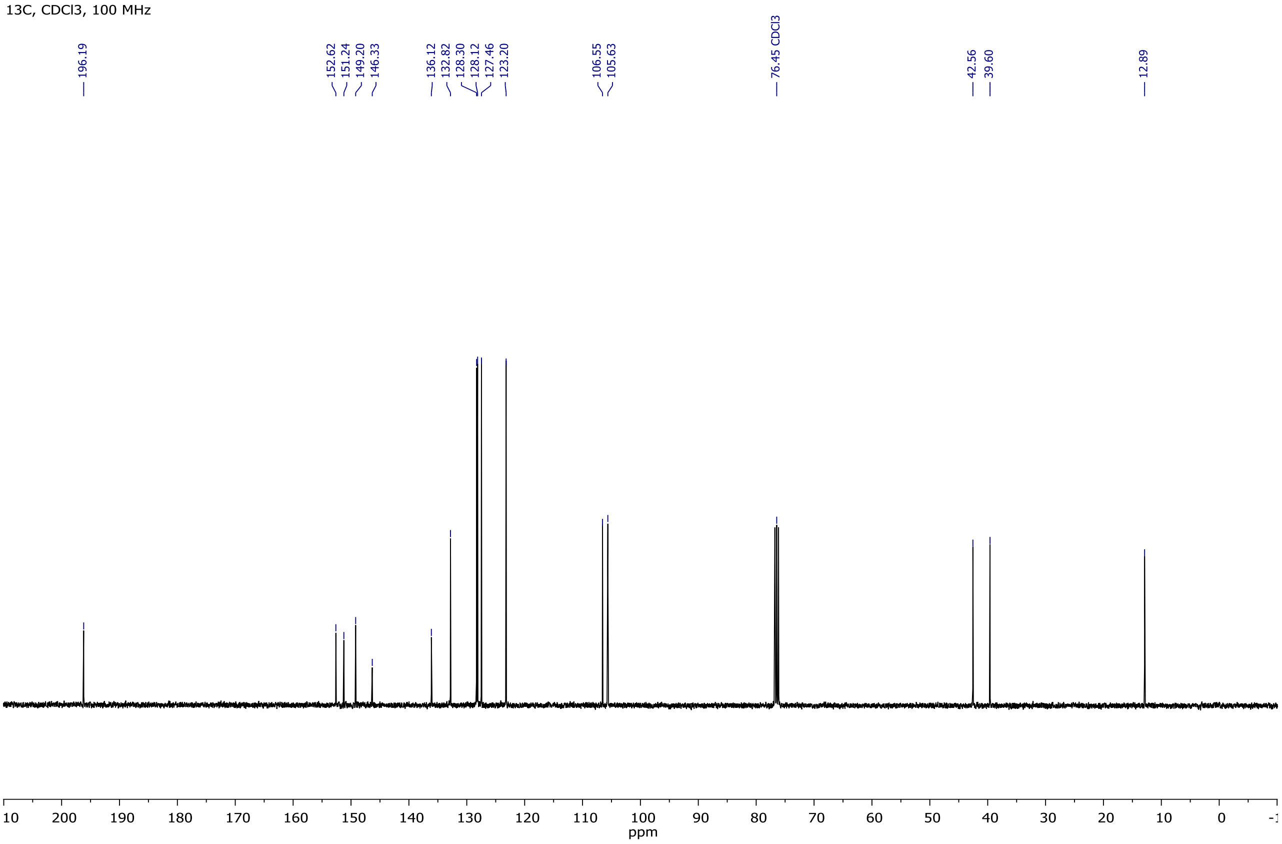
13C, CDCl3, 100 MHz
196.19
152.62 151.24 149.20 146.33
136.12 132.82 128.30 128.12 127.46 123.20
106.55 105.63
76.45 CDCl3
42.56 39.60
12.89
200 190 180 170 160 150 140 130 120 110 100 90 80 70 60 50 40 30 20 10 0
ppm

1H, CDCl3, 400 MHz

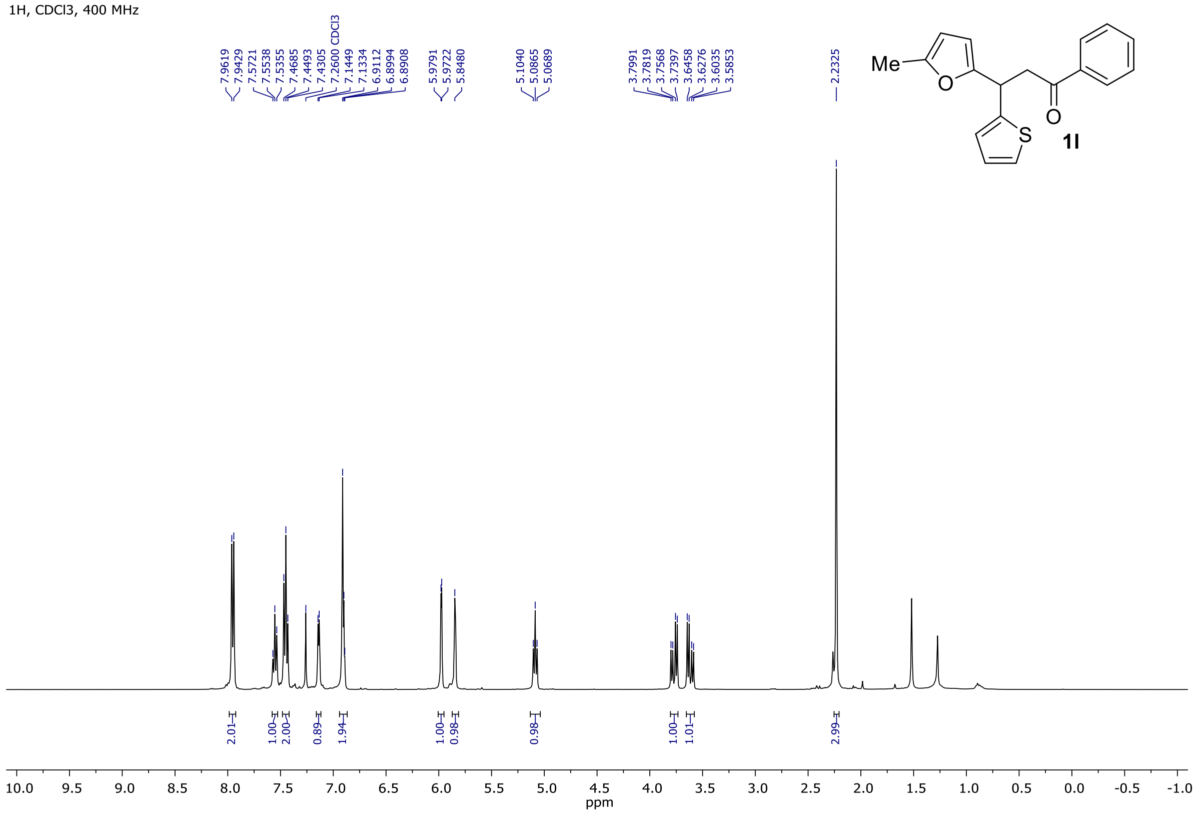

13C, CDCl3, 100 MHz

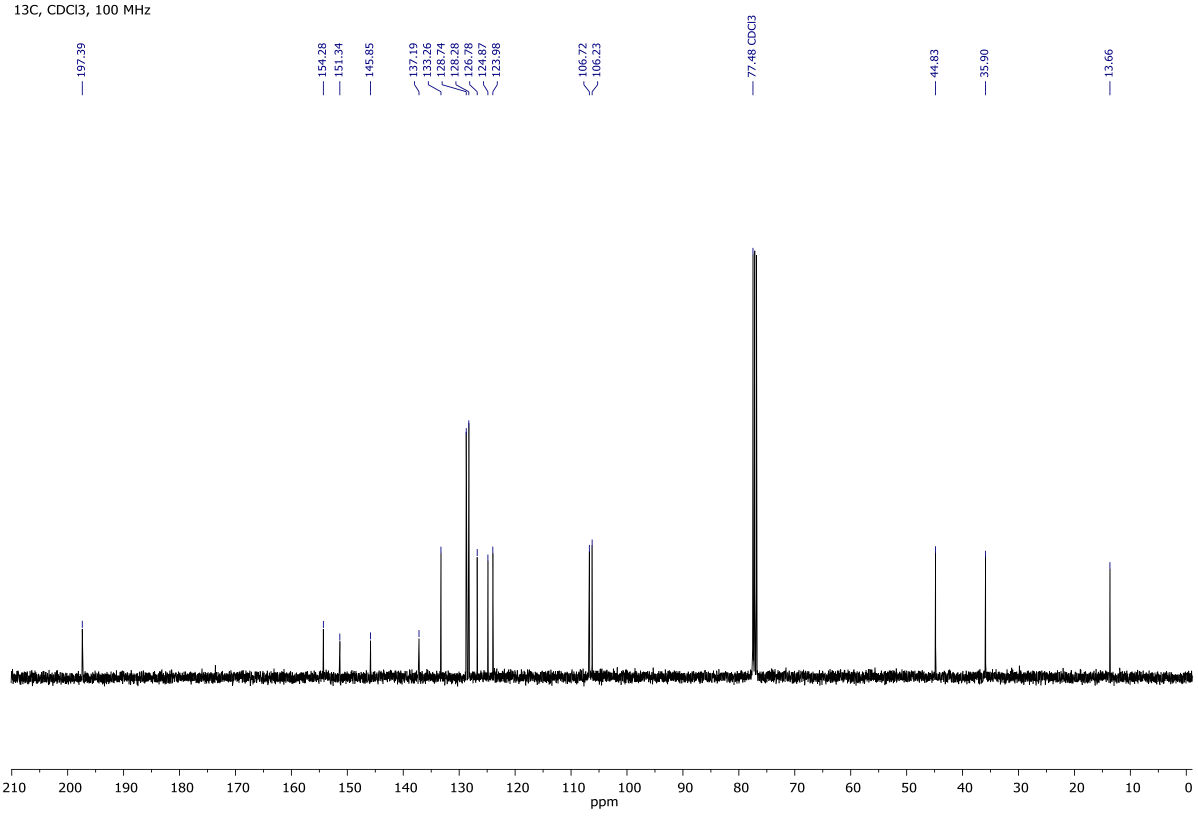

1H, CDCl3, 400 MHz

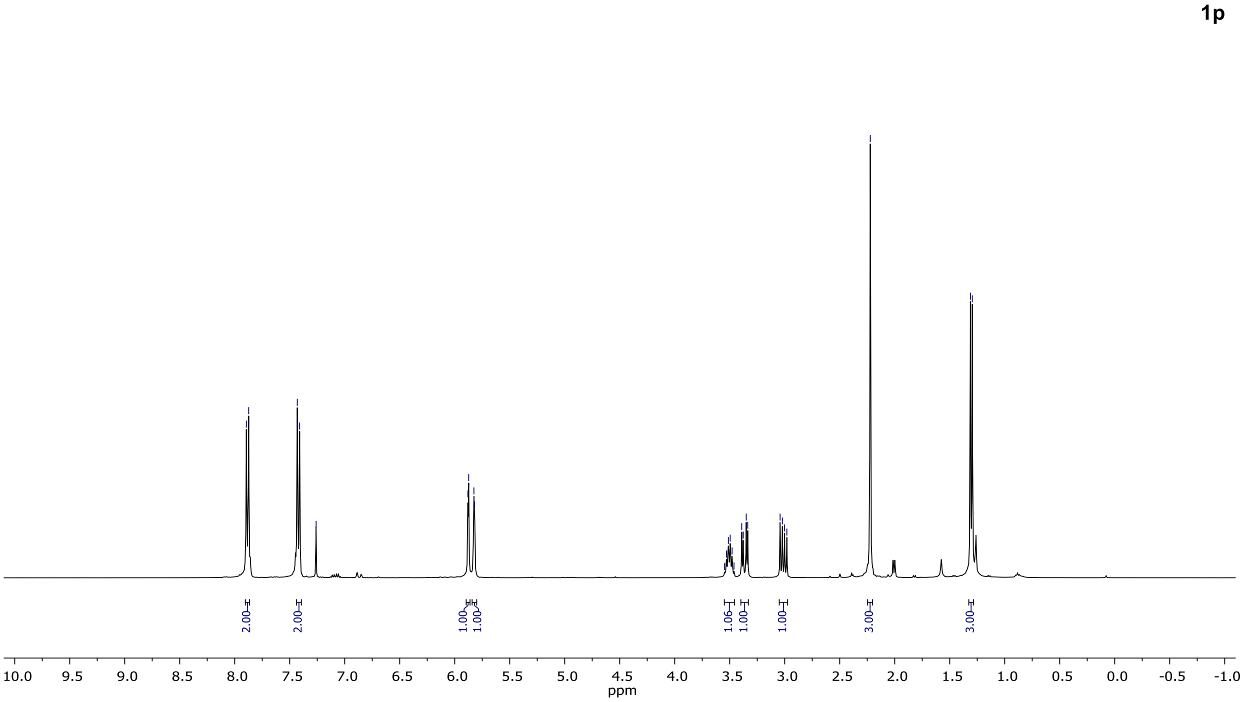

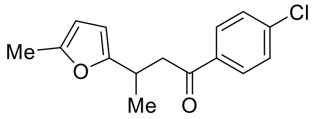

1p

13C, CDCl3, 100 MHz

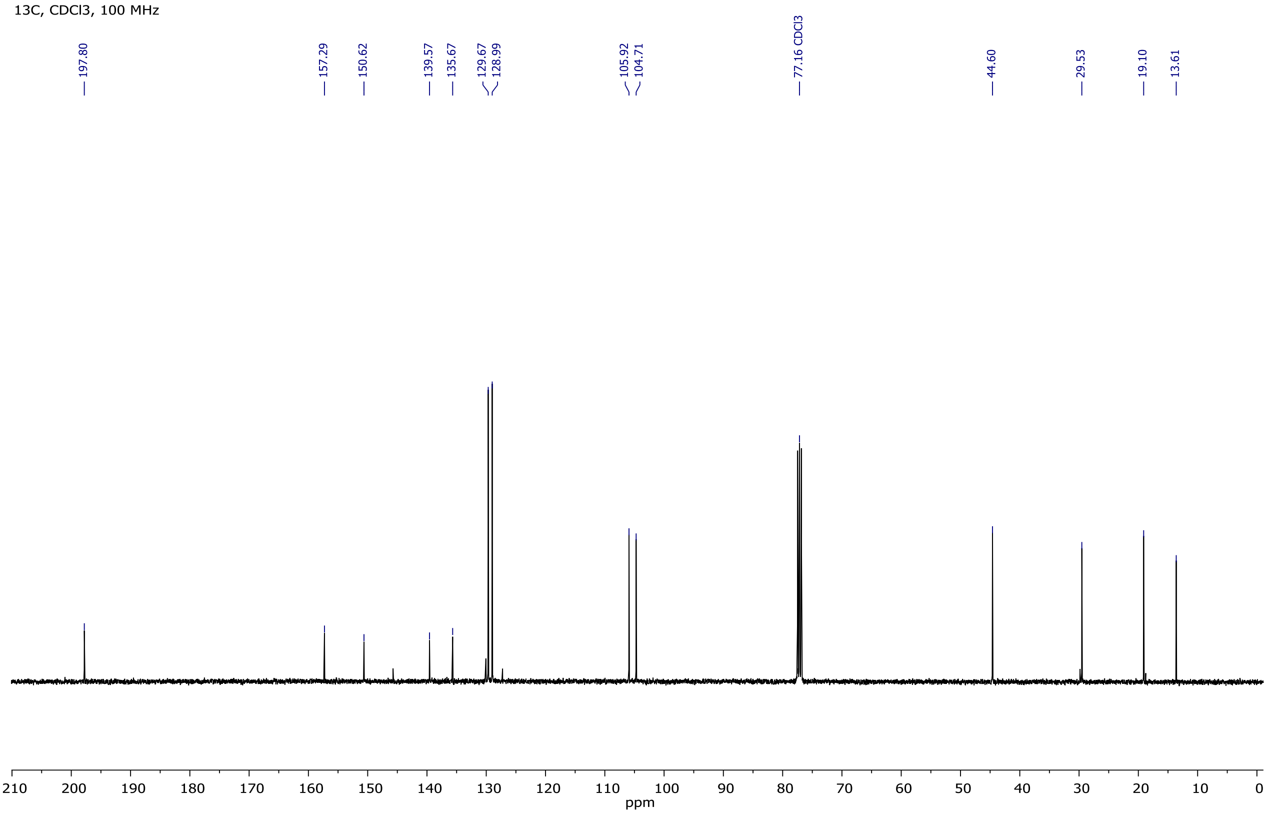

1H, DMSO-d6, 400 MHz

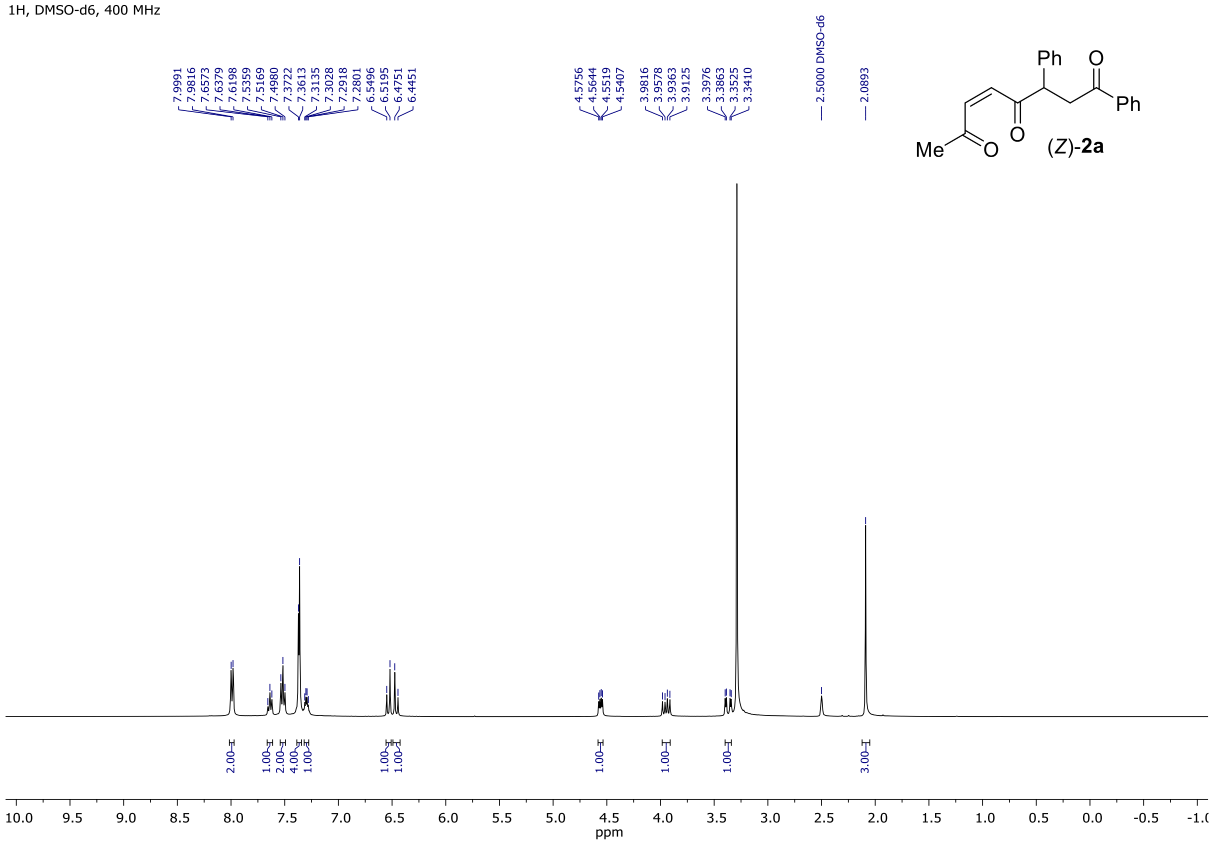

13C, DMSO-d6, 100 MHz

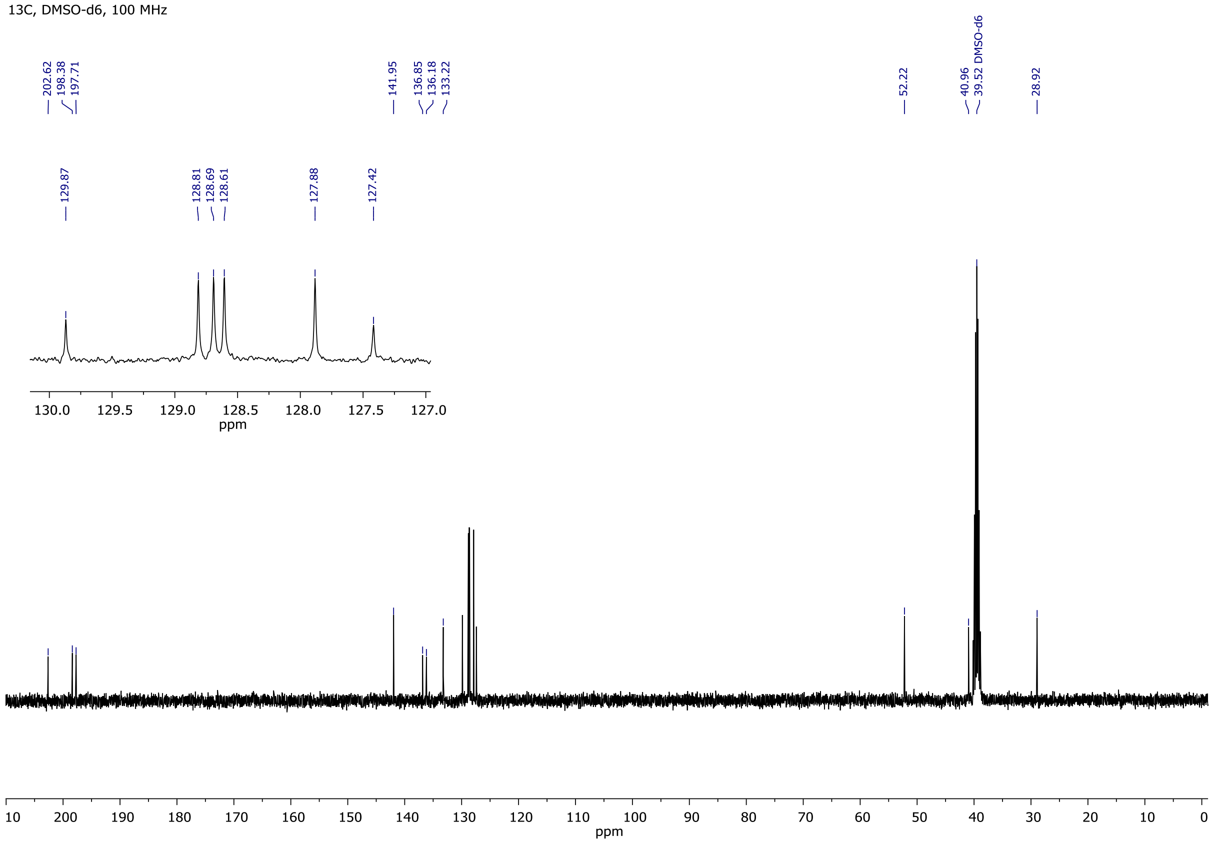

1H, CDCl3, 400 MHz

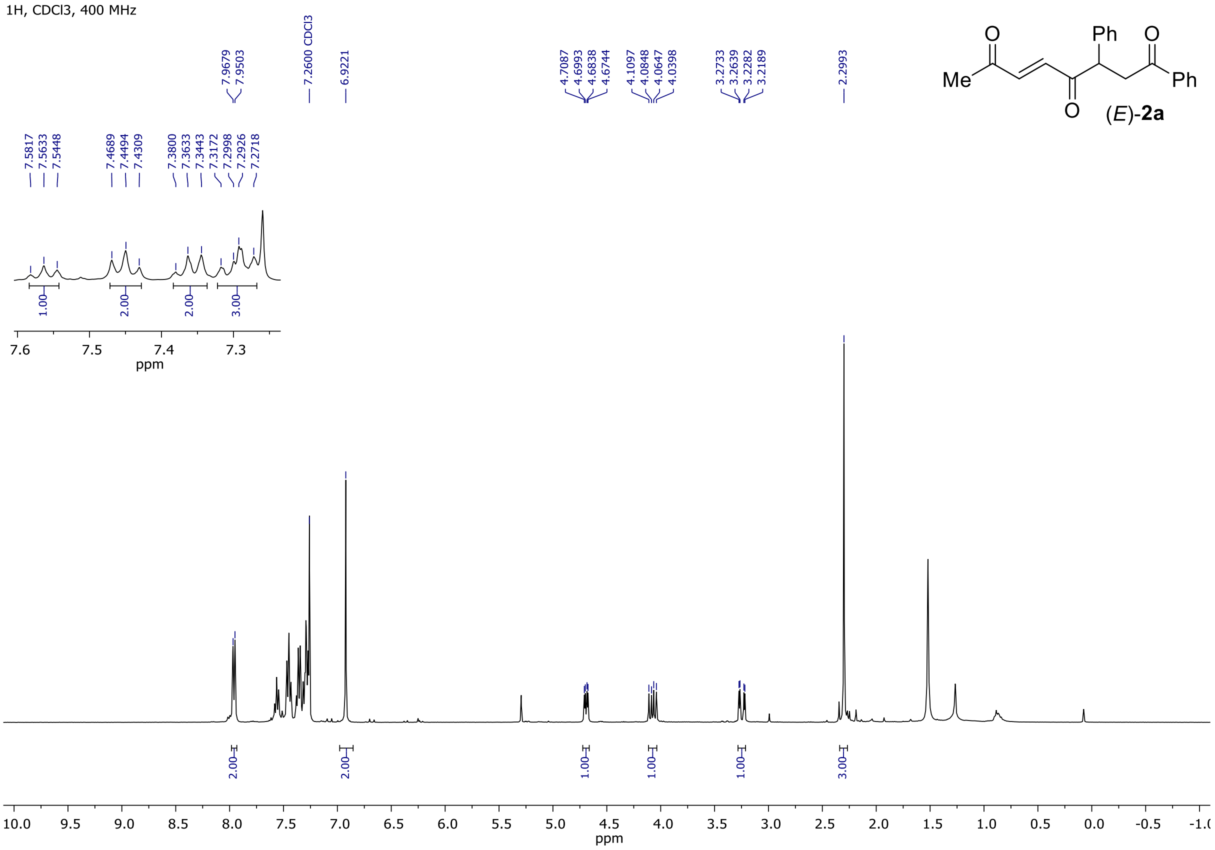

13C, CDCl3, 100 MHz

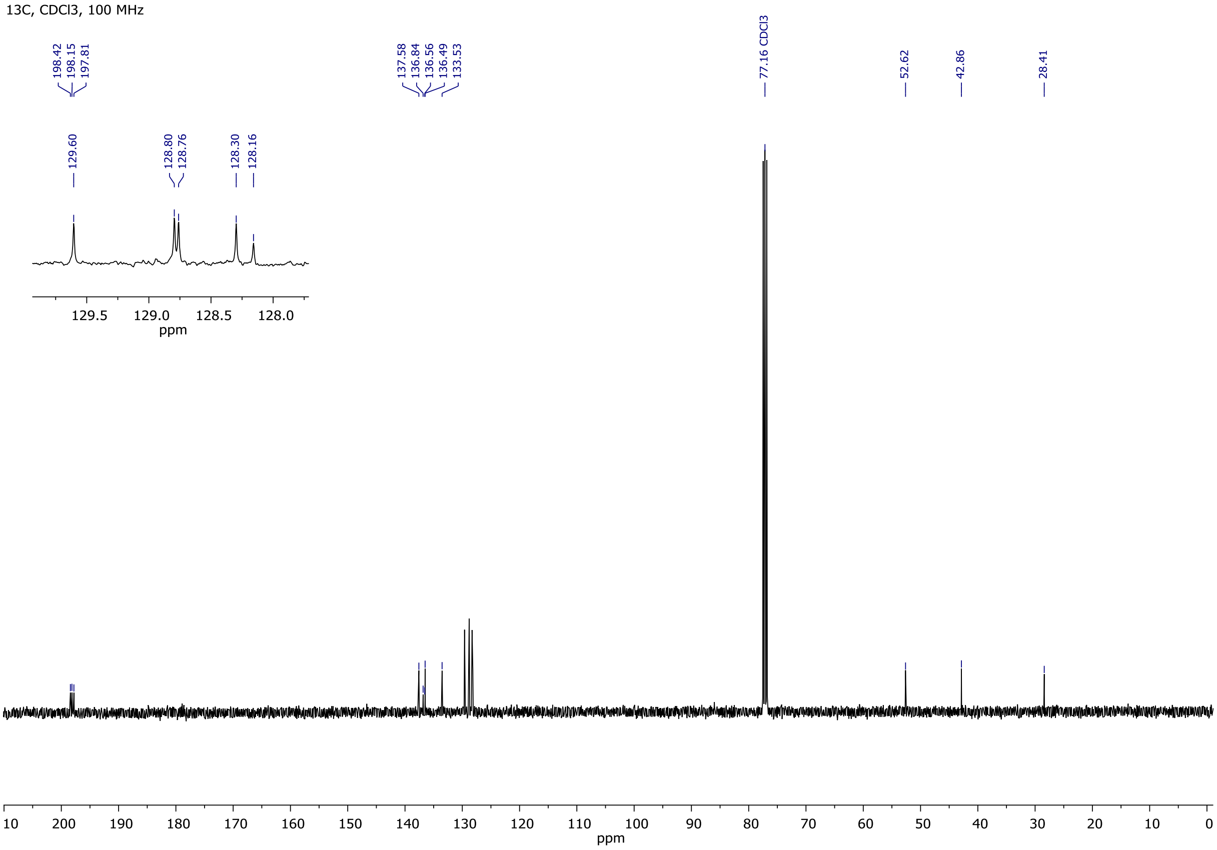

1H, CDCl3, 400 MHz

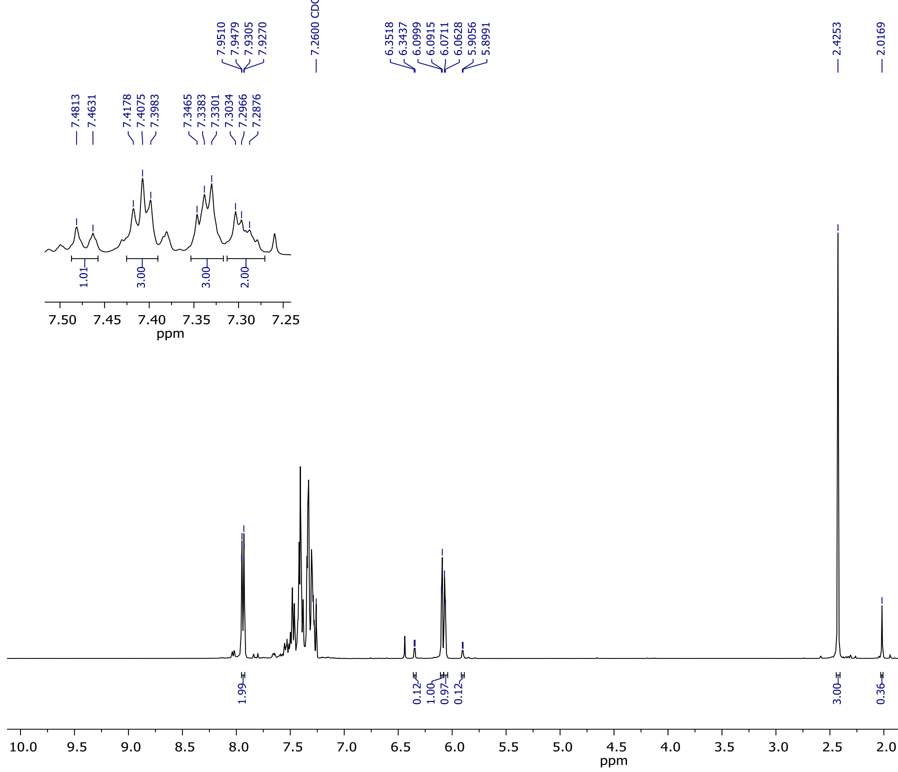

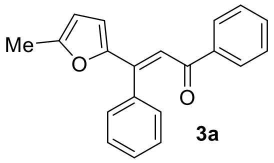

3a

13C, CDCl3, 100 MHz

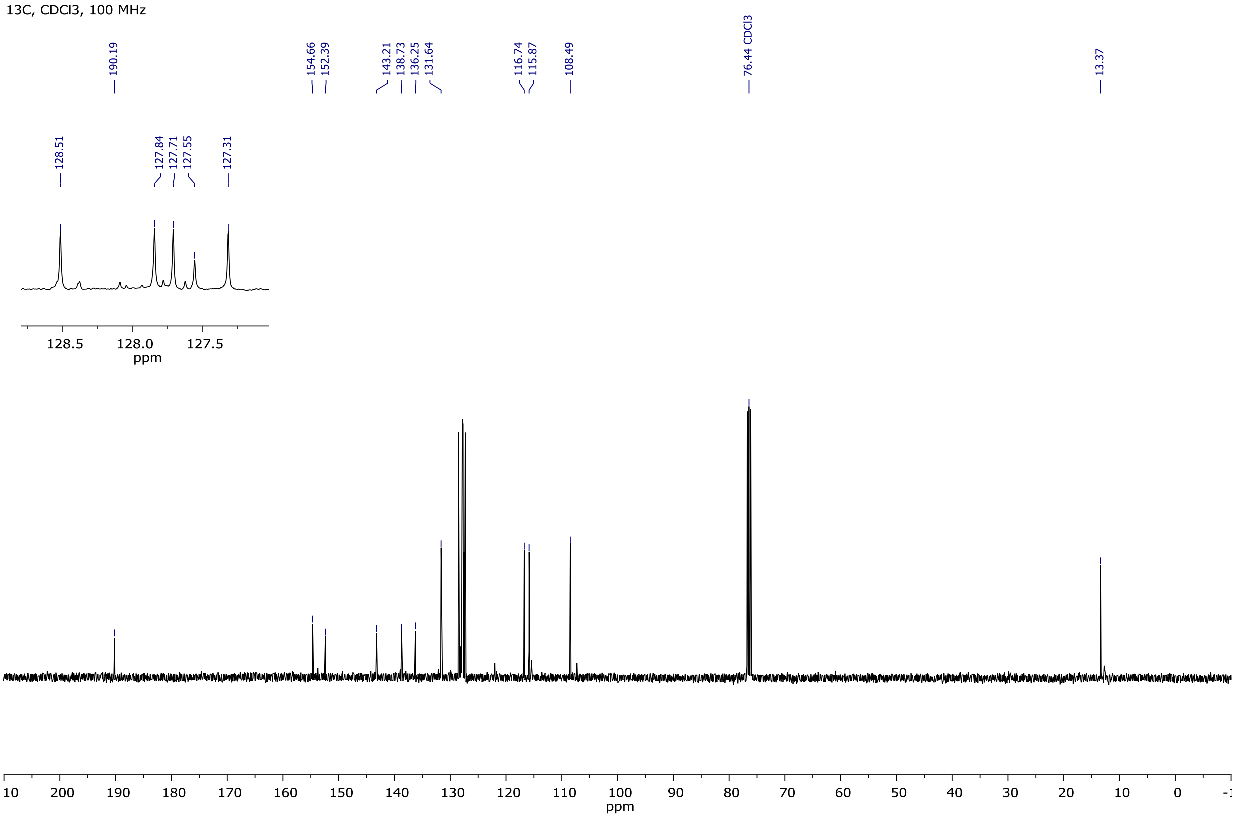

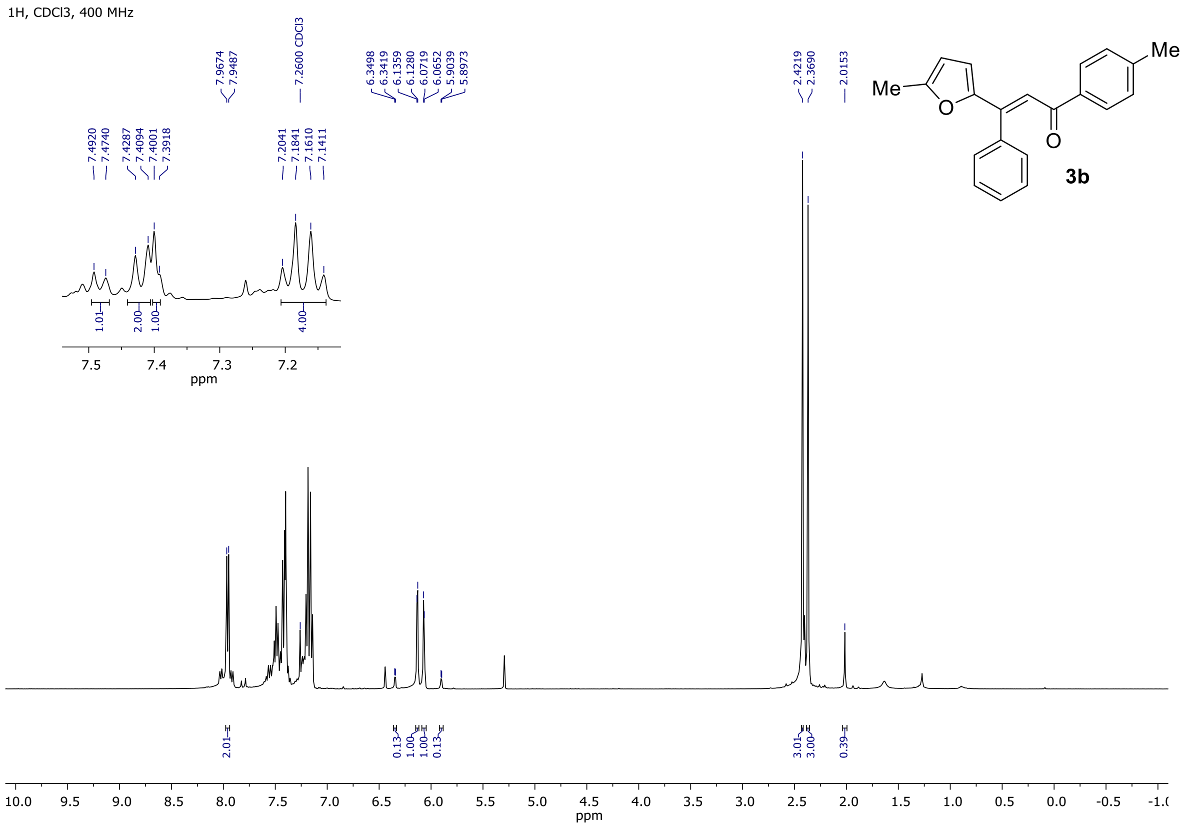
1H, CDCl3, 400 MHz
7.9674
7.9487
7.2600 CDCl3
6.3498
6.3419
6.1359
6.1280
6.0719
6.0652
5.9039
5.8973
2.4219
2.3690
2.0153
7.4920
7.4740
7.4287
7.4094
7.4001
7.3918
7.2041
7.1841
7.1610
7.1411
1.01
2.00
1.00
4.00
2.01
0.13
1.00
1.00
0.13
3.01
3.00
0.39
ppm
Me
O
Me
O
3b

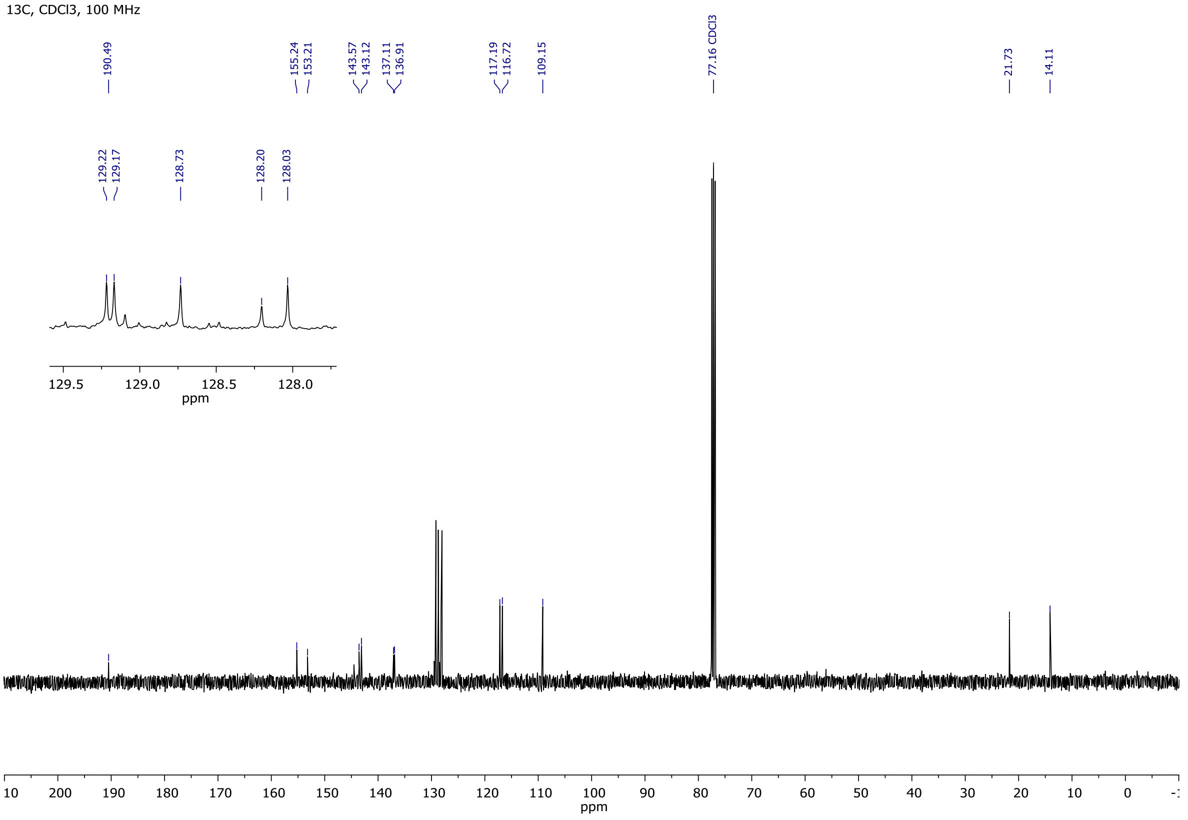
13C, CDCl3, 100 MHz
190.49
155.24
153.21
143.57
143.12
137.11
136.91
117.19
116.72
109.15
77.16 CDCl3
21.73
14.11
129.22
129.17
128.73
128.20
128.03
ppm

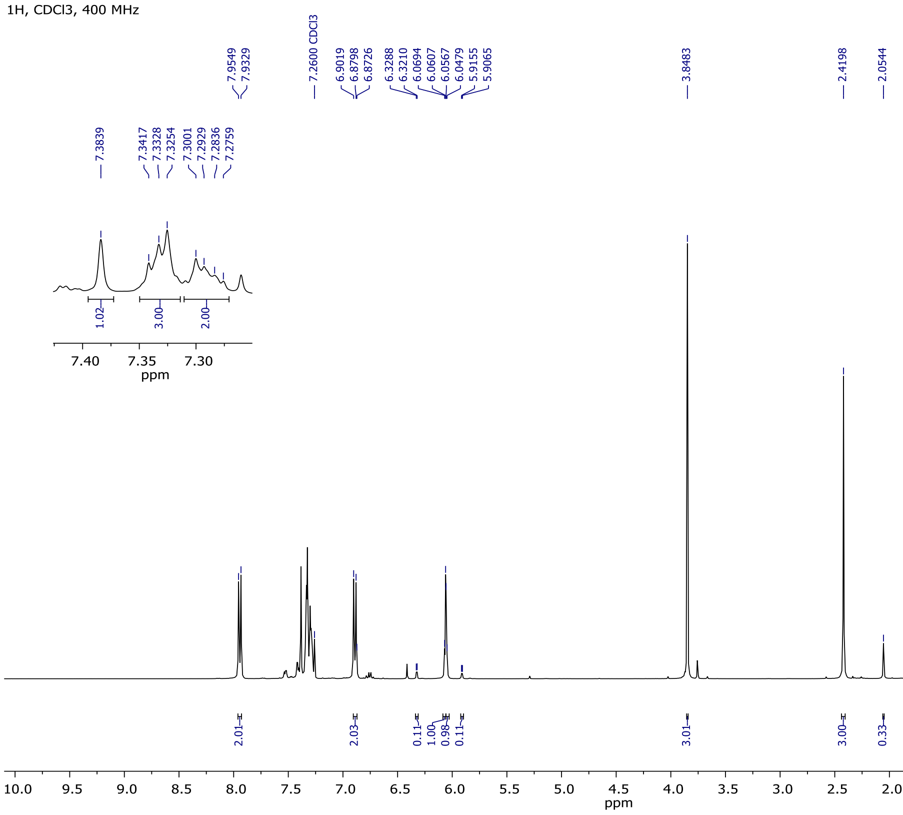
1H, CDCl3, 400 MHz
7.9549
7.9329
7.2600 CDCl3
6.9019
6.8798
6.8726
6.3288
6.3210
6.0694
6.0607
6.0567
6.0479
5.9155
5.9065
3.8483
2.4198
2.0544
7.3839
7.3417
7.3328
7.3254
7.3001
7.2929
7.2836
7.2759
1.02
3.00
2.00
ppm
2.01
2.03
0.11
1.00
0.98
0.11
3.01
3.00
0.33
ppm

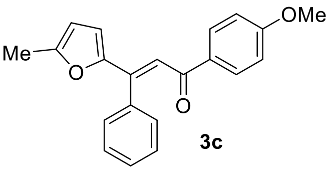
Me
O
OMe
O

3c

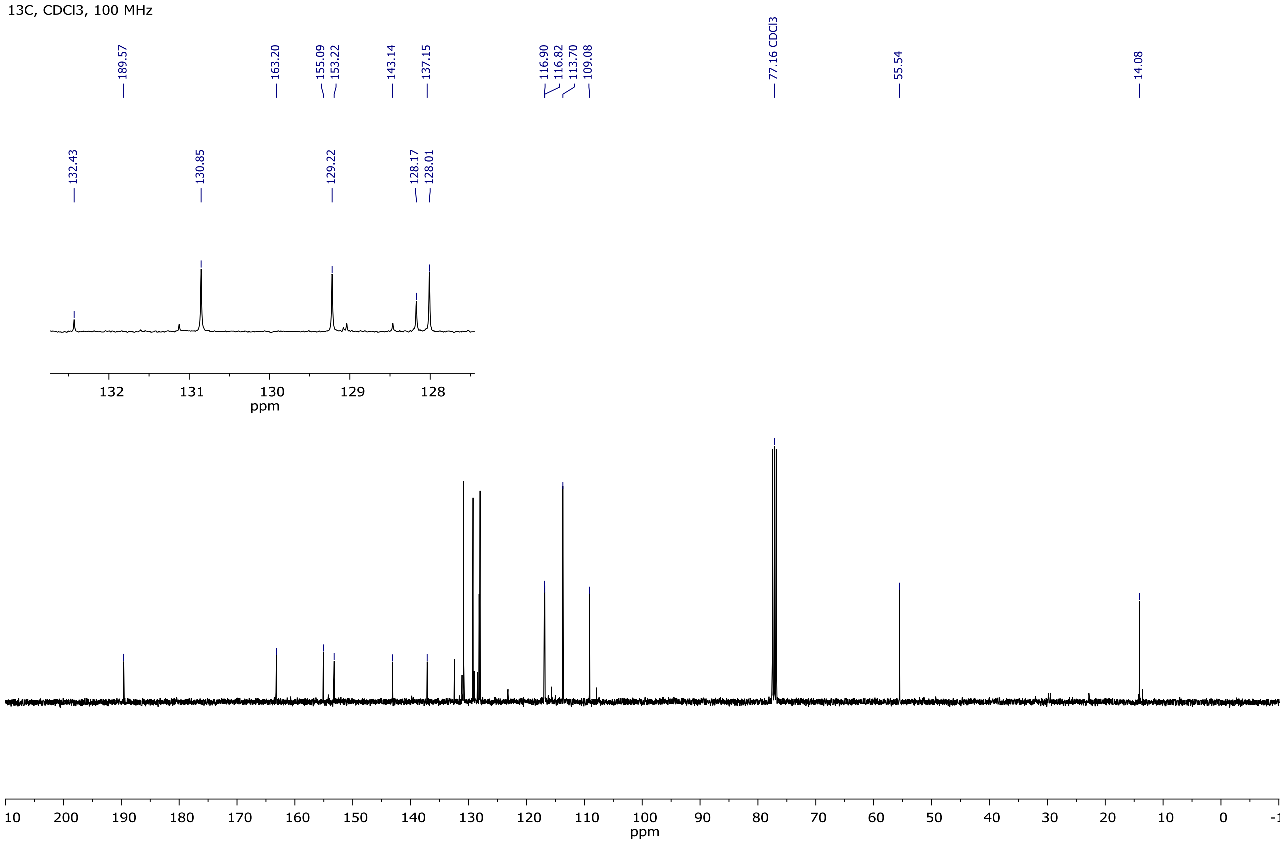
13C, CDCl3, 100 MHz
189.57
163.20
155.09
153.22
143.14
137.15
116.90
116.82
113.70
109.08
77.16 CDCl3
55.54
14.08
132.43
130.85
129.22
128.17
128.01
ppm
ppm

1H, CDCl3, 400 MHz

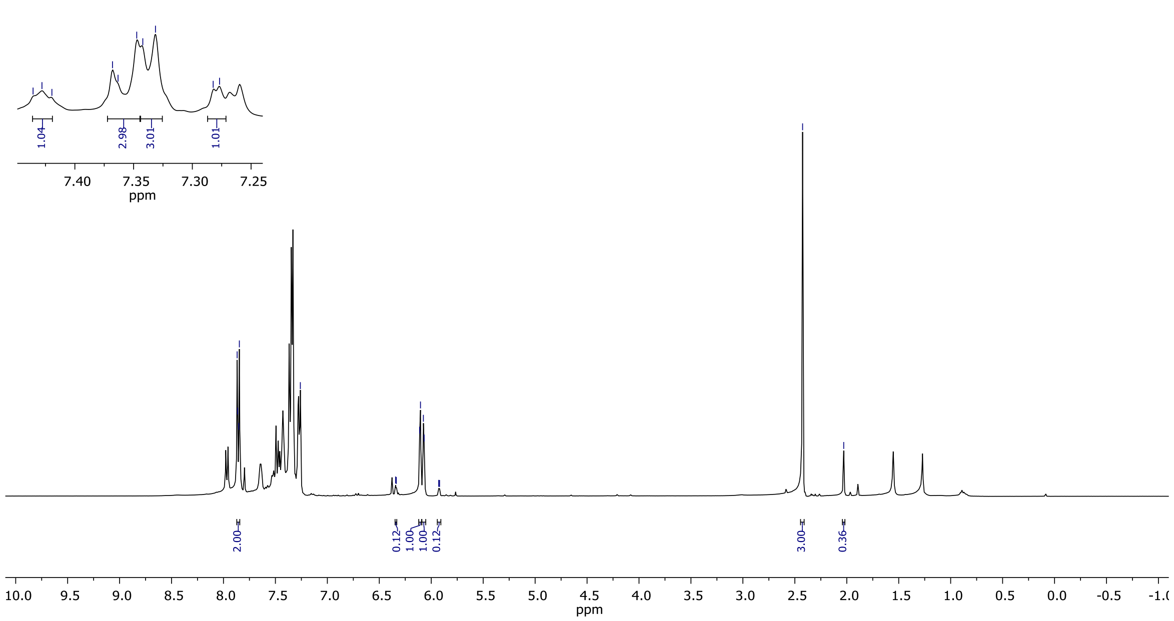

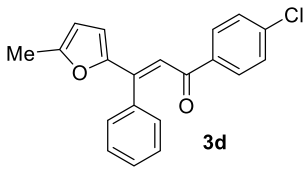

3d

13C, CDCl3, 100 MHz

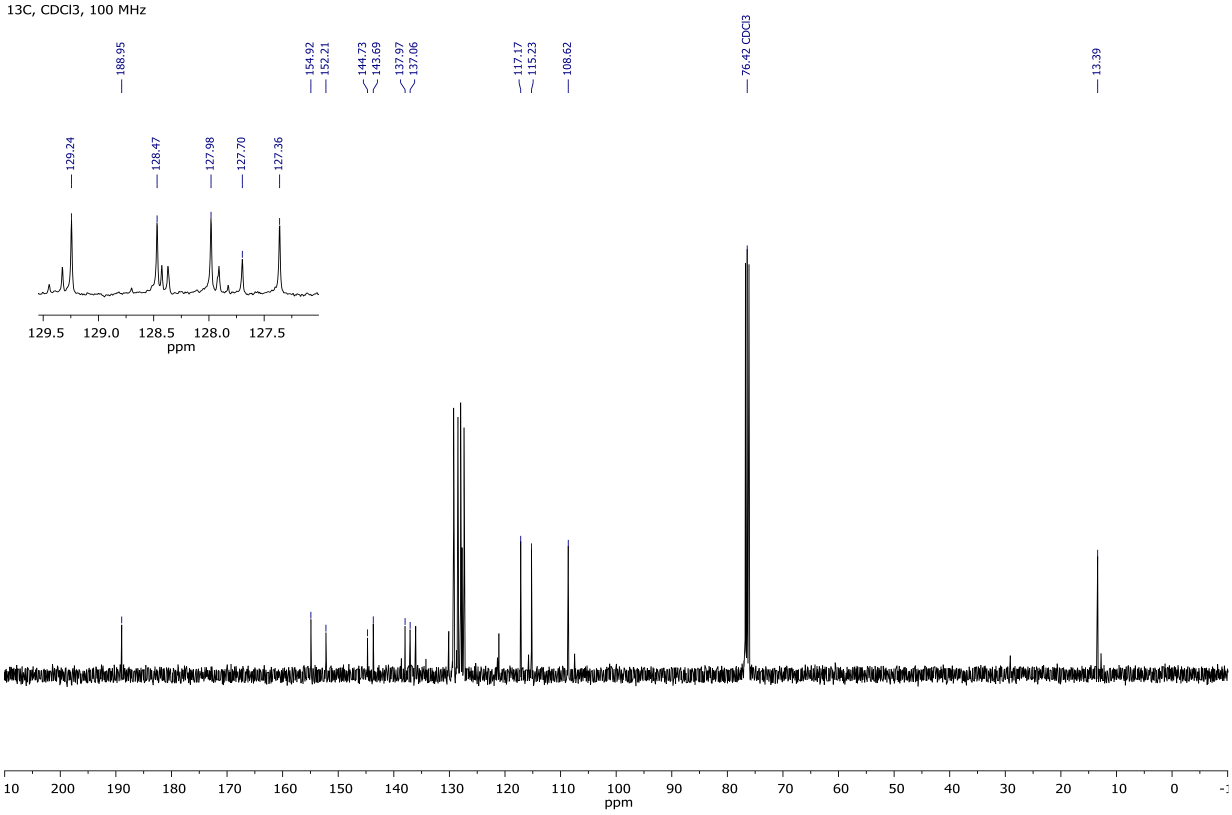

1H, CDCl3, 400 MHz

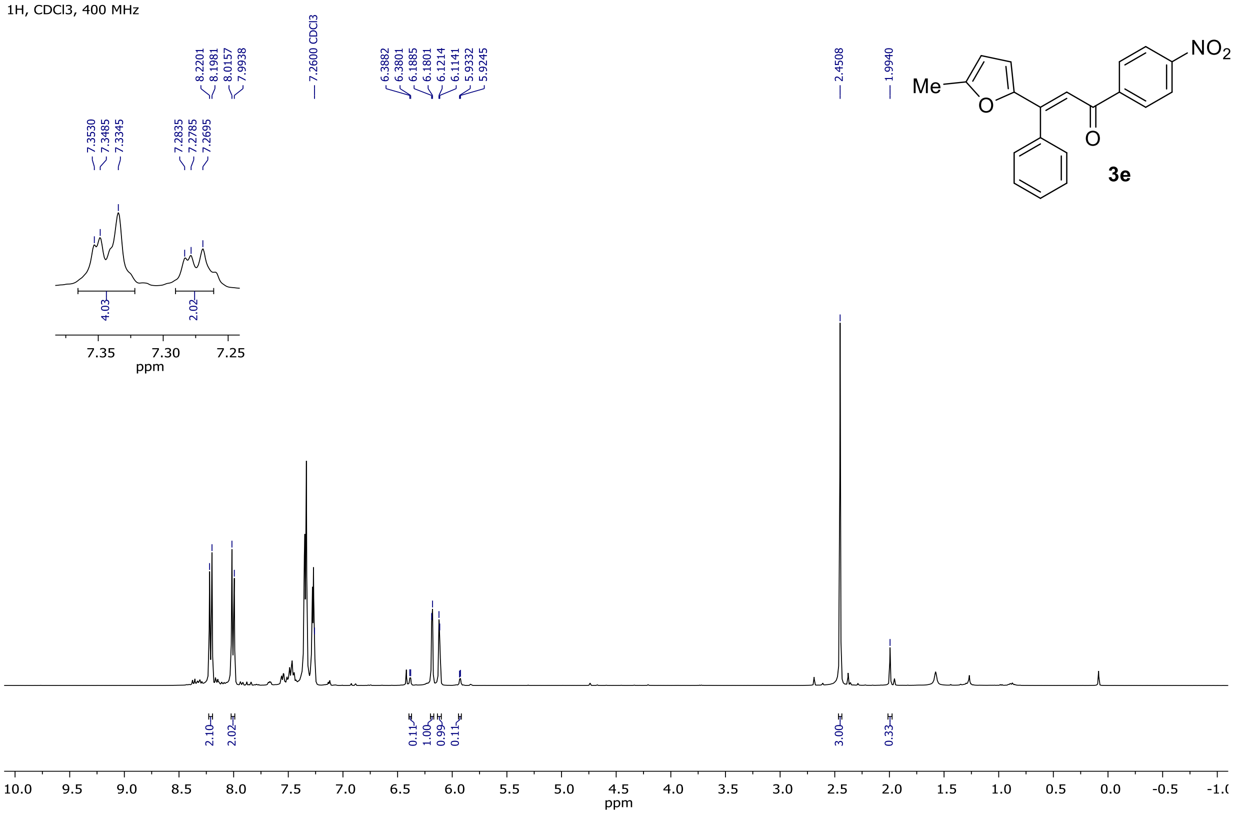

13C, CDCl3, 100 MHz

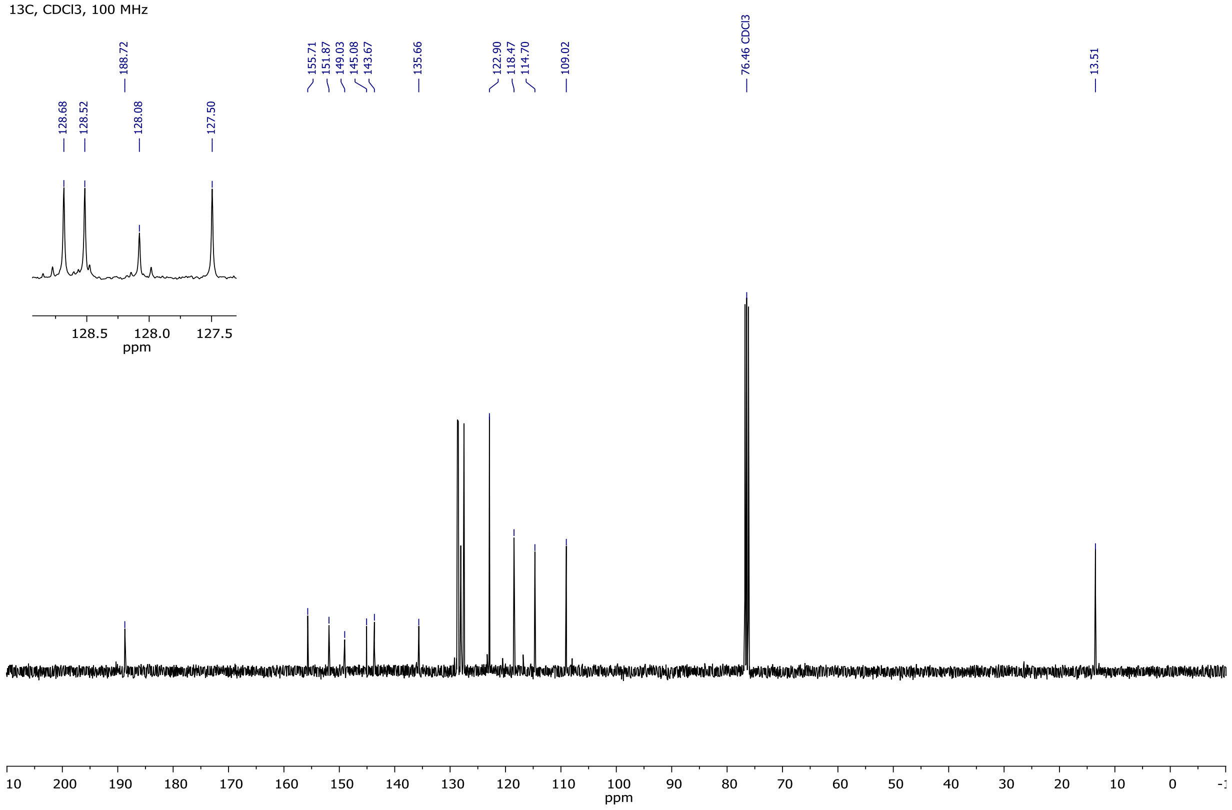

1H, CDCl3, 400 MHz

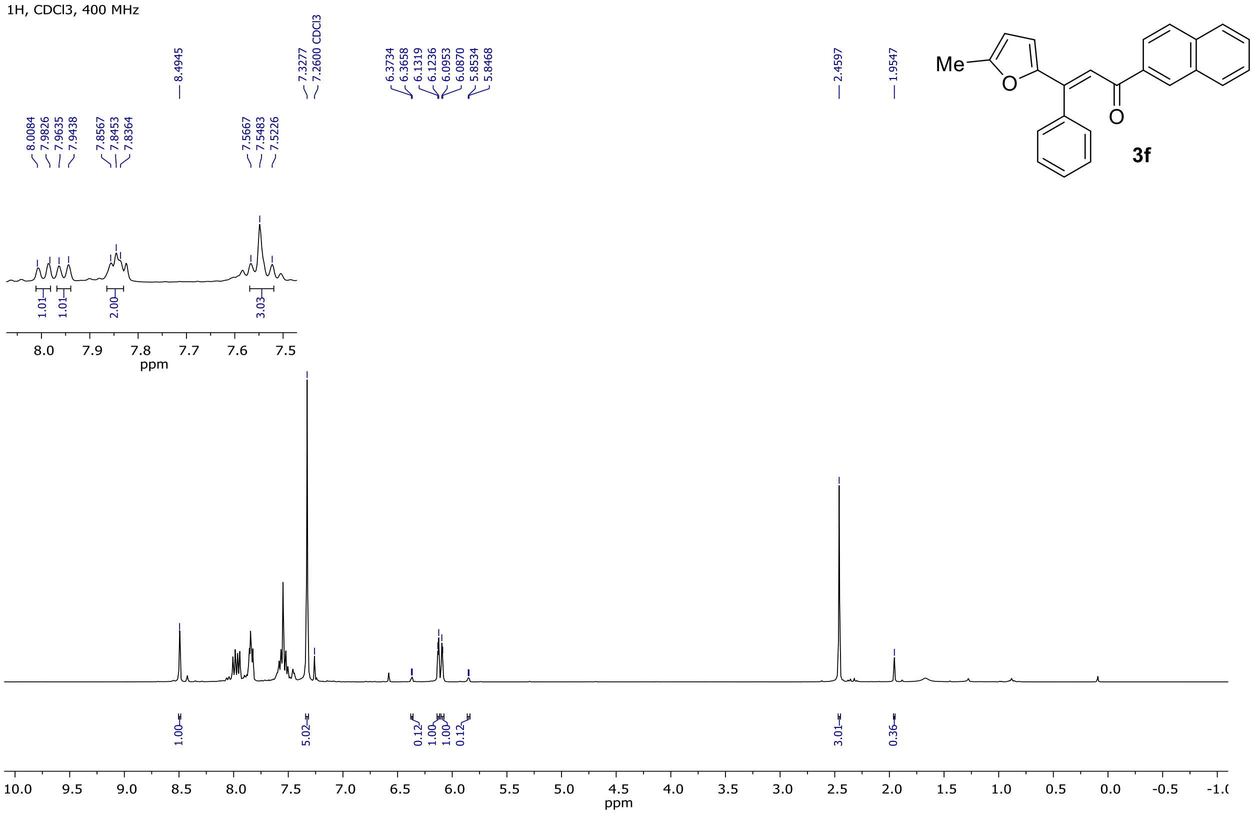

3f

13C, CDCl3, 100 MHz

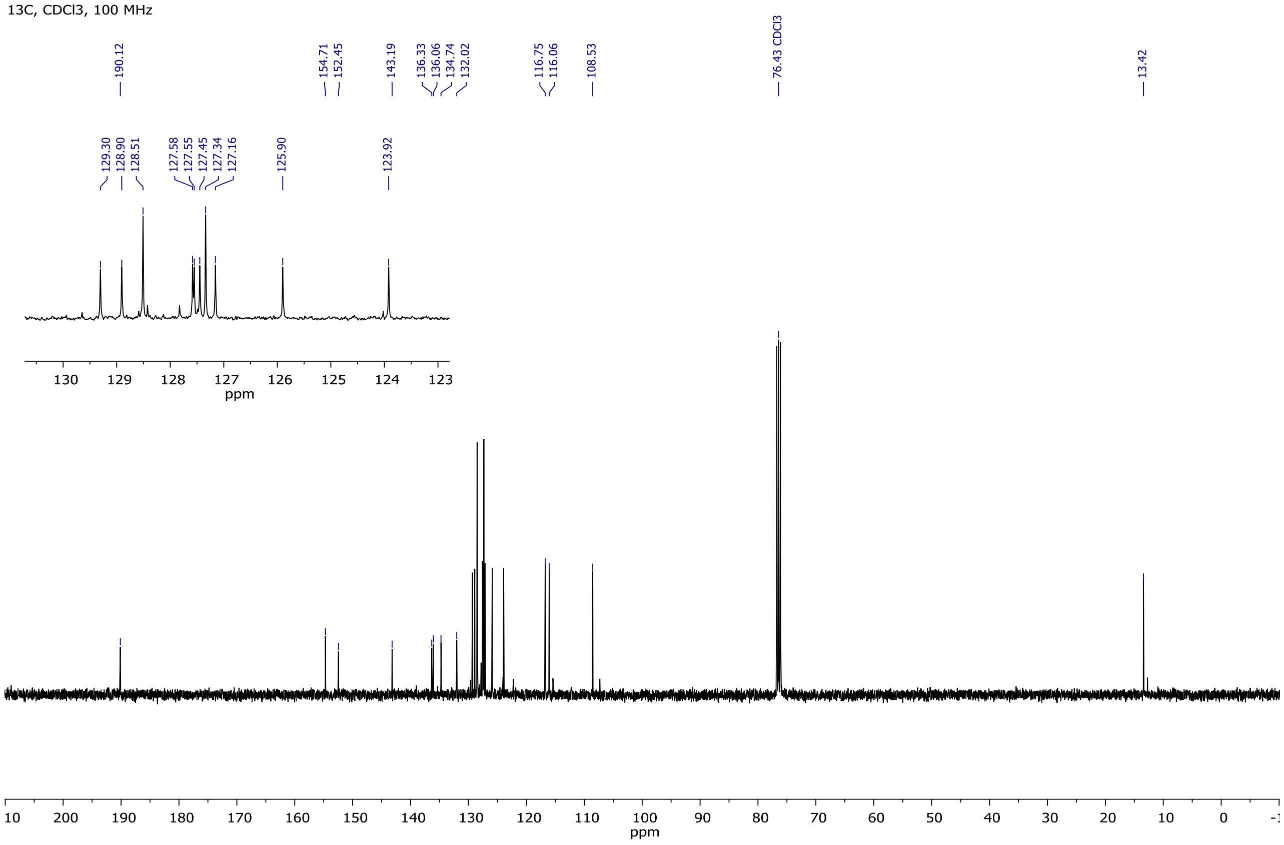

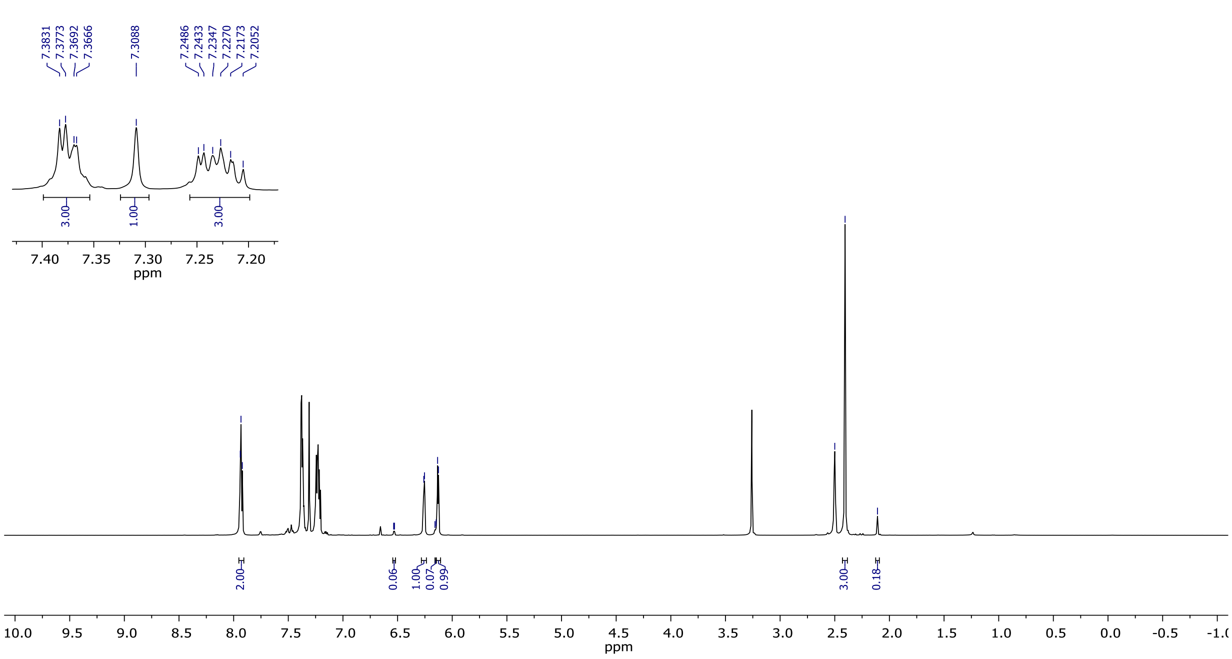
1H, DMSO-d6, 400 MHz
7.9393 7.9328 7.9207
6.5360 6.5284 6.2601 6.2539 6.1578 6.1471 6.1339 6.1257
2.5000 DMSO-d6 2.4055
2.1092
7.3831 7.3773 7.3692 7.3666
7.3088
7.2486 7.2433 7.2347 7.2270 7.2173 7.2052
3.00 1.00 3.00
7.40 7.35 7.30 7.25 7.20
ppm
2.00 0.06 1.00 0.07 0.99 3.00 0.18
10.0 9.5 9.0 8.5 8.0 7.5 7.0 6.5 6.0 5.5 5.0 4.5 4.0 3.5 3.0 2.5 2.0 1.5 1.0 0.5 0.0 -0.5
ppm

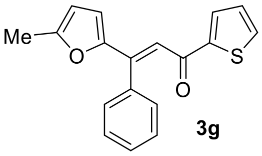
Me
O
S
O

3g

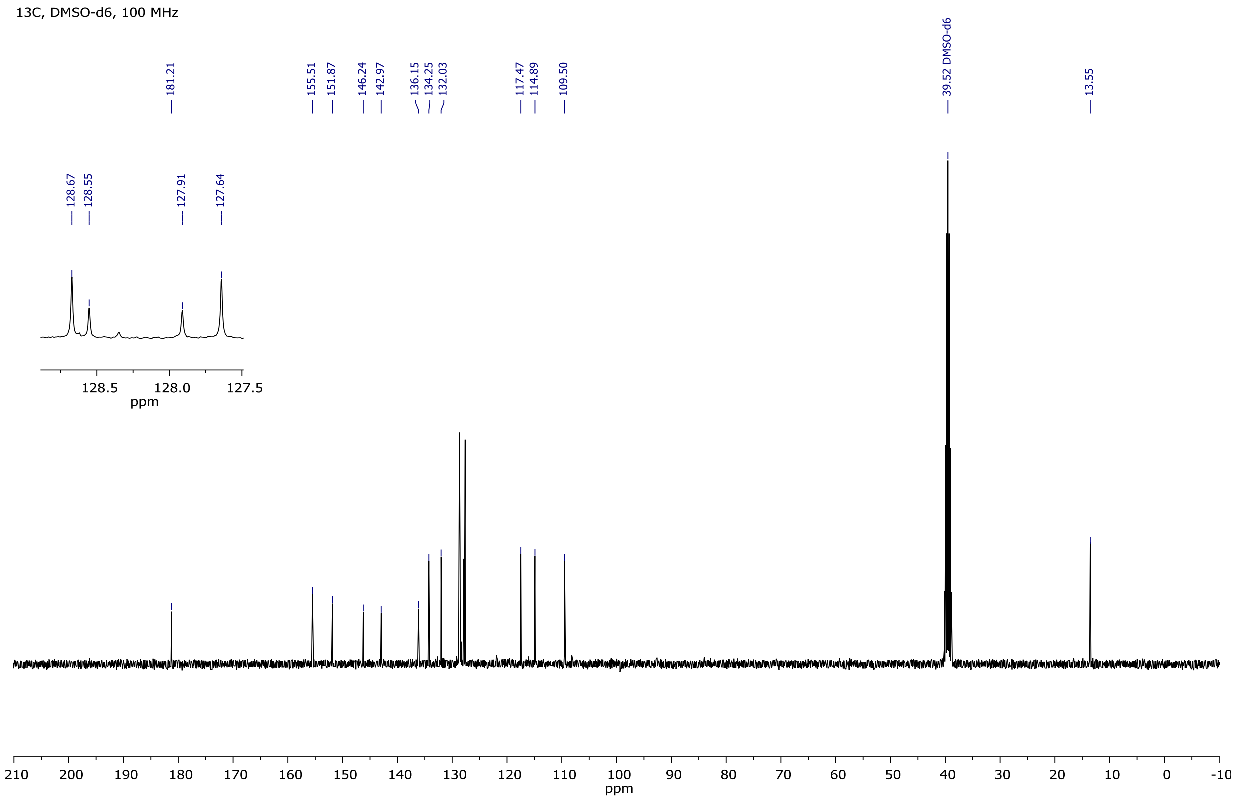
13C, DMSO-d6, 100 MHz
181.21
155.51 151.87 146.24 142.97 136.15 134.25 132.03
117.47 114.89 109.50
39.52 DMSO-d6
13.55
128.67 128.55 127.91 127.64
128.5 128.0 127.5
ppm
210 200 190 180 170 160 150 140 130 120 110 100 90 80 70 60 50 40 30 20 10 0 -10
ppm

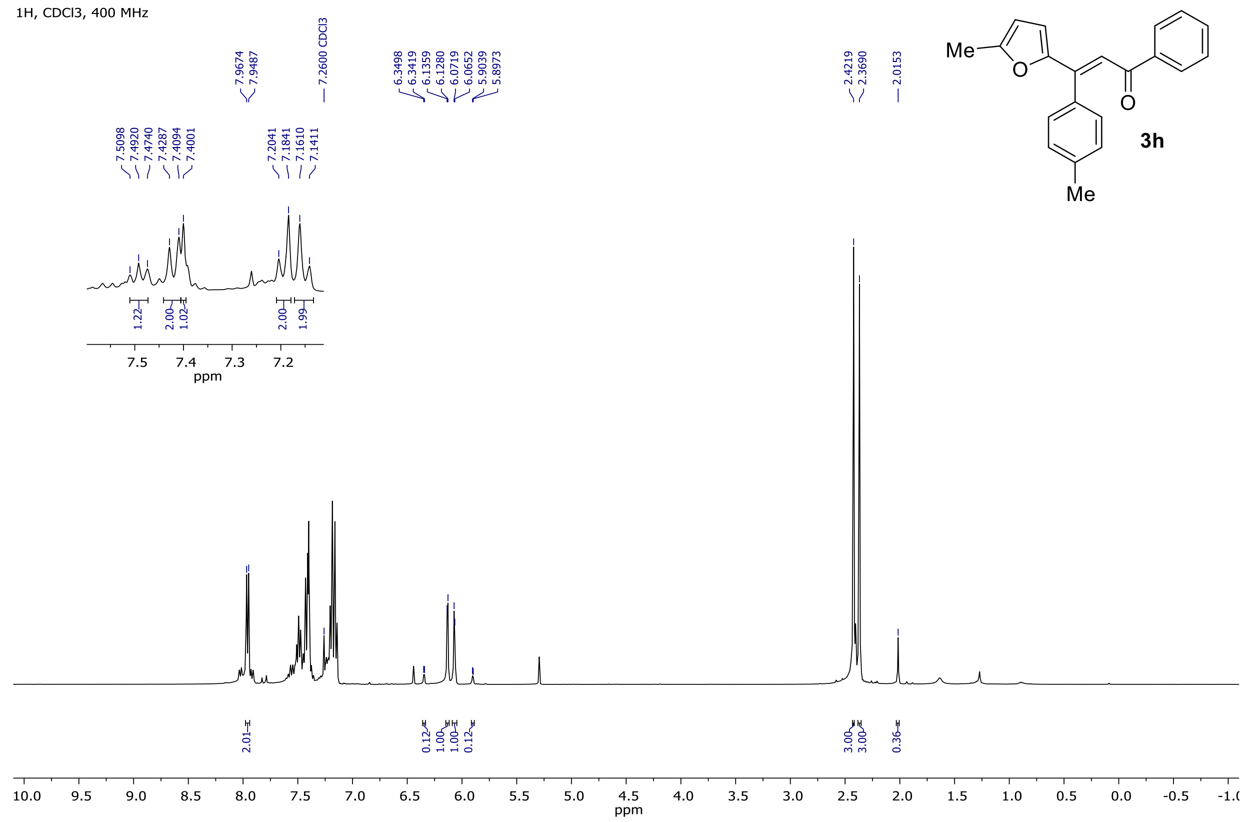
1H, CDCl3, 400 MHz
3h
Me
O
ppm

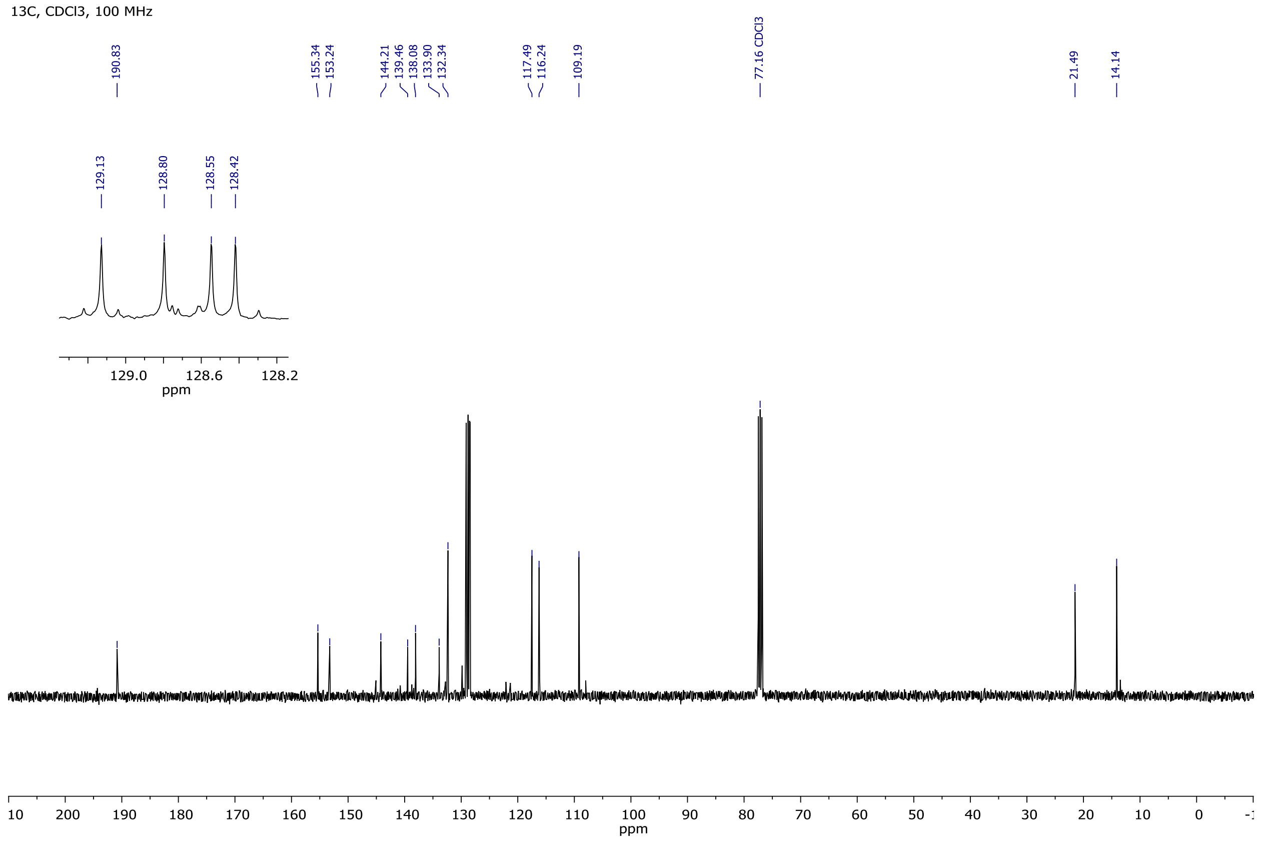
13C, CDCl3, 100 MHz
ppm

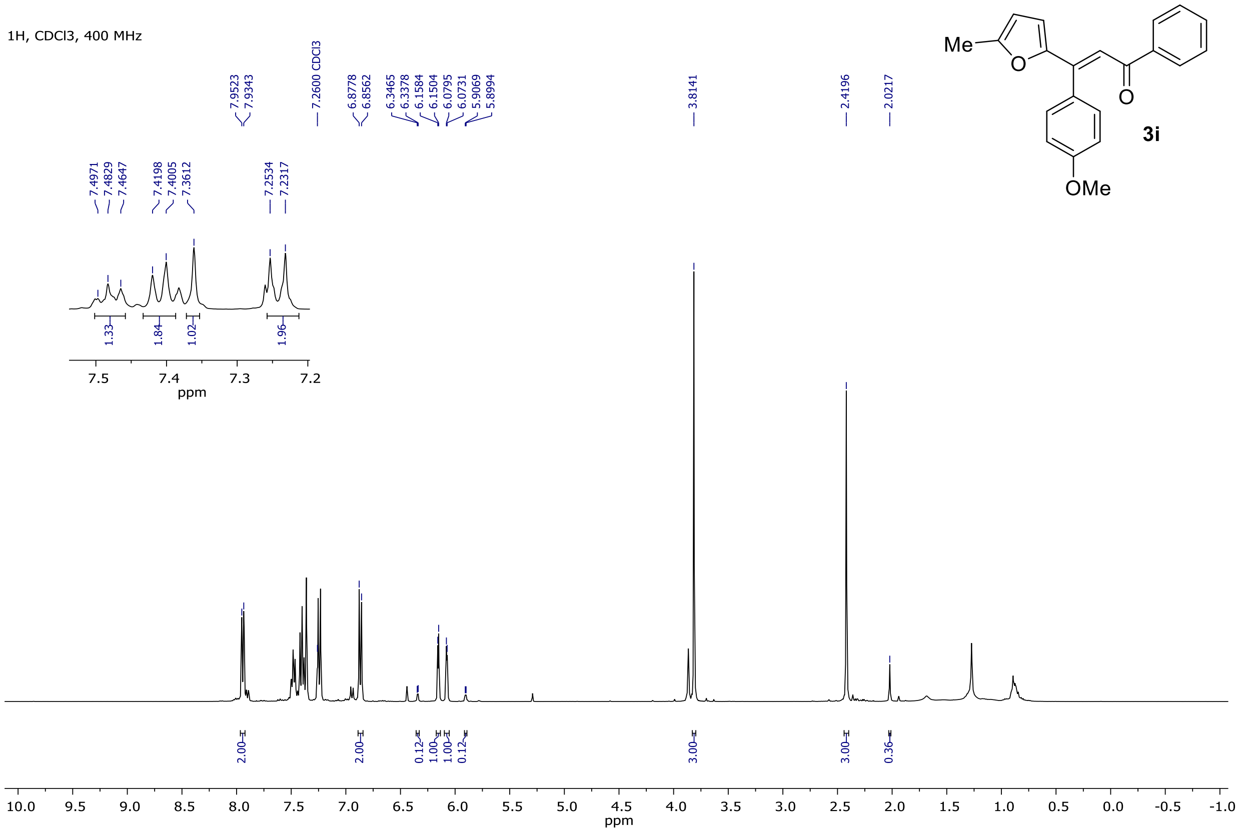
1H, CDCl3, 400 MHz
7.9523
7.9343
7.2600 CDCl3
6.8778
6.8562
6.3465
6.3378
6.1584
6.1504
6.0795
6.0731
5.9069
5.8994
3.8141
2.4196
2.0217
7.4971
7.4829
7.4647
7.4198
7.4005
7.3612
7.2534
7.2317
ppm
Me
O
O
OMe
3i

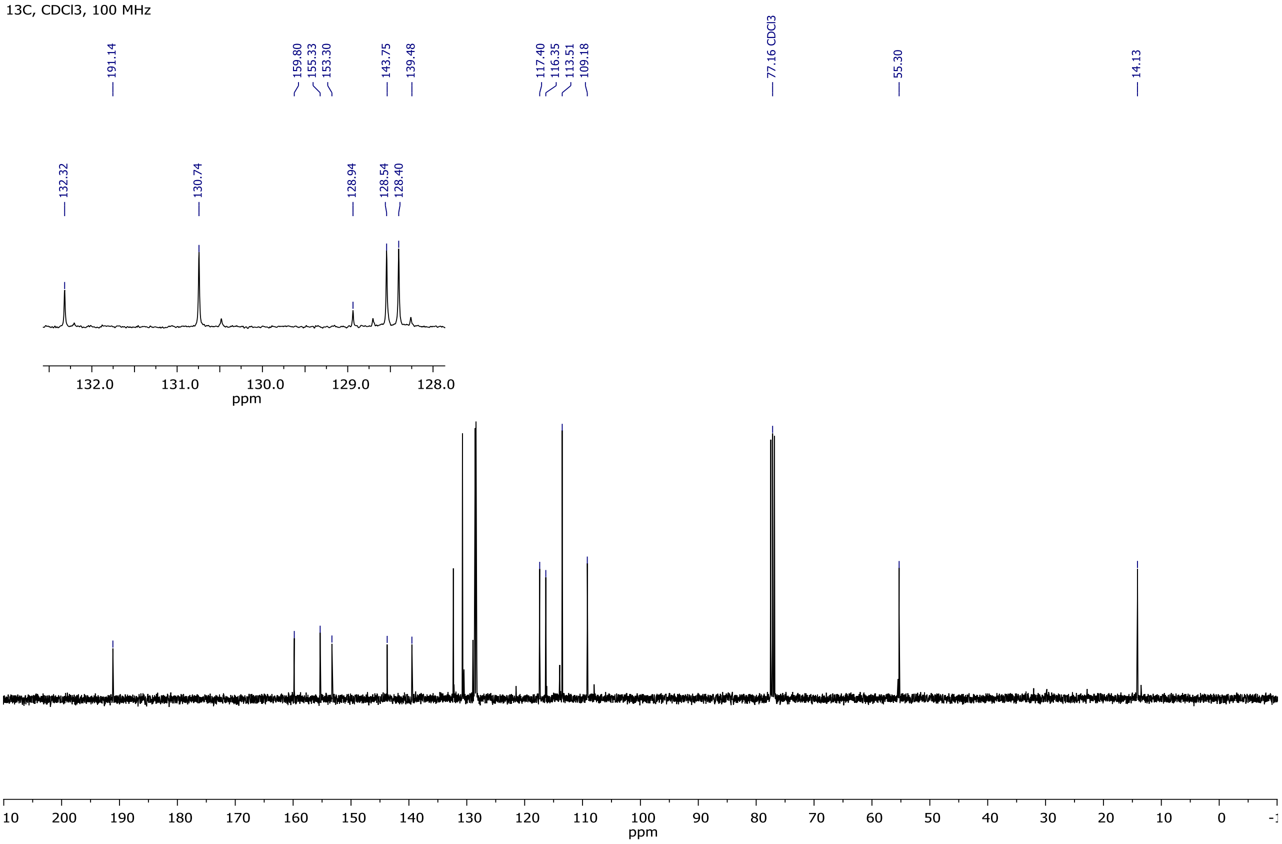
13C, CDCl3, 100 MHz
191.14
159.80
155.33
153.30
143.75
139.48
117.40
116.35
113.51
109.18
77.16 CDCl3
55.30
14.13
132.32
130.74
128.94
128.54
128.40
ppm

1H, CDCl3, 400 MHz

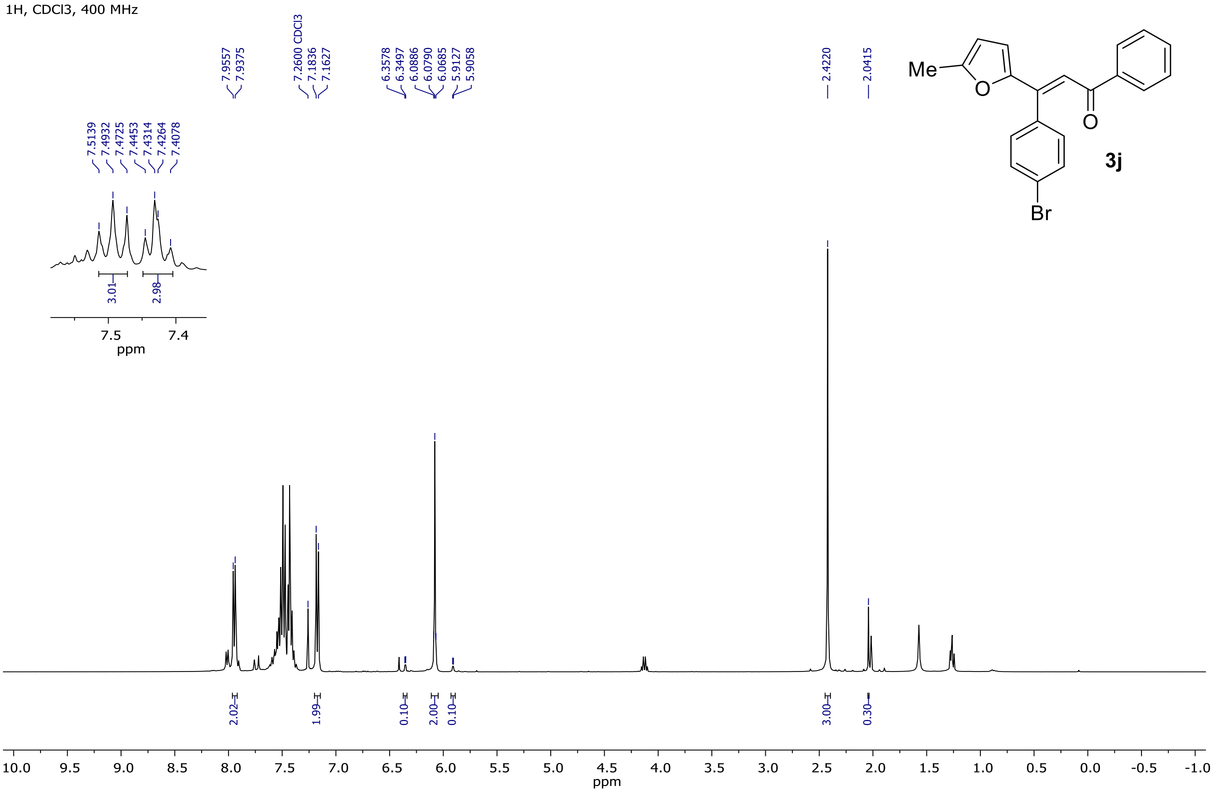

13C, CDCl3, 100 MHz

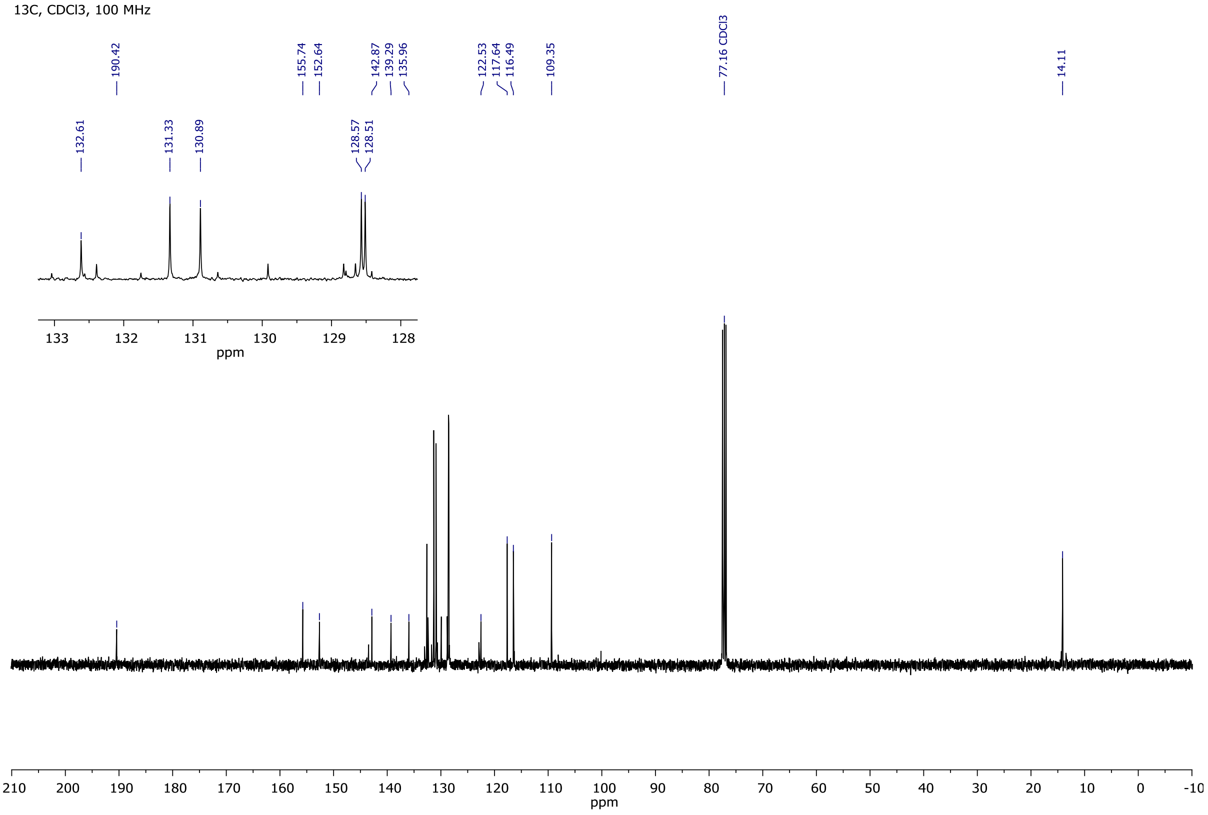

1H, CDCl3, 400 MHz

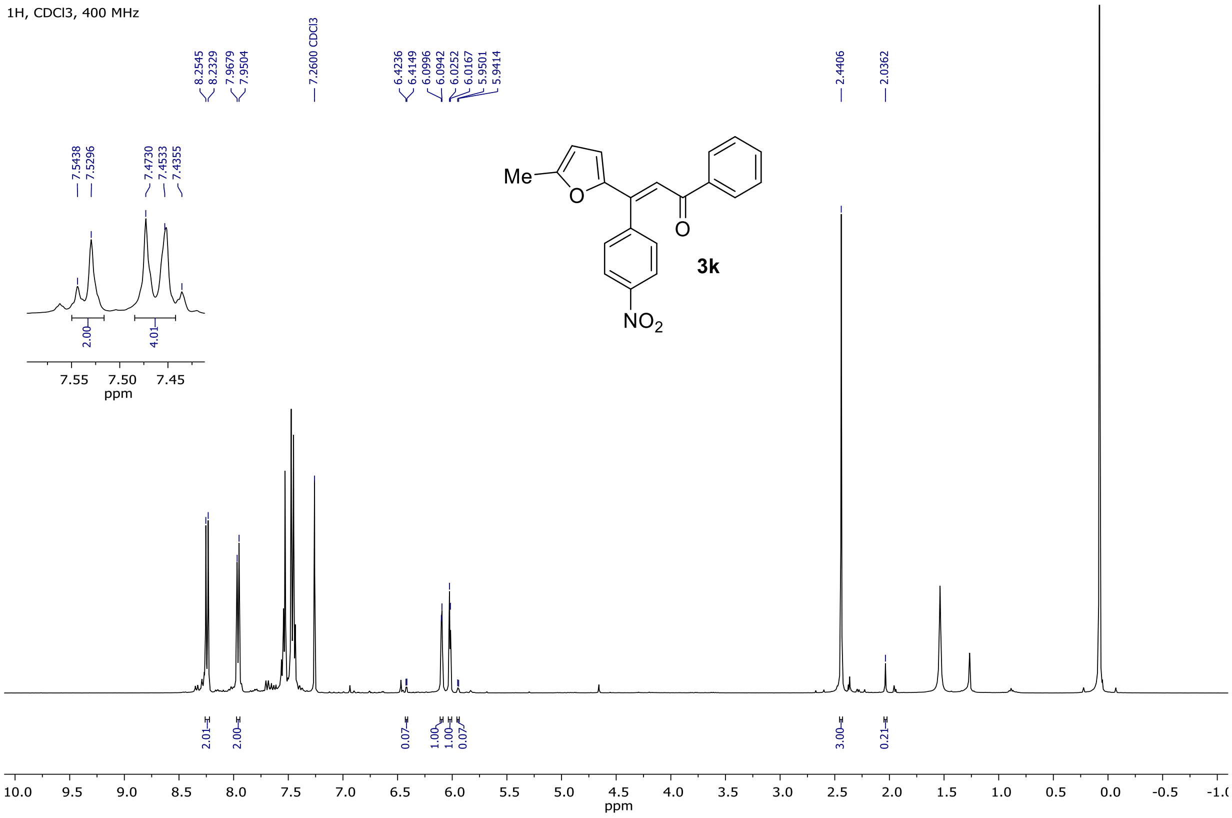

13C, CDCl3, 100 MHz

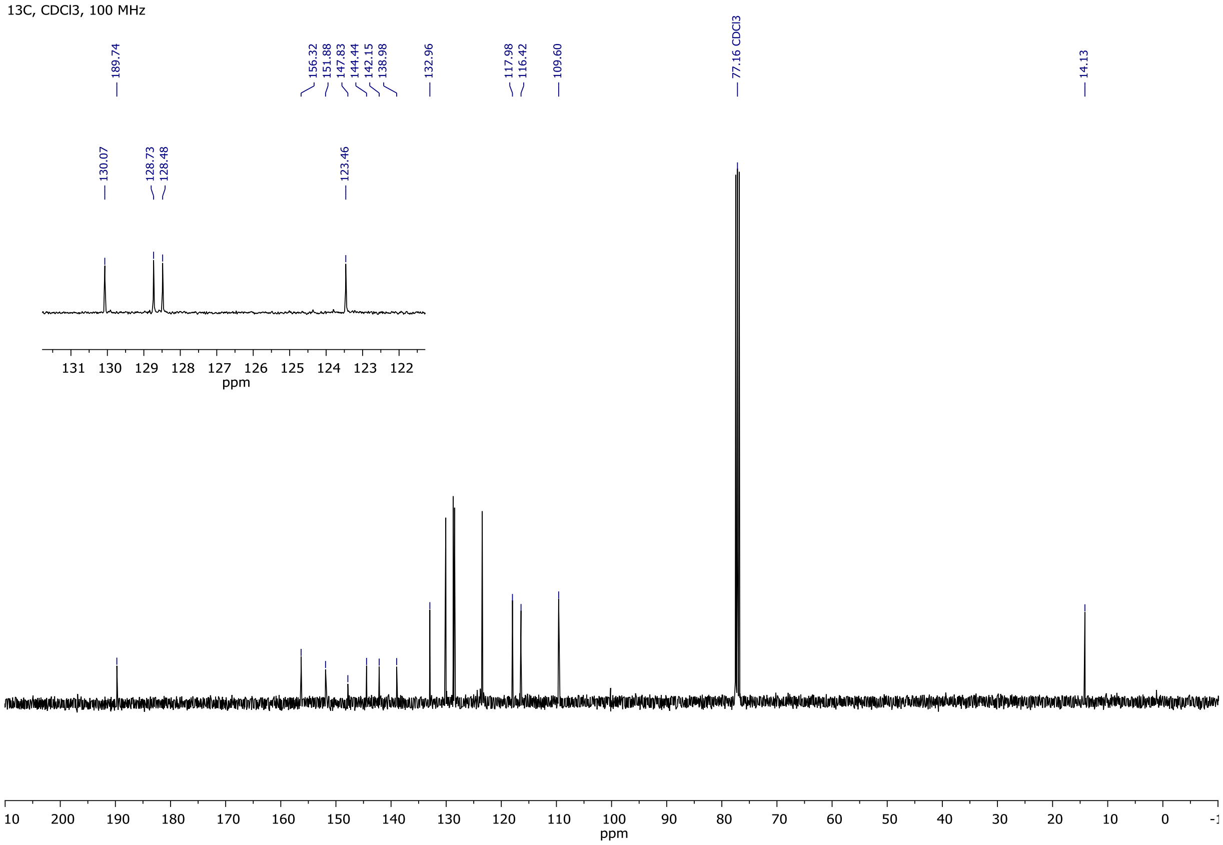

1H, DMSO-d6, 400 MHz

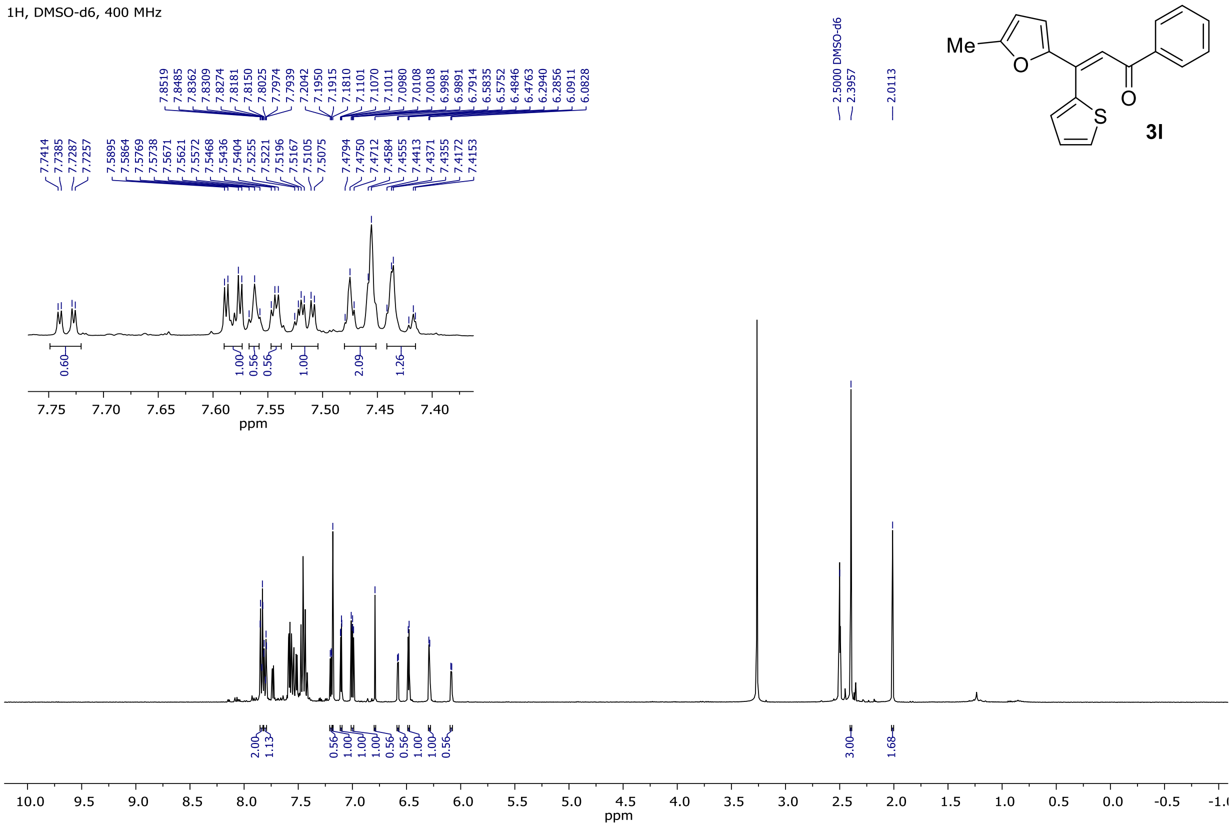

3l

13C, DMSO-d6, 100 MHz

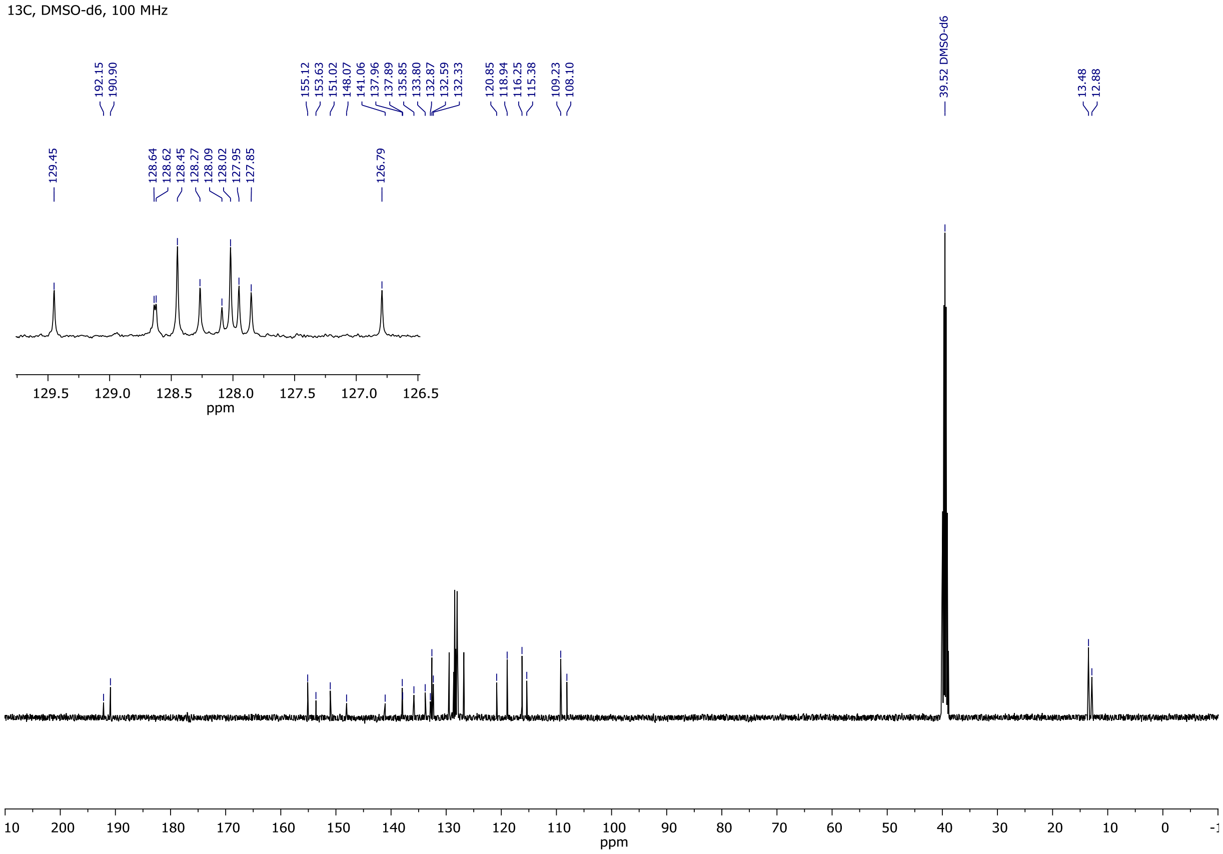

1H, CDCl3, 400 MHz

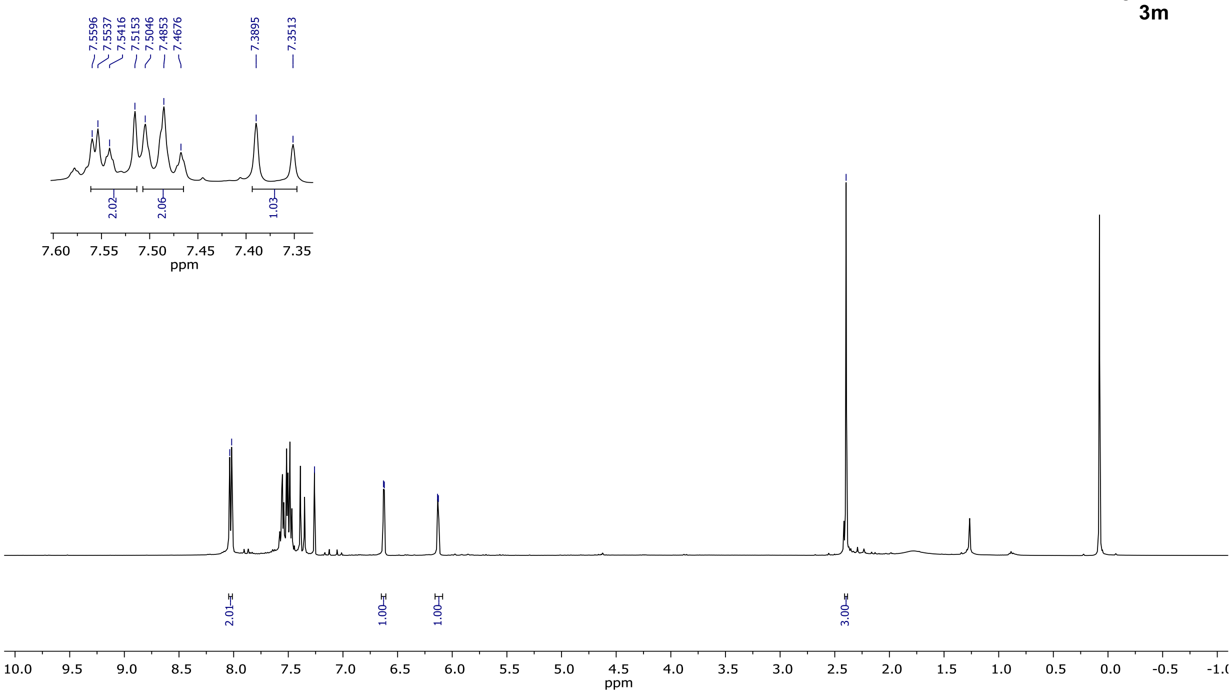

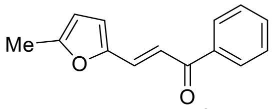

3m

13C, CDCl3, 100 MHz

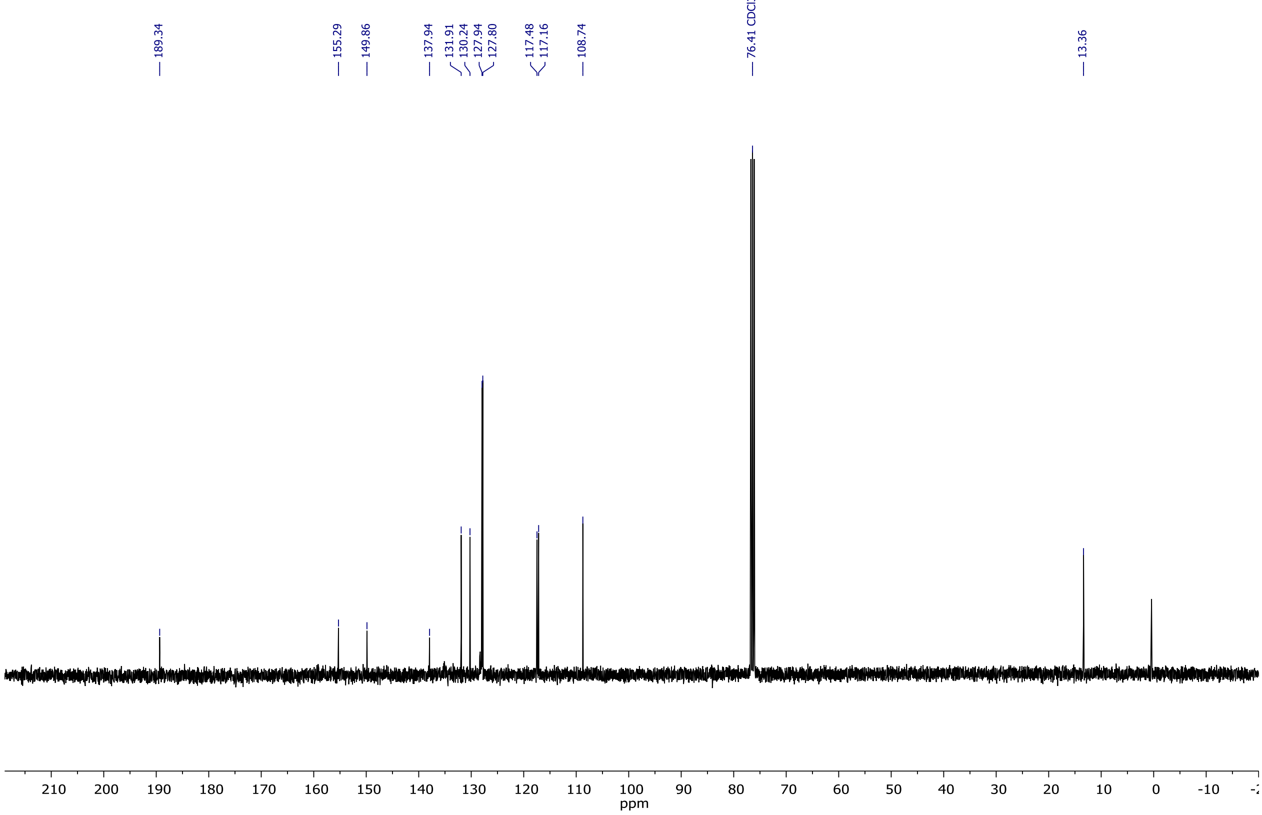

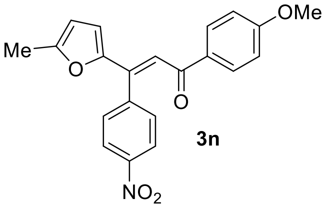
Me
O
OMe
O
NO2

3n

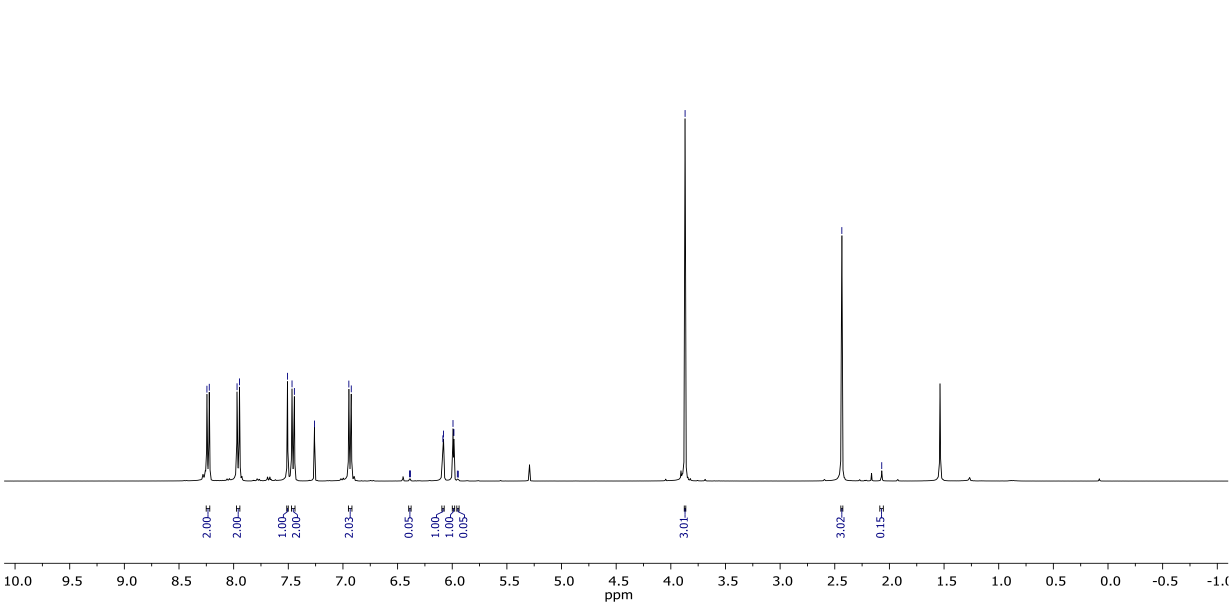
1H, CDCl3, 400 MHz
8.2446
8.2229
7.9689
7.9467
7.5073
7.4659
7.4442
7.2600 CDCl3
6.9460
6.9238
6.3908
6.3826
6.0856
6.0800
5.9931
5.9847
5.9530
5.9446
3.8695
2.4354
2.0703
2.00
2.00
1.00
2.00
2.03
0.05
1.00
1.00
0.05
3.01
3.02
0.15
ppm

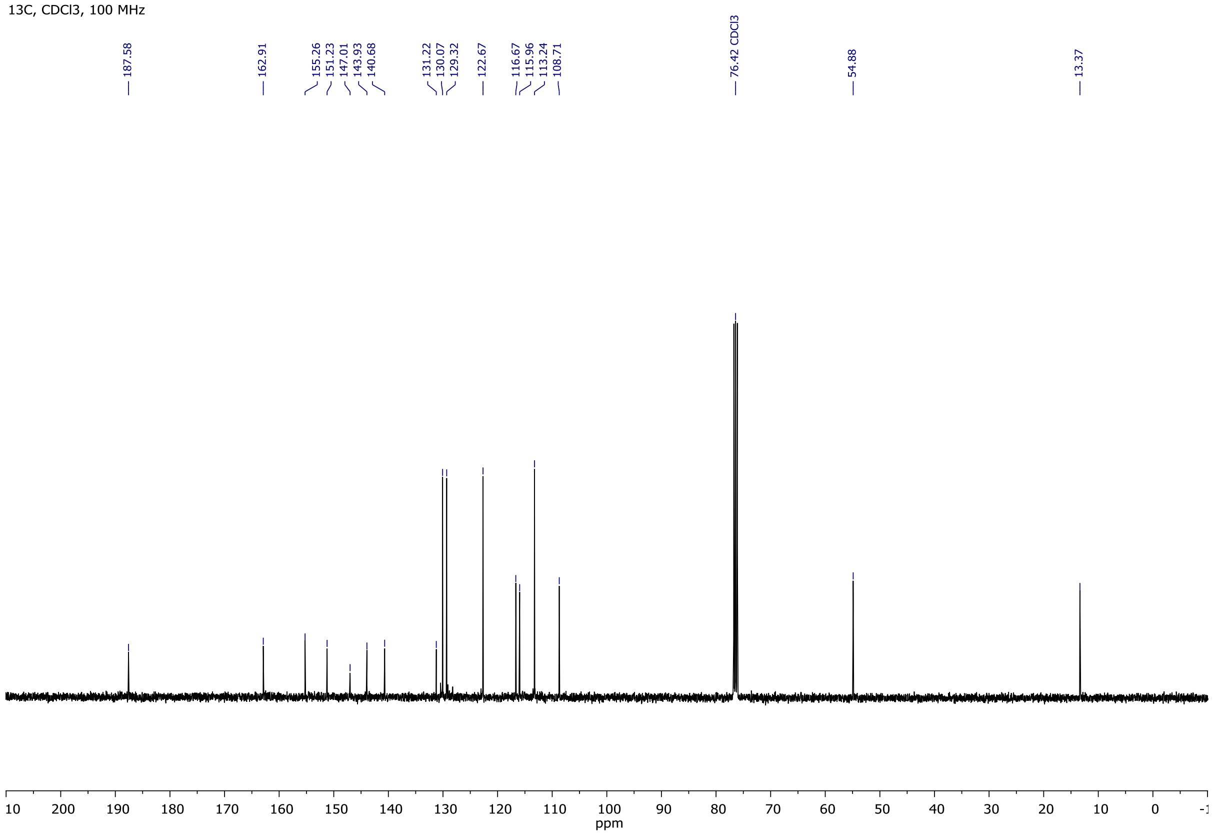
13C, CDCl3, 100 MHz
187.58
162.91
155.26
151.23
147.01
143.93
140.68
131.22
130.07
129.32
122.67
116.67
115.96
113.24
108.71
76.42 CDCl3
54.88
13.37
ppm

1H, CDCl3, 400 MHz

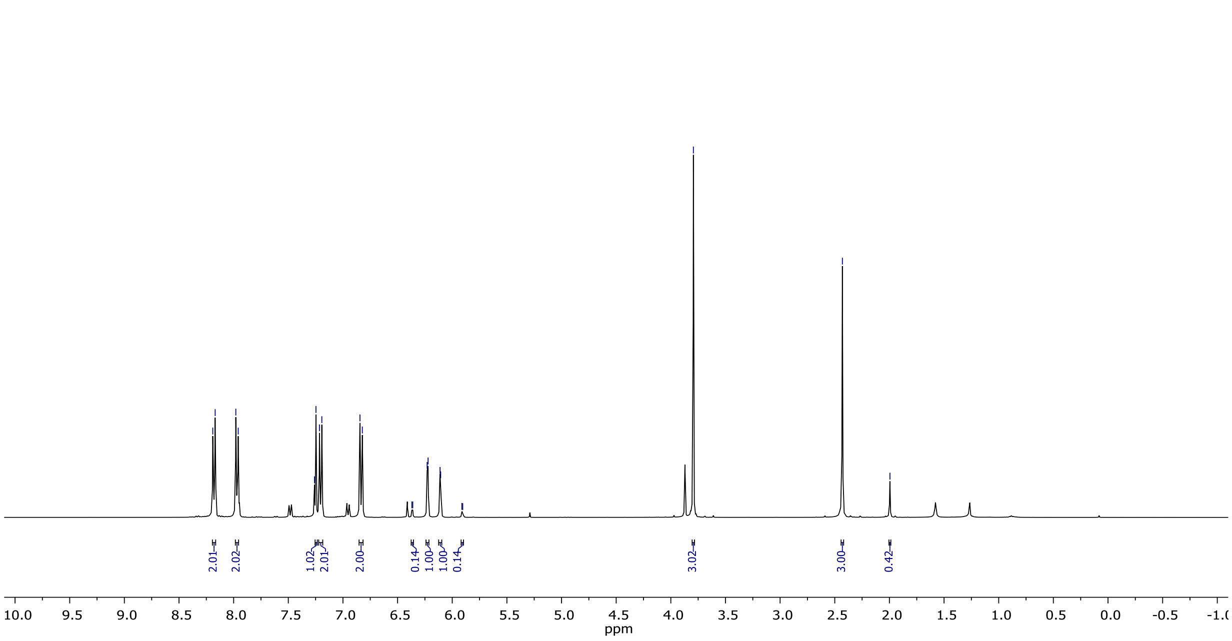

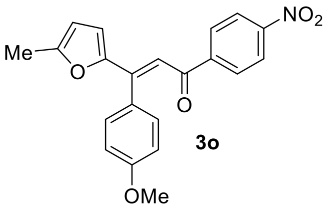

3o

13C, CDCl3, 100 MHz

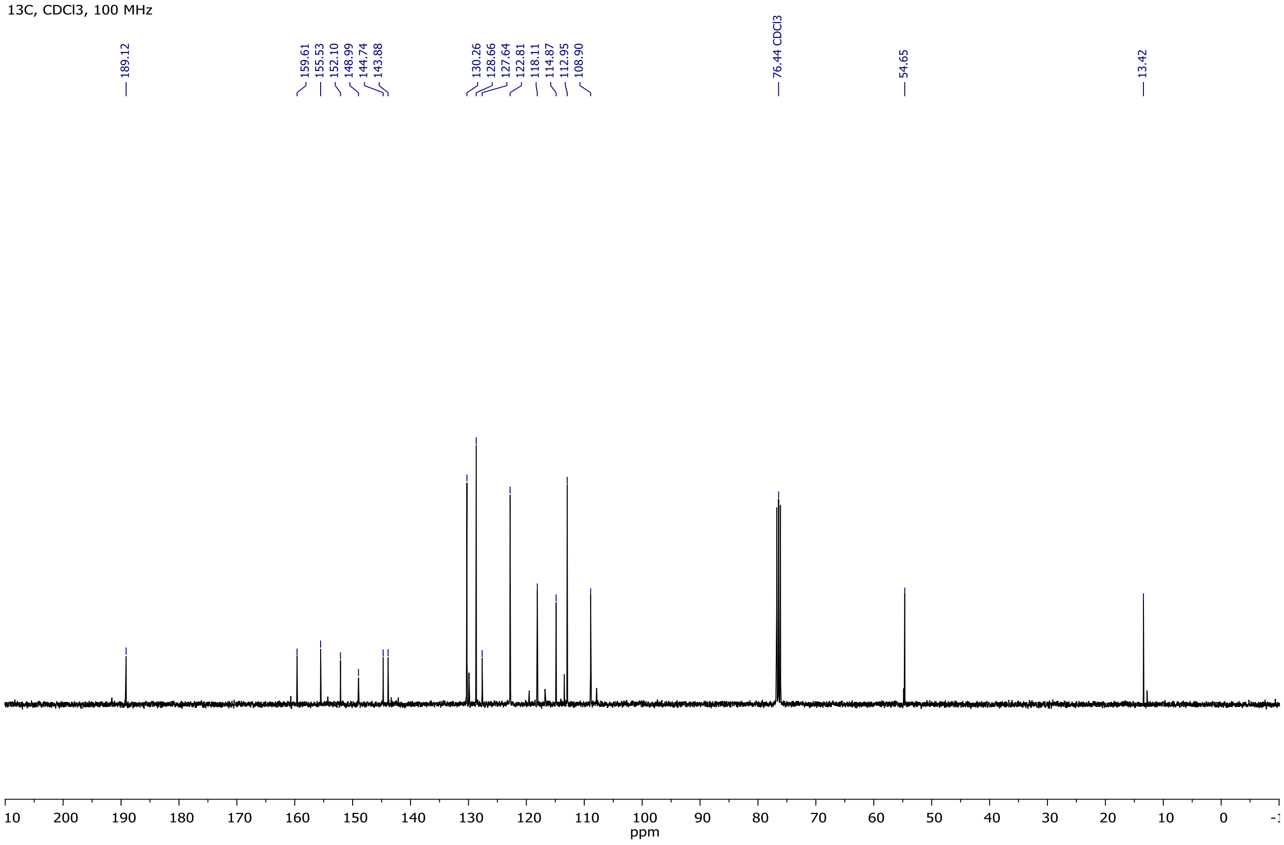

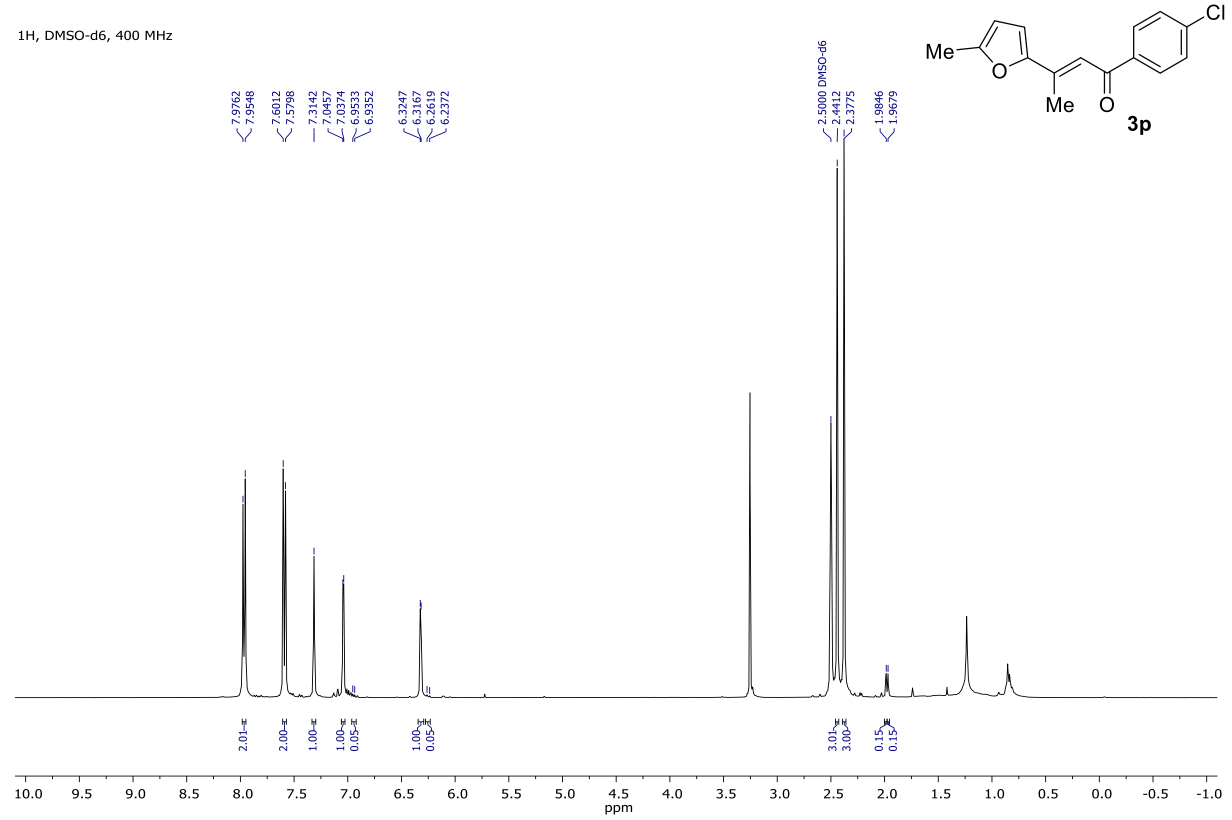
1H, DMSO-d6, 400 MHz
7.9762
7.9548
7.6012
7.5798
7.3142
7.0457
7.0374
6.9533
6.9352
6.3247
6.3167
6.2619
6.2372
2.5000 DMSO-d6
2.4412
2.3775
1.9846
1.9679
Me
O
Me
O
Cl
3p
2.01
2.00
1.00
1.00
0.05
1.00
0.05
3.01
3.00
0.15
0.15
10.0
9.5
9.0
8.5
8.0
7.5
7.0
6.5
6.0
5.5
5.0
4.5
4.0
3.5
3.0
2.5
2.0
1.5
1.0
0.5
0.0
-0.5
-1.0
ppm

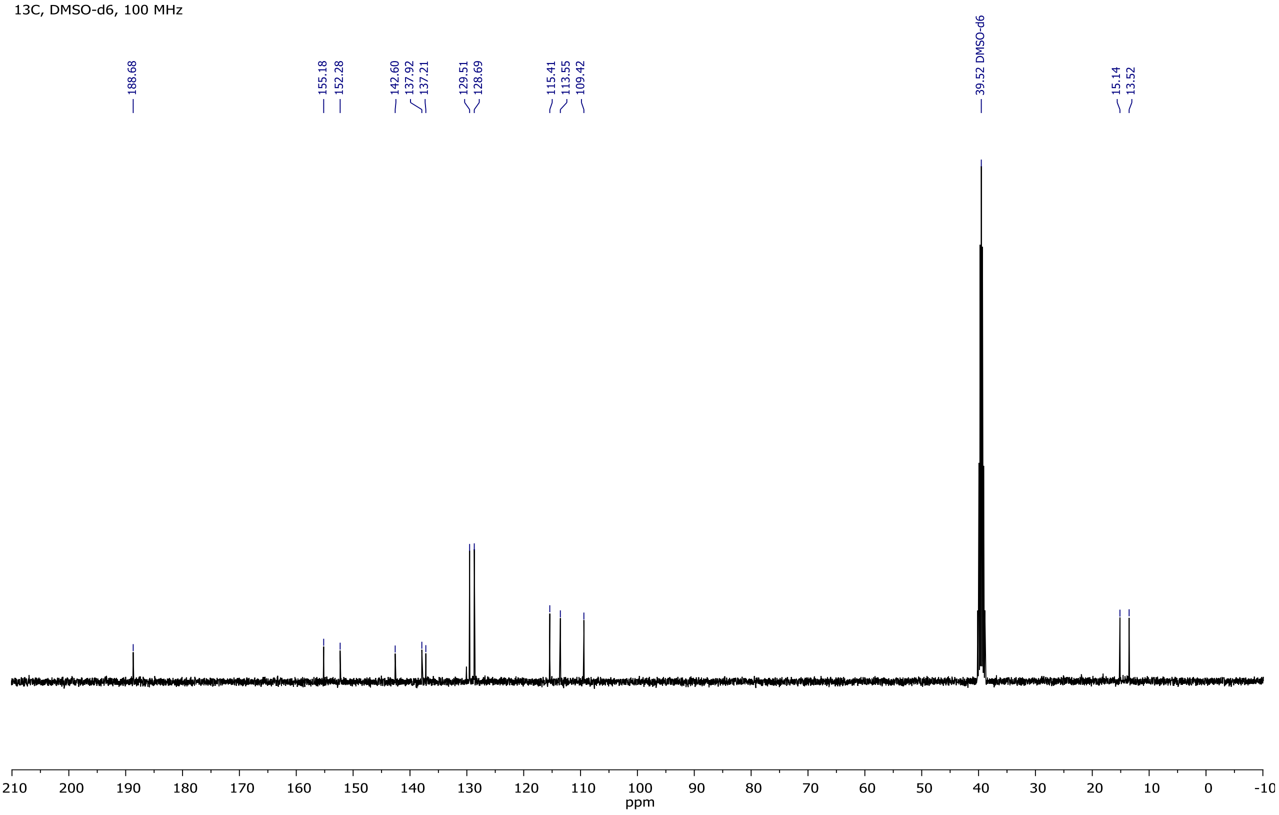
13C, DMSO-d6, 100 MHz
188.68
155.18
152.28
142.60
137.92
137.21
129.51
128.69
115.41
113.55
109.42
39.52 DMSO-d6
15.14
13.52
210
200
190
180
170
160
150
140
130
120
110
100
90
80
70
60
50
40
30
20
10
0
-10
ppm
